# Conventional versus task-based package organization for out-of-hospital emergency kits: an emergency medical services simulation study

**DOI:** 10.1186/s13049-024-01309-8

**Published:** 2024-12-20

**Authors:** Daniel Laxar, Daniel Grassmann, Lena Reischmann, Alexandra Kaider, Bertram Schadler, Carmen Huber, Mario Krammel, Christina Hafner

**Affiliations:** 1 Medical University of Vienna, Department of Anaesthesia, Intensive Care Medicine and Pain Medicine, Division of General Anaesthesia and Intensive Care Medicine, Spitalgasse 23, 1090 Vienna, Austria; 2 Ludwig Boltzmann Institute Digital Health and Patient Safety, Währinger Straße 104/10, 1180 Vienna, Austria; 3 Emergency Medical Service Vienna, Radetzkystraße 1, 1030 Vienna, Austria; 4 Medical University of Vienna, Center for Medical Data Science, Institute of Clinical Biometrics, Spitalgasse 23, 1090 Vienna, Austria

**Keywords:** Emergency kit, Backpack, Equipment retrieval, Prehospital emergency medicine, Emergency medical services

## Abstract

**Background:**

Emergency Medical Service crews are equipped with comprehensive emergency kits for routine care and to provide life-saving interventions in severely ill patients. While guidelines on contents and packing strategies of emergency kits for specific tasks and specialized situations exist, data for the design of out-of-hospital emergency kits in a general urban population is lacking. It may be possible to transfer the promising results of modern in-hospital packing strategies such as task-based package organization (TPO) to an Emergency Medical Service setting.

**Methods:**

Four types of emergency kit were used in this study: two novel backpack emergency kits were acquired for this study (one packed using a TPO approach (PAX bags) and the other a traditional non-TPO approach (inter-rescue)); the existing emergency kit; and a TPO-repack of the existing kit. We recruited 80 paramedics who each performed four different tasks in a simulated setting: preparation for endotracheal intubation; preparing an intravenous access and a crystalloid infusion; preparing intraosseous access with medication; and preparing for a forearm splint. Questionnaires were completed before starting, after each task, and at the end of each study session.

**Results:**

There was no overall difference for the primary outcome of task completion between the novel TPO and novel non-TPO kit (p = 0.11). However, for selected tasks (forearm splint) completion time was significantly different between these kits. Overall, participants performed fastest when using the existing emergency kit. Participants frequently omitted items required for critical procedures, regardless of kit type.

**Conclusion:**

TPO has been previously investigated in an in-hospital setting using participants with low exposure to medical emergencies, and with promising results. In our prehospital simulation setting with paramedics, equipment retrieval was neither faster nor more complete compared to non-TPO kits.

**Supplementary Information:**

The online version contains supplementary material available at 10.1186/s13049-024-01309-8.

## Introduction

In the City of Vienna, Austria, prehospital emergency care is routinely provided by the communal Emergency Medical Service Vienna that works with two-paramedic ambulance teams who respond to a vide variety of calls. For patients in critical condition, additional emergency physician response vehicles are dispatched. EMS crews must be able to prepare the equipment required for emergency procedures, to assist a physician at the scene, and, if qualified, to perform life saving procedures themselves where no physician is available.

The type and extent of equipment available to out-of-hospital emergency teams is continuously changing [1] and varies between emergency care systems [1–3]. In contrast to in-hospital emergency settings, crews need to carry equipment for routine calls as well as life-threatening emergencies such as cardiac arrest. Thus, portable and comprehensive emergency kits are a mainstay of prehospital emergency care. A study from the US found that the most common procedures are cardiopulmonary resuscitation with defibrillation; airway management; vascular access; immobilization; as well as splinting and hemorrhage control [4].

Traditional emergency kits are often organized using either an ABC-approach (i.e., airway, breathing, circulation) or by material type (i.e., all medication in one place). However, observations of in-hospital clinical emergency crews have shown that consideration of human factors improves acceptance and performance of crash carts [5, 6]. More recently, a specific design principle of emergency kits has been formally summarized as task-based package organization (TPO) [7, 8]. TPO follows the paradigm of closely storing together all items necessary for a specific task. For instance, obtaining intravenous access would require just one emergency kit module containing all the equipment necessary to perform the procedure [7]. Moreover, the module contains no extraneous items, thus reducing the overall number of items. The aim is to reduce potential distractions for healthcare professionals subject to a high working memory load [9, 10].

While TPO and other novel approaches to kit organization have been described successfully in an in-hospital setting [8, 11, 12], research on prehospital emergency kit contents and organization remains scarce.

The aim of this study was to investigate the impact of TPO in prehospital emergency kits compared to traditional package organization by conducting simulation experiments with 80 participants. The participants were each required to use four different emergency kits with which to accomplish four different emergency procedures. The primary outcome was the overall equipment retrieval time between the two novel kits (novel TPO versus novel non-TPO).

## Methods

The study is a prospective simulation study and complies with the Declaration of Helsinki. The study was performed as a cooperation between the Medical University of Vienna, the Ludwig Boltzmann Institute Digital Health and Patient Safety, and the Emergency Medical Service Vienna. The institutional review board of the Medical University of Vienna waived the need for ethics committee approval in September 2023. Participants provided written informed consent in advance, in digital form, using the REDCap eConsent framework [13]. All data was collected using the REDCap electronic data capture tool hosted at the Medical University of Vienna [14, 15]. Data was anonymized prior to analysis.

Paramedics who regularly use the current emergency kit and were above the age of 18 were eligible to participate. We did not recruit emergency physicians as they are assisted by paramedics who will handle the equipment during calls. Exclusion criteria were previous knowledge of the study aims, previous exposure to any of the new emergency kits, and wearing high prescription glasses (≥ 6 diopters).

**Fig. 1:**
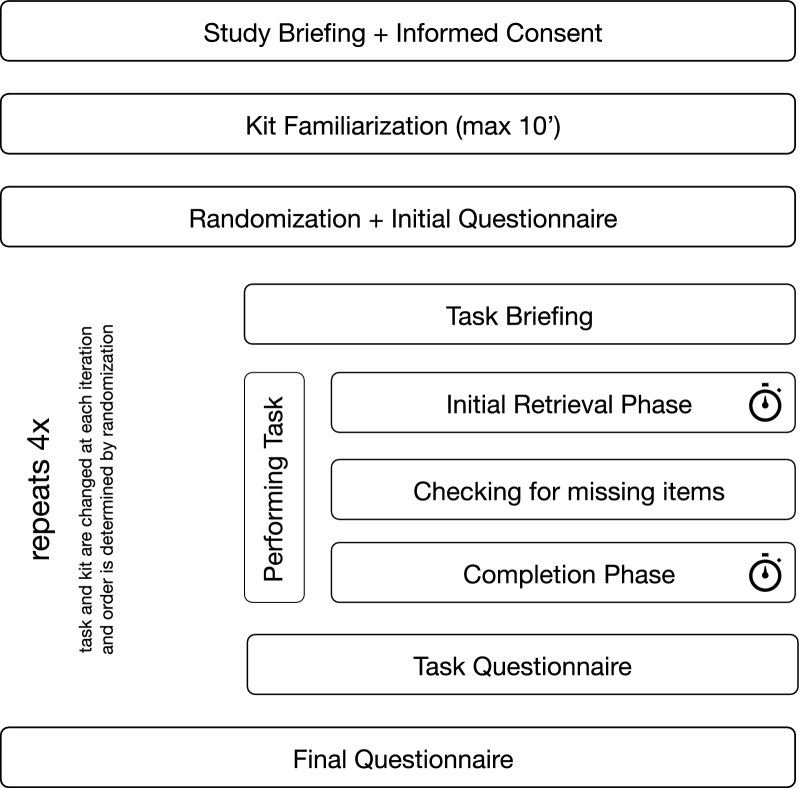
Study proceedings. Steps with clock symbols counted towards the total retrieval time

Working paramedics were recruited in November and December of 2023. After obtaining consent and brief familiarization, participants were given an initial questionnaire, four tasks with four respective questionnaires, as well as a final questionnaire at the end of the simulation (all questionnaires available in the supplementary materials). Emergency kit and task were randomized in blocks of 16, ensuring each combination of kit and task was equally common. For each task, the participant was given a brief description of what should be accomplished. A task consisted of retrieving all the items necessary for an emergency procedure to assist an on-site physician. Once the participant had successfully retrieved all required items and placed them in a pre-defined area, the time was stopped. Where items were missing, the participant was informed and the clock restarted, to record the time needed to retrieve the missing items. Where the participant was unable to locate items, they announced this to the study team and were allowed to omit the item in question. Total time to completion was the sum of time for initial retrieval and time for subsequent retrieval for completion. Detailed study proceedings are displayed in Fig. 1.

The four tasks were selected based on the international literature [4] and retrospective analysis of mission documentation dating from 2018 through 2022. However, only tasks which are predominantly performed using emergency kits were considered for this study. We selected the following tasks: preparing for endotracheal intubation; intravenous access and crystalloid infusion; intraosseous access including medication; and a forearm splint. Task checklists were created by the EMS Vienna Quality Assurance team. The checklists were not shared with participants for the study purpose but include all items necessary to perform a task as per the EMS Vienna Standard Operating Procedures. The task checklists can be found in the supplementary materials. To avoid bias, task selection and emergency kit design were performed by separate teams.

Two new emergency kits—both backpacks—were obtained for this study. The PAX bag (PAX emergency bag P5/11 L 2.0, X-CEN-TEK GmbH & Co. KG, Germany) was packed using TPO (novel TPO). In contrast, the inter-rescue bag (inter-rescue Dragon Dualsystem Oxygen 37 compact, inter-rescue e.K., Germany) was organized using a more traditional approach (novel non-TPO). The ambulance service had not previously used either type of emergency kit. Additionally, the existing emergency kit (Söhngen PROFiL, W. Söhngen GmbH, Germany), as well as a TPO-modified version, were also used in the simulation, providing a total of four different emergency kits. Images and list of contents of all the kits are provided in the supplementary materials.

The primary outcome of this study was a comparison of these novel kits (novel TPO versus novel non-TPO) with respect to total time to completion. Time to completion was chosen as the primary outcome due to the relevance in clinical practice as tasks were designed for both time-sensivity and assisting an third party (e.g., emergency physician present at the scene). Secondary outcomes included the comparision of timing data between all four kits, the completeness of retrieval and association between perceived task performance and total retrieval time.

Relevant pilot studies were not available at the time of planning. We therefore conducted an internal pilot using the first sixteen participants (i.e., one randomization block) to estimate the variance of the intra-participant difference, and to determine the required sample size to achieve 80% power with a two-sided probability of 0.05 of conducting a type I error, assuming that a 20% time difference was considered relevant. Sample size calculation revealed a required study size of 54 participants. As recruiting progressed very well, we decided to include 80 participants (5 blocks) to increase statistical power.

The analysis was blind with respect to the emergency kit used. Continuous variables are described by median values (interquartile range). A mixed linear model was used to evaluate the difference in the total time to completion between the four emergency kits, also including the task type and the period [1–4] as fixed factors and the participants as a random factor. The pairwise comparison between the novel TPO and the novel non-TPO kits was conducted as a post-hoc comparison to answer the primary research question. Furthermore, as a hypothesis-generating analysis, the interacting effect between the emergency kit and the task type was tested within the mixed linear model. Due to the right-skew distribution of the time to completion, log-transformed values were used for the analyses. Exploratory correlation analyses were performed to calculate the Spearman’s correlation coefficient. Two-sided p-values less than 0.05 were considered statistically significant. Statistical analyses were performed using SAS version 9.4 (SAS Institute Inc., 2020, Cary, NC, USA).

## Results

We recruited 80 paramedics from the Emergency Medical Services Vienna for this study. To compare the difference between task-based package-organization (TPO) and traditional emergency kit packing strategies, each participant completed four tasks, using a different emergency kit for each task. Two of these kits were TPO kits, while the other two were organized using a traditional ABC-approach. Primary endpoint was the head-to-head comparison of a novel TPO kit and a novel non-TPO kit.

The modal age category was 18 to 30 years, and participants were predominantly male (78 particpiants, 98%) which reflects the demographics of the ambulance service (mean age 36 years, 92% male; n = 668). Participants had worked in emergency medical services for a median (IQR) of 9 years (5–15 years), and with their current service for 4 years (2–9 years). All participants regularly worked with ambulances (77 participants, 96%), emergency physician response units (7 participants, 9%), field supervisor units (1 participant, 1%), or combinations of these units, and thus regularly use emergency kits in out-of-hospital emergencies. Some participants also staffed additional units such as the prehospital research unit (2 participants), the special mission unit for mass casualty events (2 participants), interhospital transfer unit (2 participants), and the technical rope rescue unit (1 participant). All but 9 participants (89%) were qualified paramedics, while 5 participants (6%) were qualified EMTs. 4 participants did not disclose their qualifications.

**Fig. 2:**
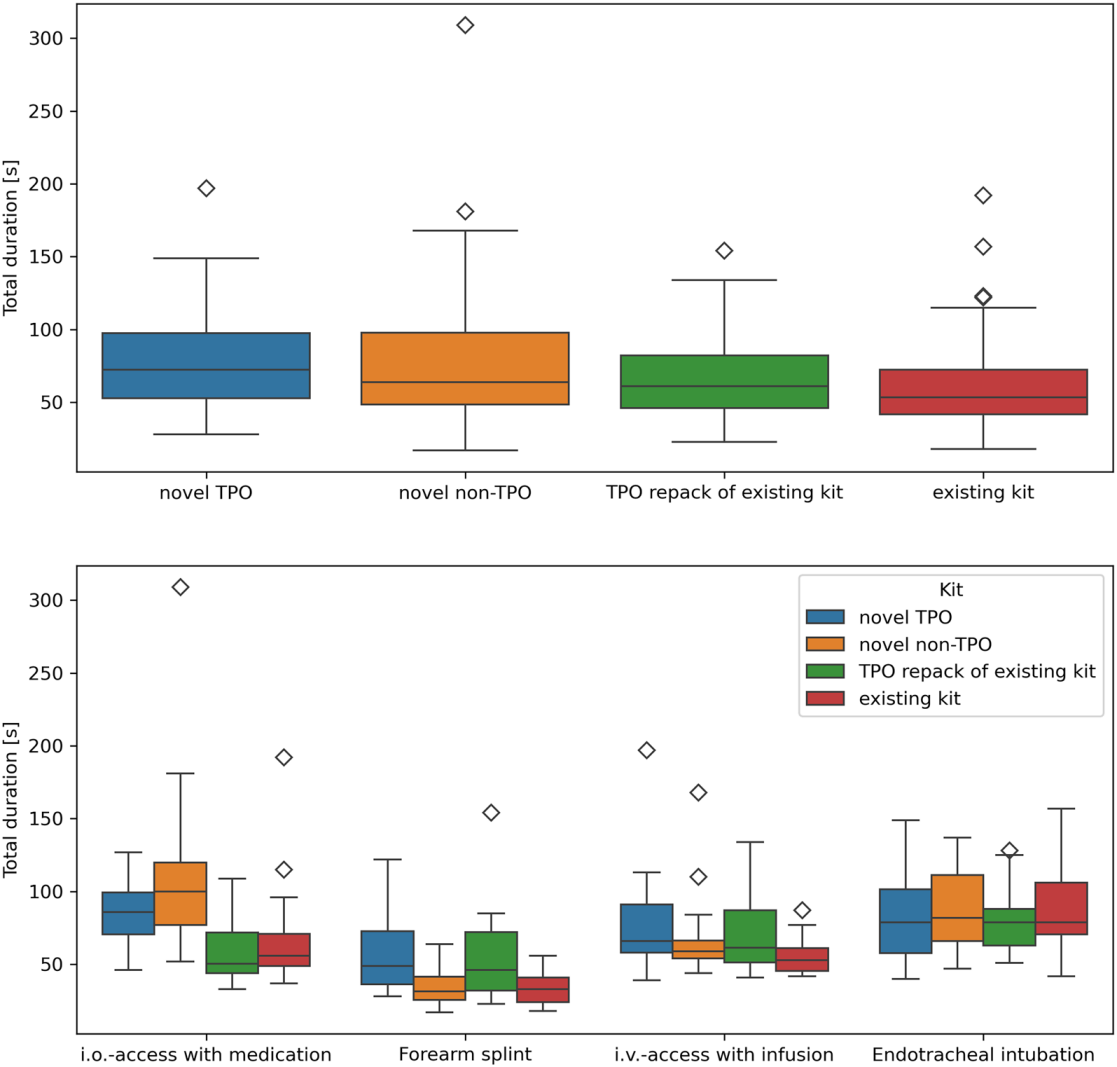
Timing data grouped by kit and task type. Overall, there was a significant between-kit difference over all tasks. However, there was no difference between the novel TPO and novel non-TPO kit. *TPO* task-based package organization, *i.o.* intraossesous, *i.v.* intravenous

Timing data is depicted in Fig. 2. Total time to completion across all tasks was not significantly different between the two novel kits, which was the primary endpoint of the present study (novel TPO vs novel non-TPO, 73 s (53–98 s) vs 64 s (48–99 s), p = 0.11). However, overall, total time to completion was dependent on both the task (p < 0.0001) and the kit type (p < 0.0001). Additional exploratory analysis revealed a significant interaction between the task and the kit type (p < 0.0001). Time to completion was significantly longer when using the novel TPO system to prepare for the forearm splint (49 s (36–75 s)) than the novel non-TPO system (32 s (26–43 s), p < 0.001). The time to completion using these two novel emergency kits for the other three tasks were comparable.

Preparation for endotracheal intubation took longest (81 s (66–103 s)), followed by intraosseous access with medication (75 s (52–96 s)), intravenous access with infusion (60 s (51–76 s)), and forearm splint (38 s (30–54 s)).

The emergency kit currently in service produced the fastest overall times (total time to completion 54 s (42–73 s)), followed by its TPO repack (61 s (46–83 s)). As to be expected, use of the newly acquired systems, with which none of the participant were familiar at the time of the study, proved to be slower (novel non-TPO: 64 s (48–99 s); novel TPO 73 s (53–98 s)).

Seventy-five (94%) participants missed at least one item while preparing for endotracheal intubation; 66 participants missed at least one item during access with infusion; 48 during access with medication; and 46 with the forearm splint. All participants missed at least one item in at least one task. Items which were omitted during initial retrieval are listed in Table 1. The percentage of missing items was distributed similarly across all investigated kits (17% TPO repack, 17% current kit, 18% novel non-TPO, 18% novel TPO) but differed according to the task (range of 11–29%, data provided in the supplementary material).

**Table 1:** Missing items grouped by task type

	# missing
*Endotracheal intubation*	
Plan B kit	66
Stethoscope	54
Extension tubing	24
Cuff syringe	6
ETT fixation material	2
Endotrachealtube w/stylet	1
*i.v.-access with infusion*	
Sharps container	55
Dry gauze (≥ 1 pcs)	13
3-way stopcock	11
Flush syringe	9
i.v. dressing	8
Tourniquet	7
Alcoholic swab	3
i.v. catheter	3
Cristalloid bag	3
i.v. line	3
*i.o.-access with medication*	
Blunt needle	36
3-way stopcock	13
20 mL syringe	10
Flush syringe	7
Ampulle	5
i.o. Drill	2
i.o. needle kit (with needle, sharps container, stabilizer, adapter)	2
*Forearm splint*	
Scissors	32
Triangular bandage	27
Foamed aluminum splint	3
Elastic bandage	3

Checklists for each task are available in the supplementary material

*ETT* Endotracheal tube, *i.v.* intravenous, *i.o.* intraossesous, *mL* millilitres

All but four participants retrieved more items than required for at least one task. More than half of all participants retrieved unnecessary lubricant and half retrieved uncessary BVM equipment during preparations for endotracheal intubation, while more than half retrieved an unnecessary alcoholic swab while preparing for intraosseous access with medication. The exact count of surplus items is shown in Table 2.

**Table 2:** Items retrieved that were not part of the task checklist

Task	Equipment	Count
Endotracheal intubation	Lubricant	56
	BVM Equipment	40
	Magill forceps	36
	Airway adjuncts	11
	Cuff pressure gauge	7
	Conventional laryngoscope	4
	PEEP valve	2
	Additional securing material	2
	Bite block	1
	Additional Syringe	1
	Additional SGA device	1
	Scissors	1
	Additional ET tube	1
Forearm splint	Cool Pack	9
	Adhesive tape	3
	Additional elastic bandage	2
	Additional scissors	1
i.o.-access with medication	Alcoholic swab	43
	Sharps container	26
	Infusion	9
	Dry gauze	8
	Tourniquet	7
	i.v.-line	7
	i.v.-dressing	5
	i.v.-catheter	5
	Syringe cap	4
	Hypodermic Needle	3
	Video Laryngoscope with single-use blade	2
	Additional flush syringe	2
	Additional syringe	2
	Endotracheal tube	1
	Different medication	1
	Additional blunt needle	1
	Additional i.o. needle kit	1
i.v.-access with infusion	Syringe cap	14
	Elastic bandage	2
	i.o. Drill	2
	i.o. needle kit	2
	Blunt needle	1
	Additional i.v.-catheter	1
	Additional flush syringe	1
	Syringe	1

*BVM* Bag-Valve-Mask, *SGA* Supraglottic Airway, *i.o.* intraossesous, *i.v.* intravenous

**Fig. 3:**
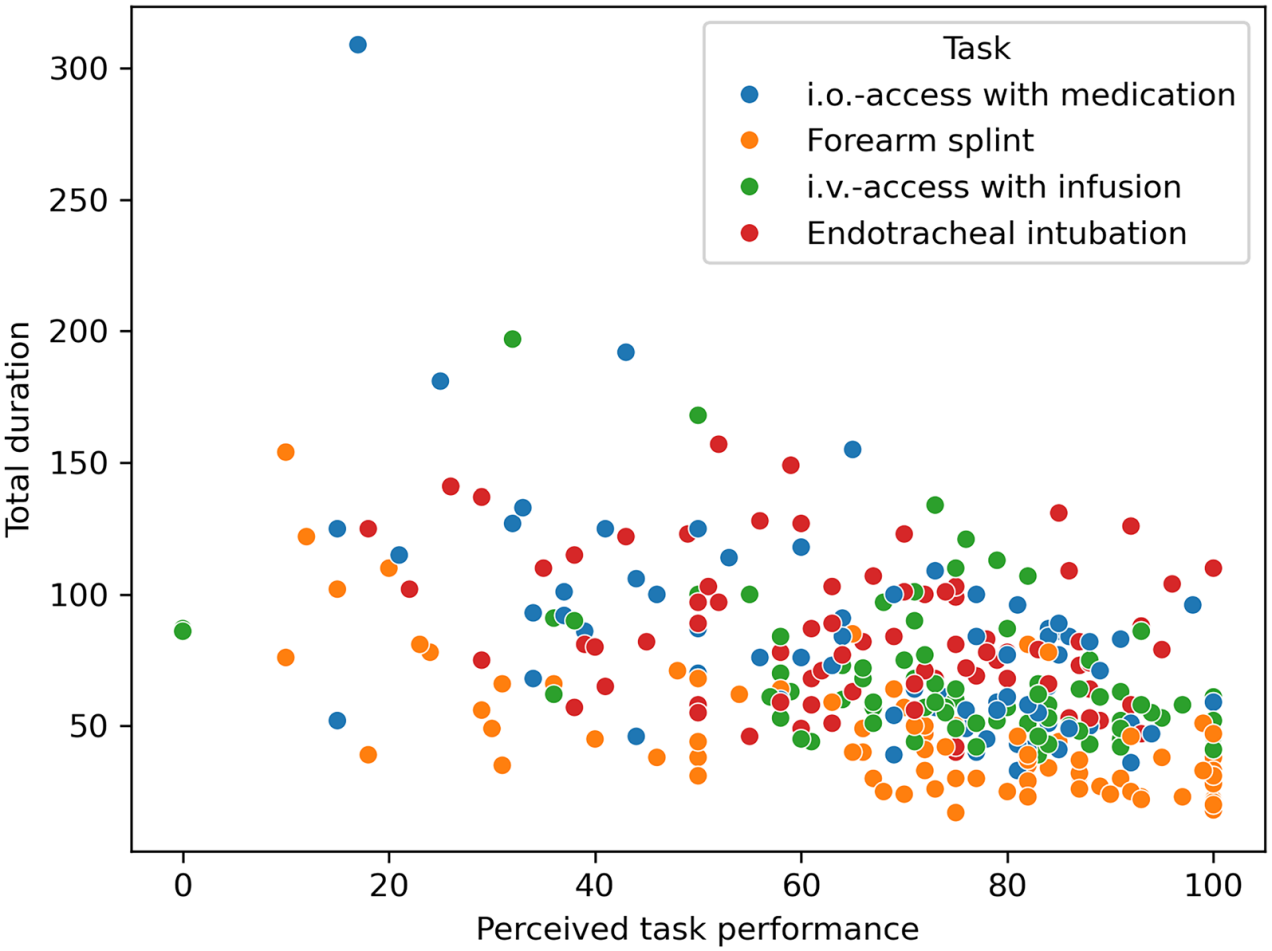
Total duration and perceived task performance correlate (r = −.46). *i.o.*: intraosseous, *i.v.*: intravenous

For exploratory analysis, participants were asked to rate their confidence and perceived performance for each task between zero (not at all confident/very poor performance) and 100 (extremely confident/excellent performance). They were additionally asked to rate the emergency kit. The median perceived performance was 74 (58–85), with similar outcomes for confidence at performing the task (80 (66–94)) and for perceived performance of the emergency kit (74 (51–89)). Confidence in performing the task and perceived performance are moderately well correlated (r = 0.80), while confidence and kit rating correlated less well (r = 0.50). Pairwise plots of these variables are shown in the supplementary materials. Additionally, perceived performance and total duration to task completion also correlate (r = −0.46). The correlation graph can be seen in Fig. 3.

## Discussion

To date, the optimal packing strategy for emergency kits has not been established. While some literature exists on improved packing of in-hospital emergency kits [7, 16], the urban prehospital setting has not yet been investigated. The present study investigated 80 simulated experiments, each with four tasks and four emergency kits. For our primary endpoint in comparing a novel TPO kit and novel non-TPO kit we did not observe a difference in total equipment retrieval time between these kits.

The participants in our study were predominantly young males who regularly worked with emergency kits. This setting is vastly different to previous studies conducted in clinical settings in which most participants were female and with low exposure to emergency kits [7]. Sex differences in handling of emergency equipment have not been investigated to date.

Optimal packing strategies for emergency kits have yet to be established and are likely to depend on several factors. Firstly, some emergency kits may serve a very specific purpose and can therefore be designed under more restrictive assumptions. Schyma et al. optimized an airway bag for in-hospital remote site resuscitation, achieving a 29% decrease in preparation time and a 87.5% reduction in errors; 68.3% of the study participants also perceived the task as being less difficult [16]. In the prehospital emergency setting, however, a single kit needs to serve a wide variety of purposes, ranging from basic diagnostics to potentially highly invasive procedures.

Secondly, the intended use setting plays an important role. Most existing research focuses on the design of in-hospital crash carts [8, 12, 17]. Villamaria et al. have measured the times taken by teams in responding to cardiac arrests, and found that crash carts arrive a median 68 s after the cardiac arrest team has been activated [17]. Crash carts were less readily available in non-patient areas, and it took longer for the crash cart to arrive at the scene of the cardiac arrest. Consequently, even in-hospital teams frequently use smaller, more portable emergency kits (e.g., backpack) for remote emergency response and during patient transport [7, 11]. While some research into the content and organization structure of in-hospital emergency kits has been published [8, 12, 18], prehospital and clinical settings differ significantly. Prehospital emergency services usually work in smaller teams and tighter spaces, and handle a more diverse set of emergencies. The present study was conducted in an urban prehospital EMS setting with a highly modular structure and a variety of units providing different expertise and equipment. Most studies investigating the contents and—in part—packing strategies for emergency kits operated in different settings, such as mountain rescue [2], in-flight emergencies [3], and austere environments [2]. These settings constrain the design of emergency kits so that the results of these studies are of limited value for other settings.

Thirdly, familiarization with the emergency kits is likely to affect performance. Uniquely, TPO has been proposed to offer a benefit for emergency kits which are used only on rare occasions [7] In our data, however, equipment retrieval times did not vary significantly between the two unfamiliar emergency kits (novel TPO versus novel non-TPO), regardless of their organization. Our experiments thus contradict existing literature on TPO. Data on the performance of TPO versus non-TPO emergency kits after adequate equipment familiarization is lacking.

When preparing equipment for a procedure, forgetting items is a common problem and is comprehensively documented in the literature [7, 16]. Cognitive aids have therefore been recommended for complex procedures with significant risk [19, 20]. In recent years, mnemonics, checklists [21], prepacked kits [16], and dedicated staging area backgrounds (kit dump sheets) [21] have been described. Sommer et al. [7] have described TPO as a novel cognitive aid strategy for use in unexpected emergency settings in which crash carts are infeasible and where other cognitive aids might not be readily available. They were able to not only demonstrate a significant reduction in missing items across all tasks (neonatal endotracheal intubation, neonatal intravenous access, neonatal intraosseous access), but also a significant reduction in retrieval times. Despite these promising results, our experiments did not reproduce these results: using TPO kits did not result in more complete retrieval compared to traditional kits, and retrieval time was comparable across all emergency kits. Exploratory analysis did, however, reveal quicker retrieval times when using the novel TPO system compared to the novel non-TPO system to prepare for a forearm splint. Assuming that in real-life settings different tasks are assigned different priorities (based on urgency and expected task frequency), some backpack designs may perform differently than in our simulated setting which prioritized each task equally. These priorities should be considered when designing an emergency kit because it may be feasible to optimize kits under these constraints and a TPO strategy could prove beneficial.

Despite the precise task assignments, surplus items were retrieved for every task. The most common surplus items were a lubricant gel for endotracheal intubation and an alcoholic swab for intraosseous access. Both of these items were removed from initial versions of the checklists after being frequently missed in the piloting phase. Only very few participants misunderstood the initial assignment and retrieved items for similar but different tasks (e.g., retrieval of an intraosseous drill while preparing for intravenous access). The wide range of the surplus items suggests a high inter-provider-variability in clinical practice from experienced healthcare providers.

Successfully employing TPO will always require that equipment required for multiple tasks is stored in multiple locations, as can be seen from the packaging table provided by Sommer et al. [7]. This redundancy is likely to lead to increased weight of emergency kits, representing an added physical burden for already strained emergency medical services workers [22]. Moreover, some of the assumptions and perhaps even personal experiences of the kit designers themselves are manifested in the organization of the TPO modules: as demonstrated by commonly omitted and surplus items, providers may have different definitions of the equipment a task entails. We hypothesize that highly standardized procedure definitions and processes could circumvent this complication, as is the case for airway management in many emergency medical services, especially those with a high rate of invasive procedures [23, 24].

### Strengths and limitations

Our study has several strengths. Highly standardized conditions allowed the different emergency kits to be objectively assessed. Careful randomization and blind analysis reduced relevant biases. A large sample size supports the outcome of statistical tests.

Despite these strengths, relevant study limitations need to be acknowledged. Firstly, while simulations are an important tool for safely evaluating new equipment or procedures, they cannot completely mirror their real-life use. However, this study design was selected to maximize consistency between experiments. Secondly, participants had not previously worked with the emergency kits provided, and the participants were not examined after the initial learning phase with the respective kits. This limits generalizeability of our results to every-day use of TPO but adds important data to the previously established hypotheses that TPO might benefit users with limited exposure to these emergency kits. Finally, task-oriented package organization is not strictly defined, and TPO may be variously interpreted by the different organizations and providers.

## Conclusion

The present study is the first to investigate task-based package organization in an urban emergency medical service setting, following relevant research on emergency kit packing strategies conducted in various in- and out-of-hospital settings. Our experiments did not reveal a difference between novel TPO and non-TPO emergency kits in equipment retrieval times and completeness across all tasks, but do suggest that optimizing emergency kits for specific high-priority tasks may be beneficial. Further research is warranted to better and consistently characterize TPO and to optimize prehospital equipment organization according to the special settings in which EMS teams work.

## Supplementary Information

Additional file1 (DOCX 501 kb)

Additional file2 (PDF 2785 kb)

Additional file3 (PDF 2841 kb)

Additional file4 (PDF 2160 kb)

Additional file5 (PDF 2314 kb)

## Data Availability

The datasets used during and/or analysed during the current study are available from the corresponding author on reasonable request.
