# Supplementary material for: Conventional versus task-based package organization for out-of-hospital emergency kits: an emergency medical services simulation study
Source: Scand J Trauma Resusc Emerg Med. 2024 Dec 20;32:135. doi: 10.1186/s13049-024-01309-8 (PMC11660938; doi:10.1186/s13049-024-01309-8)
Supplement: Supplementary file 1 — Additional file1 (DOCX 501 kb) [file 13049_2024_1309_MOESM1_ESM.docx]

Supplement

[Questionnaires 2](#_Toc181197744)

[Initial Questionnaire 2](#_Toc181197745)

[Task Questionnaire 3](#_Toc181197746)

[Final Questionnaire 3](#_Toc181197747)

[Briefing Texts 4](#_Toc181197748)

[Tasks 5](#_Toc181197749)

[Example-Task (briefing only, not a real task) 5](#_Toc181197750)

[Task: “Preparation for endotracheal intubation” 5](#_Toc181197751)

[Task: “Preparing an intravenous access and crystalloid infusion” 5](#_Toc181197752)

[Task: “Preparing intraosseous access with medication” 5](#_Toc181197753)

[Task: “Preparing for a forearm splint” 5](#_Toc181197754)

[Task Checklists 7](#_Toc181197755)

[Percentage of missing items by kit and task 9](#_Toc181197756)

[Perceived Performance, Confidence, Kit handling 9](#_Toc181197757)

# Questionnaires

All questionnaires were German but translated to English for the purpose of publication. Original questionnaires are available upon request from the corresponding author.

## Initial Questionnaire

- Age (single choice)
  - 18-30 years old
  - 31-40 years old
  - 41-50 years old
  - 51-60 years old
  - 61-70 years old
- Sex (single choice)
  - Male
  - Female
  - Other
- Highest level of Education (single choice)
  - National Qualification Register Level 4 / matriculation qualification
  - National Qualification Register Level 5 / vocational matriculation qualification
  - National Qualification Register Level 6 / Bachelors degree or comparable qualification
  - National Qualification Register Level 7 / Masters degree or comparable qualification
  - National Qualification Register Level 8 / Doctorate
  - Others — please specify
- Highest Medical Qualification (single choice)
  - Emergency Medical Technician
  - Paramedic
  - Paramedic with general qualification to administer medication
  - Paramedic with general qualification to punctuate veins
  - Paramedic with special qualification for endotracheal intubation and ventilation
- Total number of years in EMS
- Total number of years at the current EMS service (Emergency Medical Service Vienna)
- Current EMS Units (multiple choice)
  - Ambulance Car
  - Emergency Physician Response Vehicle
  - Field Supervisor Response Vehicle
  - Supervising Physician Response Vehicle
  - Research Response Vehicle
  - Special Operations Response Vehicle
  - Others — please specify
- I am _____ in using the current emergency kit. (scale 0 to 100, where 0 is not confident and 100 is very confident)
- I would rate the current emergency kit as _____. (scale 0 to 100, where 0 is very bad and 100 is very good).

## Task Questionnaire

- Performing this task I felt _____. (scale 0 to 100, where 0 is not confident and 100 is very confident)
- I think my performance on this task was _____. (scale 0 to 100, where 0 is very bad and 100 is very good)
- The emergency kit handling was _____. (scale 0 to 100, where 0 is very bad and 100 is very good)

## Final Questionnaire

- I think the emergency kit is _____ in my daily work. (scale 0 to 100, where 0 is not important and 100 is very important)
- I think involving users in the decision making process on future equipment is _____. (scale 0 to 100, where 0 is not important and 100 is very important)
- Please order the four emergency kits you have just used to your preference from favorite to least favorite.
- Please state advantages of the TPO emergency kit.
- Please state disadvantages of the TPO emergency kit.
- Please state advantages of the non-TPO emergency kit.
- Please state disadvantages of the non-TPO emergency kit.
- I experienced the eye-tracking glasses as _____. (scale 0 to 100, where 0 is not irritating and 100 is very irritating)
- How did the eye-tracking glasses irritate you?

# Briefing Texts

This is the translated version. The original German version is available from the corresponding author upon request.

*Welcome to the EMES Study!*

*The goal is to prepare equipment for a number of interventions in as little as time as possible.*

*You must retrieve the required equipment for a given task from the provided emergency kit. Assume you are assisting an emergency physician who does not carry any personal equipment. You only need to retrieve the equipment, but you do not need to assemble anything. You also do not need to open any packaging (e.g. opening the infusion line and priming the line is not necessary). If an intervention requires equipment that is not located in the emergency kit (e.g.: oxygen, capnography-line) you do not need to prepare or retrieve this.*

*We look for both speed and completeness. The time starts once you open the emergency kits and stops once you announce that you have finished retrieving the equipment (“stop” or “done”).*

*If you cannot not find what you are looking for, please let the study personnel know immediately. Once acknowledged, you can omit the equipment in question.*

# Tasks

The translated version of task specifications are shown below. Original versions (German) are available upon request from the corresponding author.

## Example-Task (briefing only, not a real task)

Your task is to prepare Bag-Valve-Mask ventilation with oro- and nasopharyngeal airway adjuncts.

Specific instructions for this task:

- Oxygen does not need to be connected
- The size of the face mask can be chosen freely
- Checking and/or assembling of equipment is **not** part of this task

## Task: “Preparation for endotracheal intubation”

Your task is to prepare the endotracheal intubation using a video-laryngoscope. The patient is already being ventilated.

Specific instructions for this task:

- Checking the endotracheal cuff is **not** required. The size of the endotracheal tube may be chosen freely.
- Assembling/checking the video-laryngoscope is **not** part of this task.
- Checking and/or assembling of equipment is **not** part of this task

## Task: “Preparing an intravenous access and crystalloid infusion”

Your task is to prepare an intravenous access including equipment to flush the i.v. access and a three-way stopcock as well as an crystalloid solution.

Specific instructions for this task:

- One needle size will be enough. The size may be chosen freely.
- Assembling the infusion line (e.g., priming the line) is **not** part of this task.
- Checking and/or assembling of equipment is **not** part of this task

## Task: “Preparing intraosseous access with medication”

You are working a cardiopulminary-resuscitation scenario. You are tasked with preparing for intraosseous access as well as the first dose of epinephrine.

Specific instructions for this task:

- One needle size will be enough. The size may be chosen freely.
- Drawing up the medication into a syringe is **not** part of this task.
- Checking and/or assembling of equipment is **not** part of this task

## Task: “Preparing for a forearm splint”

You are treating a patient with a closed forearm fracture. You are tasked with preparing for the forearm splint as well as limb immobilization.

Specific instructions for this task:

- Applying/forming the splint is **not** part of this task.
- Checking and/or assembling of equipment is **not** part of this task

# Task Checklists

**Task: Preparation for endotracheal intubation**

- Video-Laryngoscope
- Single-use blade
- ET tube with preloaded stylet (size does not matter)
- syringe for blocking
- expandable extension tubing for ventilation.
- material for securing the tube.
- Plan B-kit (Placing the package on the staging area is sufficient. The package does not need to be unpacked.)
- stethoscope

**Task: Preparation for intravenous access and infusion**

- Tourniquet
- ready-to-use alcoholic swab.
- Intravenous catheter
- i.v. catheter dressing
- pre-filled 10ml syringe or combination of 10ml syringe and normal saline
- Dry gauze (≥ 1 piece)
- Sharps container
- 500 ml bag/bottle of a crystalloid solution
- i.v. line
- 3-way-stopcock

**Task: Preparation for intraosseous access and drug application**

- IO drill
- IO Needle Kit (containing needle, stabilizer, sharps container, connector tubing)
- pre-filled 10ml syringe or combination of 10ml syringe and normal saline
- 20 ml ampule of Adrenaline (1 mg/10 ml)
- 1 blunt needle
- 20 ml syringe
- 3-way-stopcock

**Task: Preparation for a forearm splint**

- Foam Aluminum Splint (commonly referred to by the brand name SAM® Splint)
- Elastic Bandage
- Triangular Bandage
- Scissors

# Percentage of missing items by kit and task

| **Kit** | **Task** | **% missing** |
| --- | --- | --- |
| **novel TPO** | **i.o.-access with medication** | 11.3% |
| **TPO repack of existing kit** | **i.o.-access with medication** | 11.4% |
| **existing kit** | **i.v.-access with infusion** | 12.1% |
| **novel TPO** | **i.v.-access with infusion** | 13.3% |
| **existing kit** | **i.o.-access with medication** | 15.0% |
| **TPO repack of existing kit** | **i.v.-access with infusion** | 15.0% |
| **novel non-TPO** | **i.o.-access with medication** | 15.7% |
| **existing kit** | **Forearm splint** | 16.7% |
| **novel non-TPO** | **Entotracheal intubation** | 16.9% |
|  | **i.v.-access with infusion** | 17.0% |
| **TPO repack of existing kit** | **Forearm splint** | 18.4% |
| **novel TPO** | **Forearm splint** | 20.0% |
| **TPO repack of existing kit** | **Entotracheal intubation** | 23.2% |
| **novel non-TPO** | **Forearm splint** | 26.3% |
| **existing kit** | **Entotracheal intubation** | 26.3% |
| **novel TPO** | **Entotracheal intubation** | 29.4% |

# Perceived Performance, Confidence, Kit handling


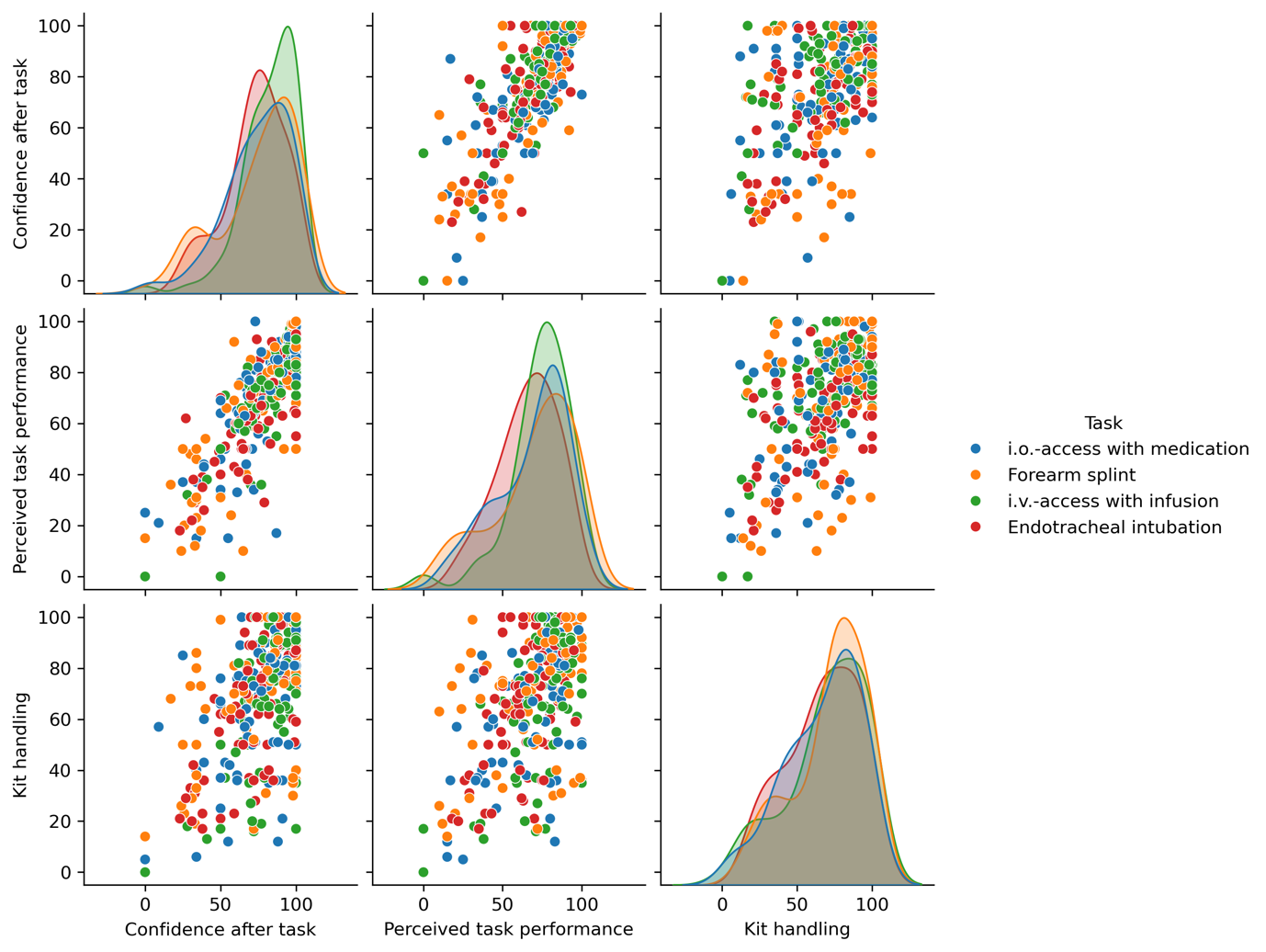


i.o.: intraossesous, i.v.: intravenous
