# Supplementary material for: Conventional versus task-based package organization for out-of-hospital emergency kits: an emergency medical services simulation study
Source: Scand J Trauma Resusc Emerg Med. 2024 Dec 20;32:135. doi: 10.1186/s13049-024-01309-8 (PMC11660938; doi:10.1186/s13049-024-01309-8)
Supplement: Supplementary file 2 — Additional file2 (PDF 2785 kb) [file 13049_2024_1309_MOESM2_ESM.pdf]

Novel kit (TPO)

Airway	video-laryngoscope<br>blades (various sizes)<br>endotracheal tube fixation material<br>extension tubing<br>laryngoscope handle<br>laryngoscope blades (various sizes)<br>magill forceps<br>10ml syringe<br>oropharyngeal Airways (various sizes)<br>nasopharyngeal airways (various sizes)
SGA	i-gel (various sizes)<br>lubricant
Intubation	stylet<br>endotracheal tubes (various sizes)<br>lubricant
Ampullarium (top half)	various medications
Ampullarium (bottom half)<br>compartments left side	 rectal midazolam<br>syringe connector<br>MAD adapters<br>intraosseous drill<br>3-way stopcock<br>syringes (various sizes)<br>intraosseous needle-kits (various sizes)
compartments right side	hypodermic needles<br>spike adapter<br>3-way stopcocks<br>i.v.-catheters (various sizes)<br>tourniquet<br>blunt needles<br>i.v.-dressings<br>syringe caps<br>alcoholic swabs<br>dry gauze<br>flush syringe
Infusion (top compartment)	flush syringes<br>sharps container

Infusion (lower compartment)	paracetamol (100mL)<br>crystalloid infusion<br>glucose infusion<br>methoxyflurane inhaler<br>i.v. lines
Diagnostics	pen light<br>stethoscope<br>single-use clamp<br>ear thermometer<br>blood-pressure cuff<br>glucose measurement set<br>vomit bag<br>permanent marker
BVM	self-inflating bag<br>oxygen tubing<br>oxygen resevoir<br>masks (various sizes)<br>PEEP valve<br>respiratory filter (various sizes)
Trauma (top compartment)	emergency blankets<br>emergency bandage<br>hemostatic dressing<br>tourniquets<br>foamed aluminum splint<br>pelvic binder
Trauma (lower compartment)	antiseptic spray<br>elastic bandage<br>cool packs<br>various wound dressings<br>triangular bandages<br>self-adhesive bandage<br>adhesive tape<br>plasters<br>scissors
Airway (rear)	lubricant<br>oropharyngeal Airways (various sizes)<br>nasopharyngeal airways (various sizes)

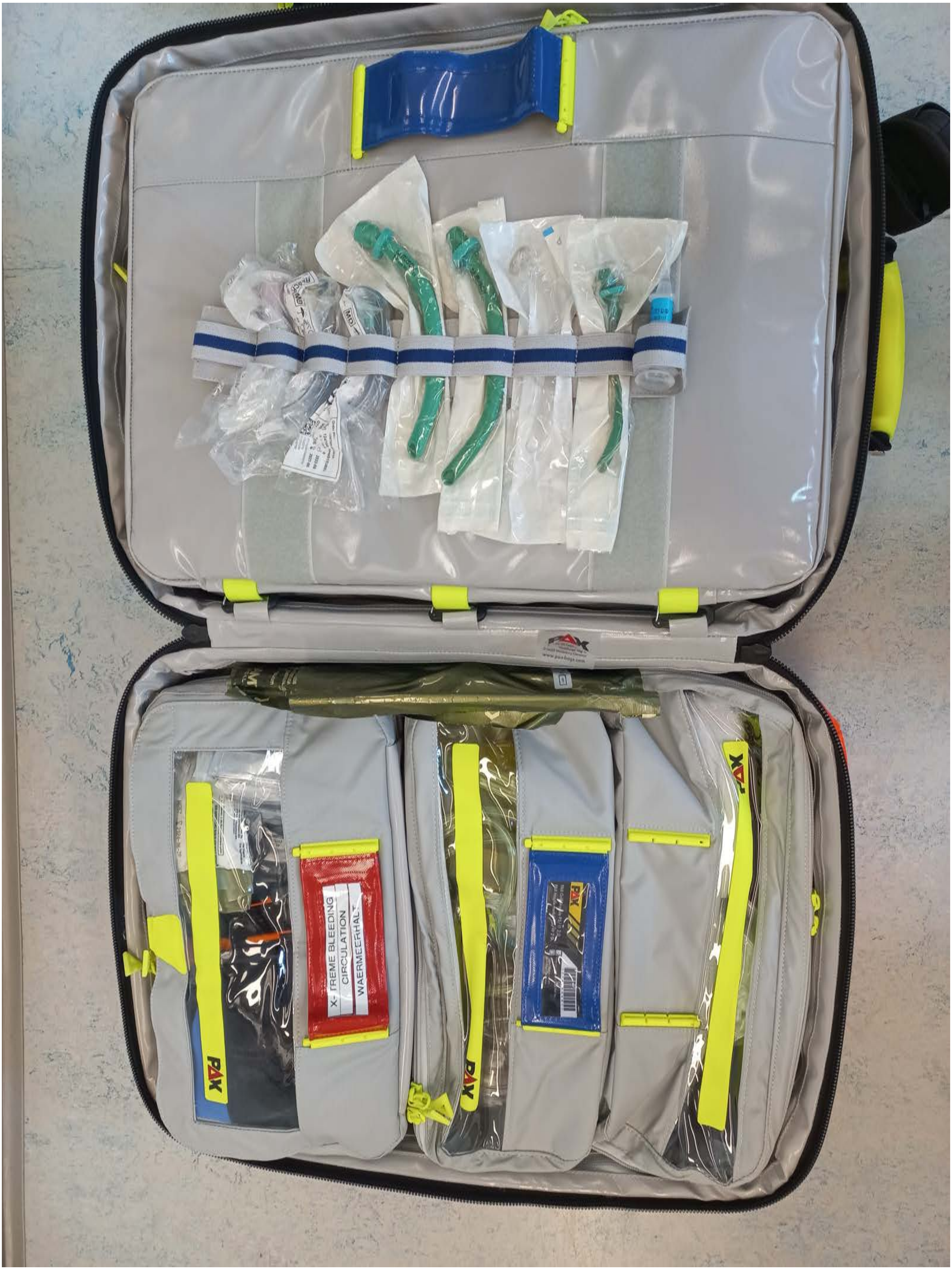
X-TREME BLEEDING
CIRCULATION
WAERMEERHALT

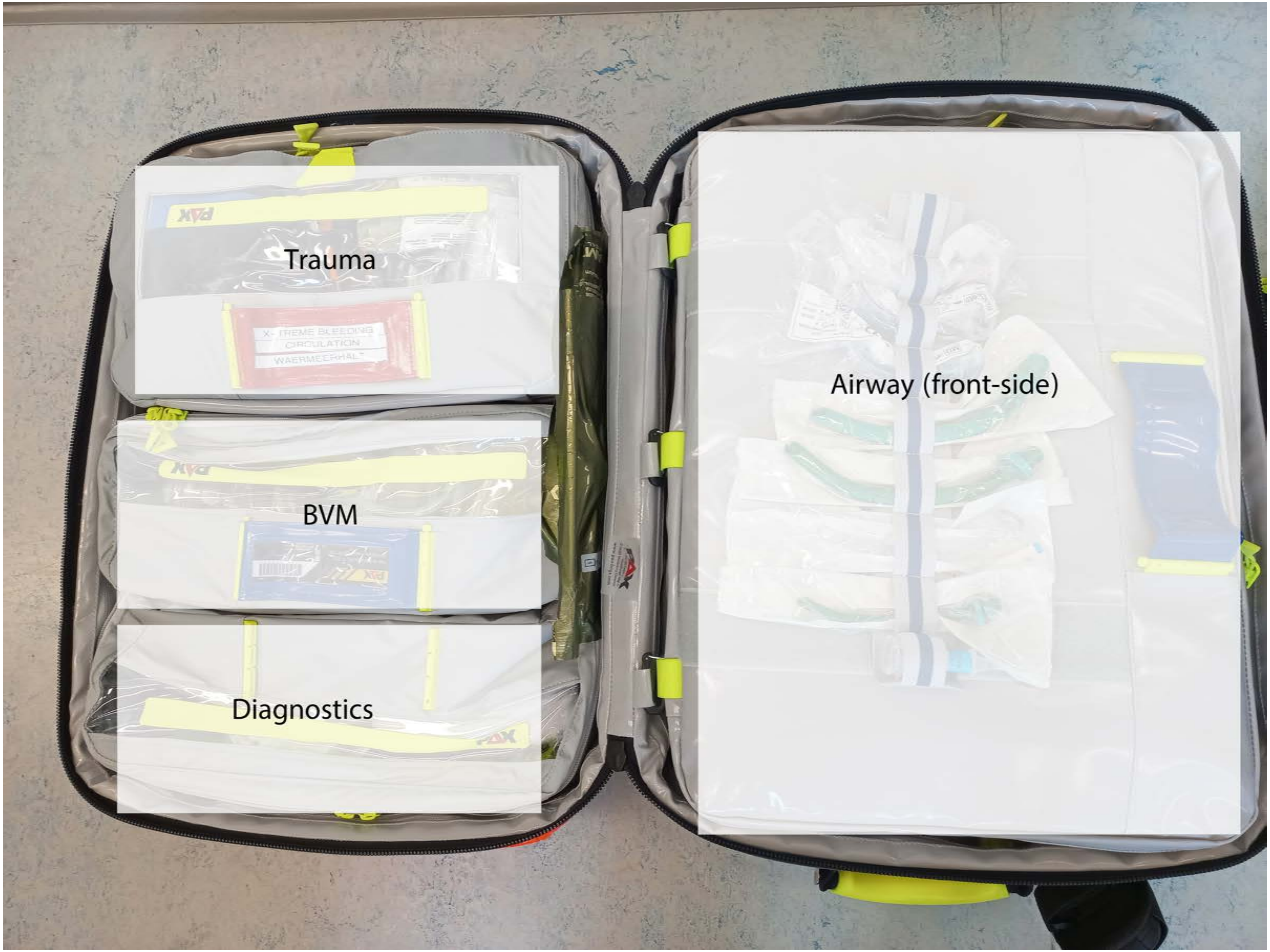
Trauma
X- TREME BLEEDING
CIRCULATION
WAERMEERHALT
BVM
Diagnostics
Airway (front-side)

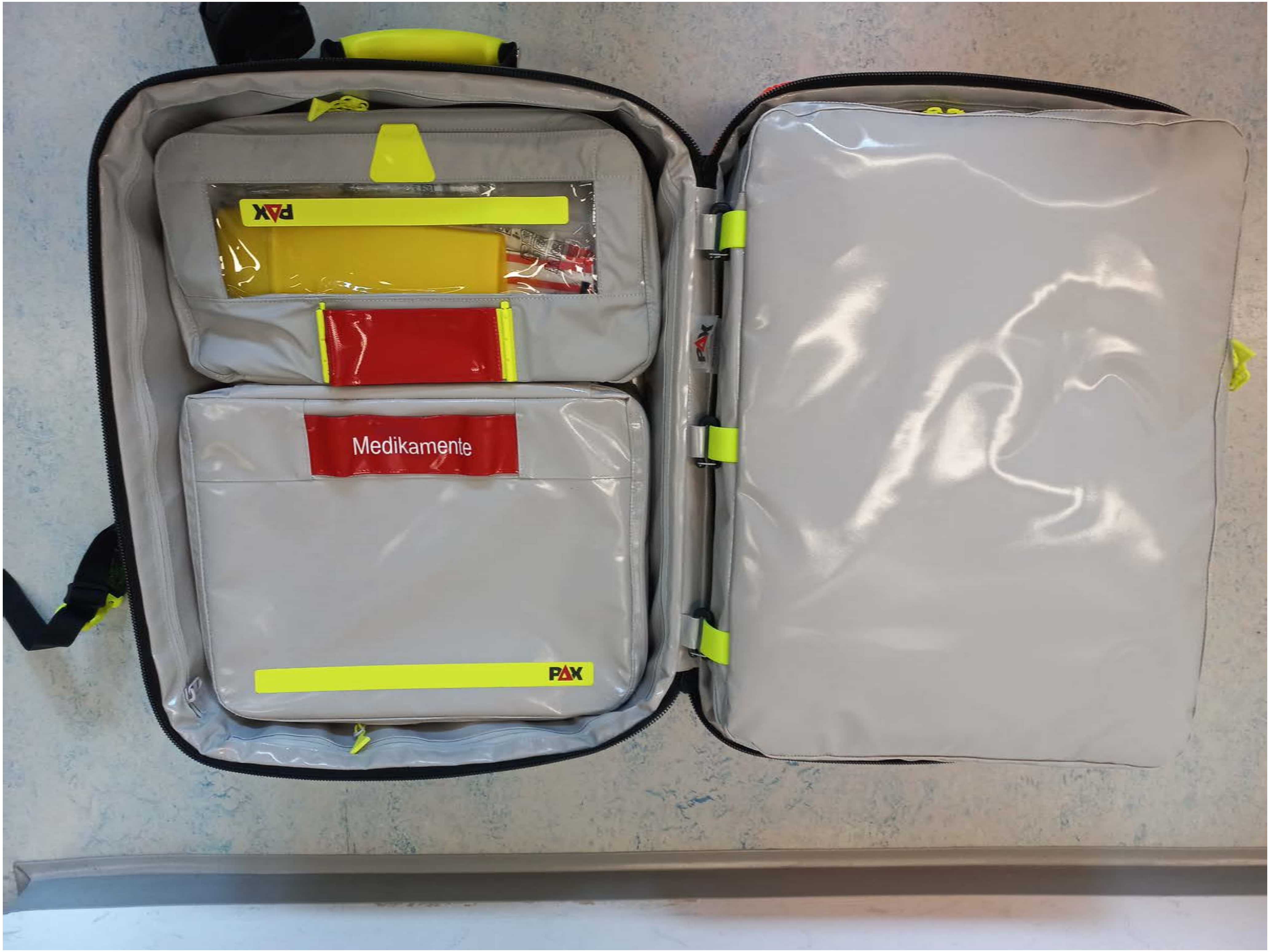
Medikamente
PAX

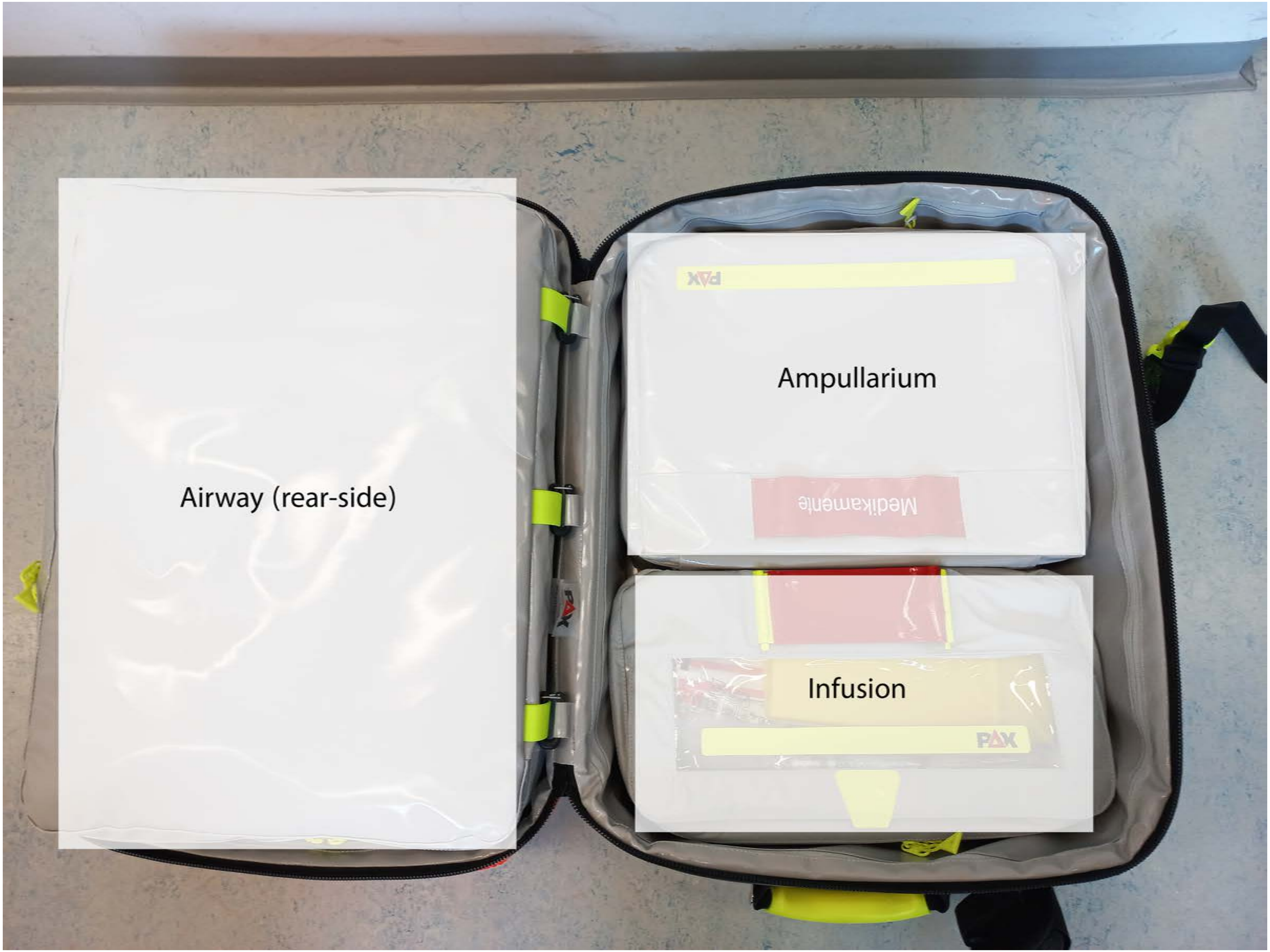

Airway (rear-side)
Ampullarium
Medikamente
Infusion

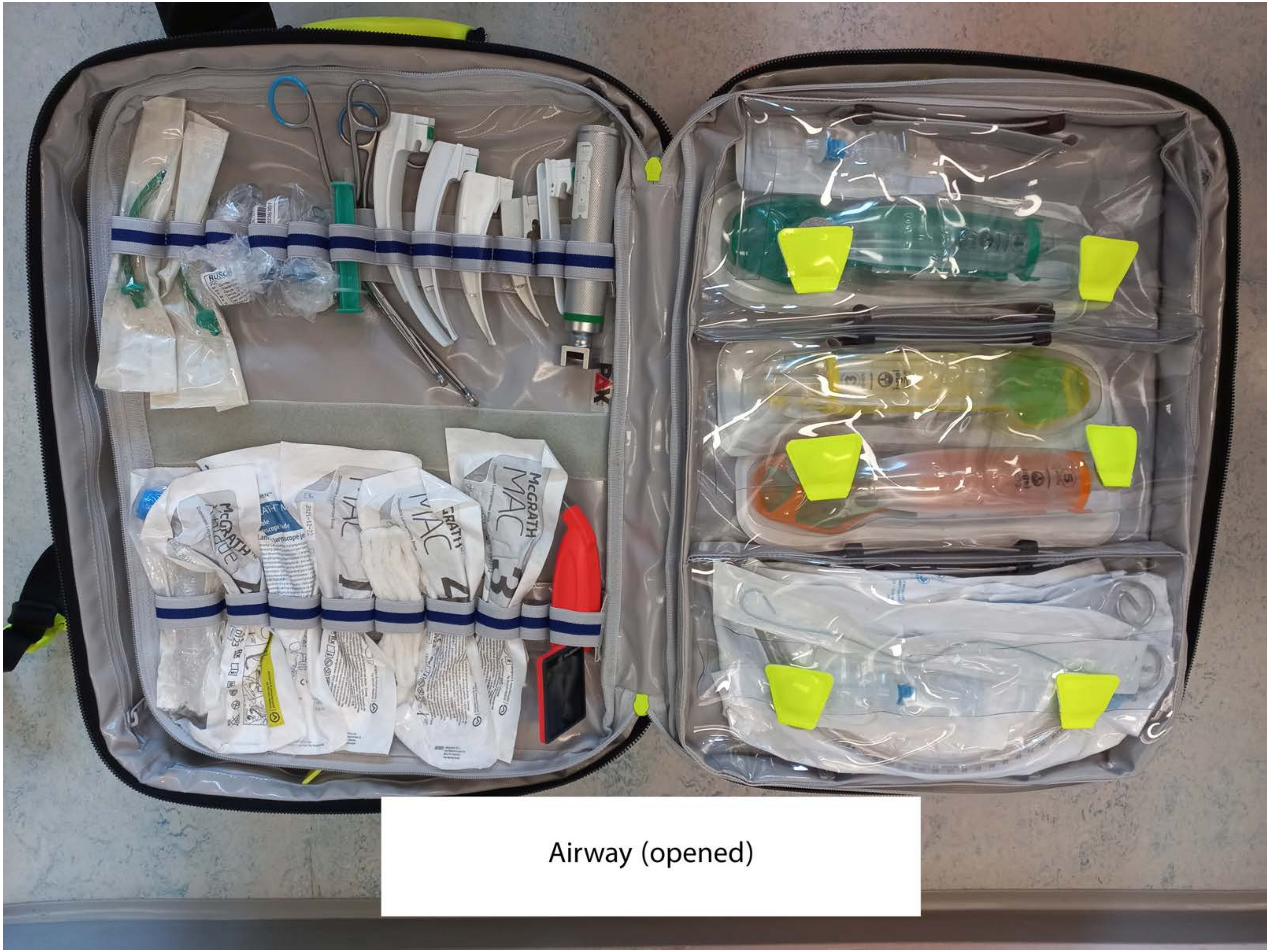

Airway (opened)

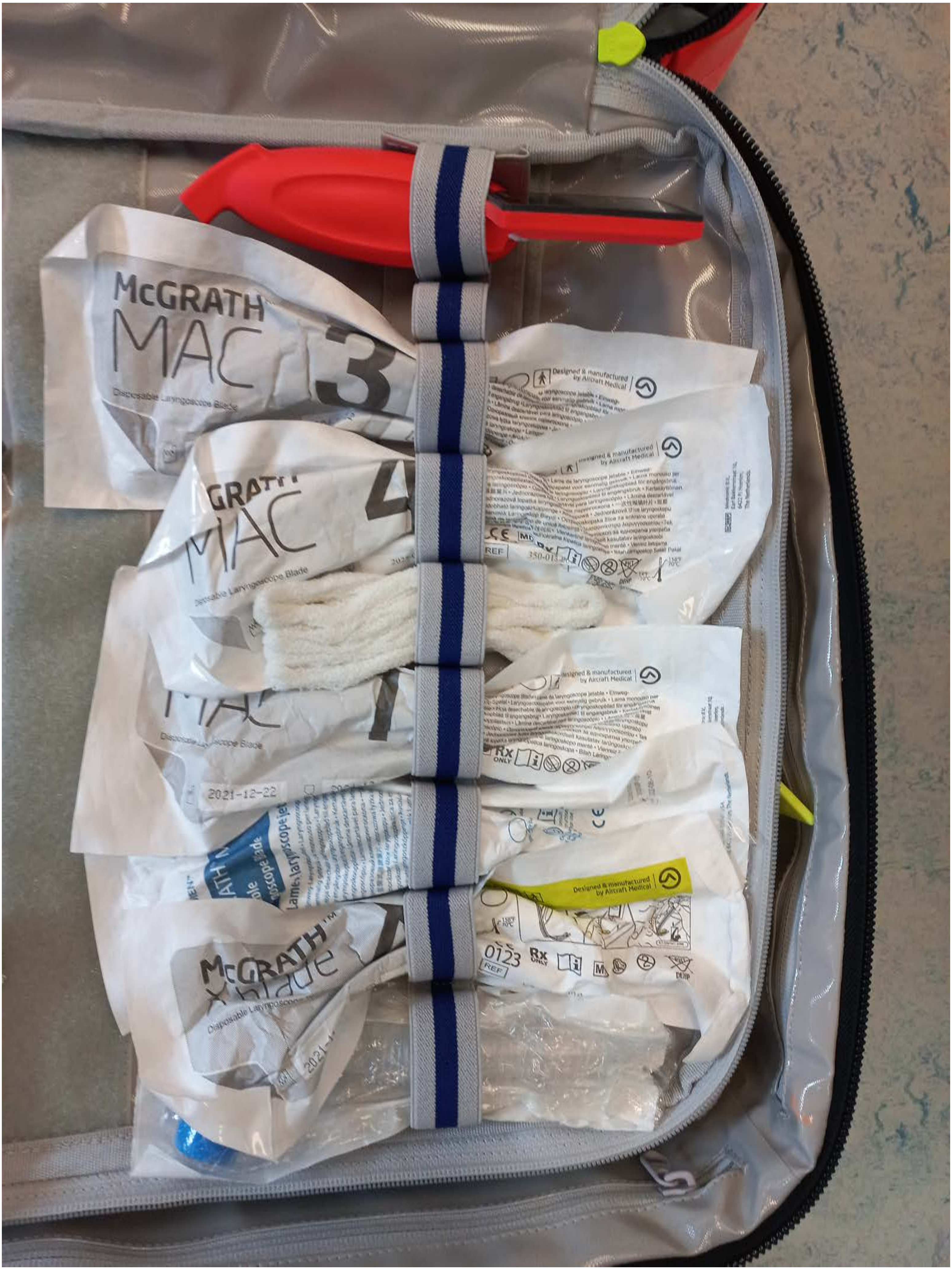
McGRATH MAC 3
Disposable Laryngoscope Blade
Designed & manufactured by Aircraft Medical
2021-12-22
0123
Rx ONLY
REF
McGRATH X blade

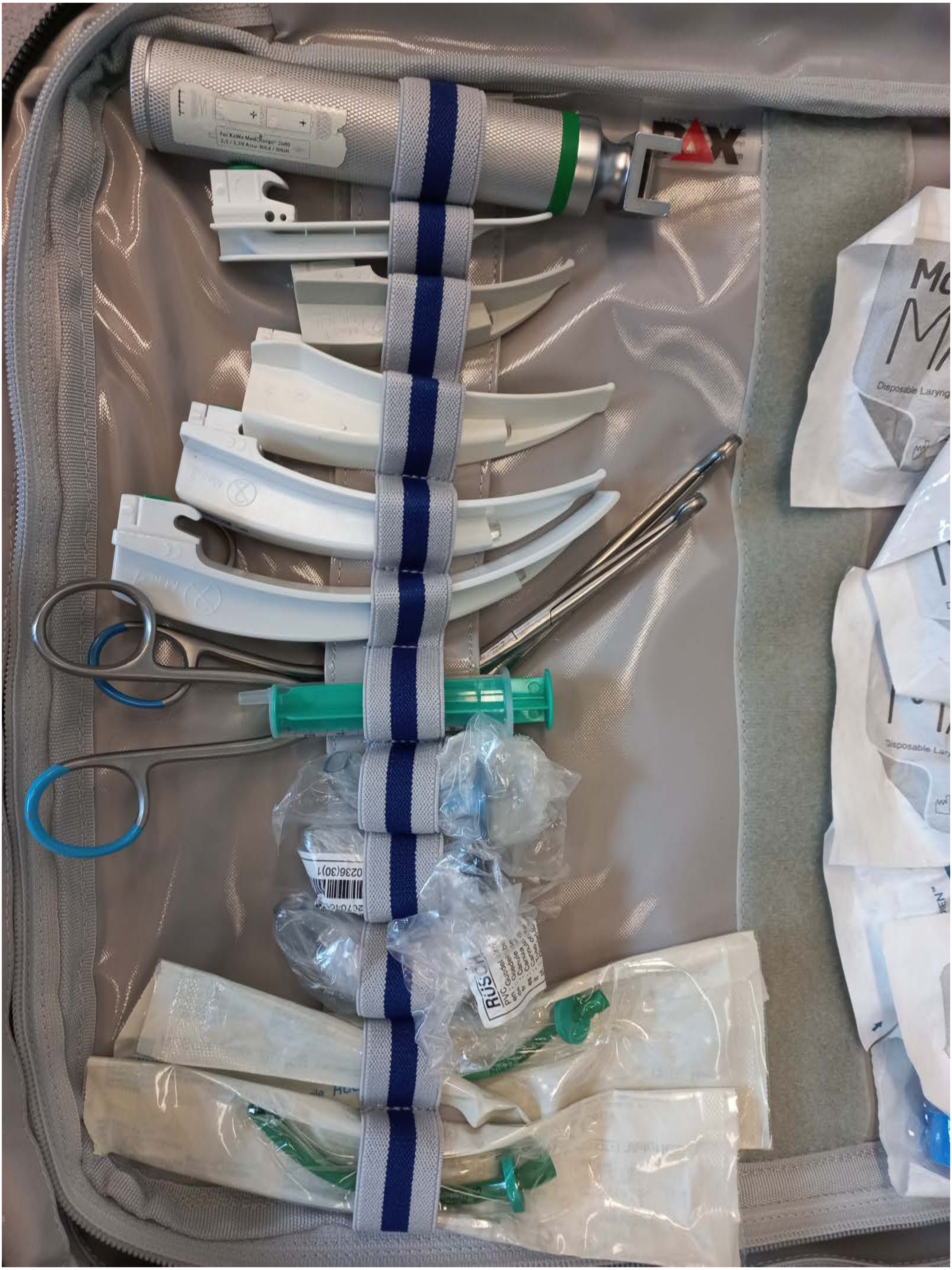

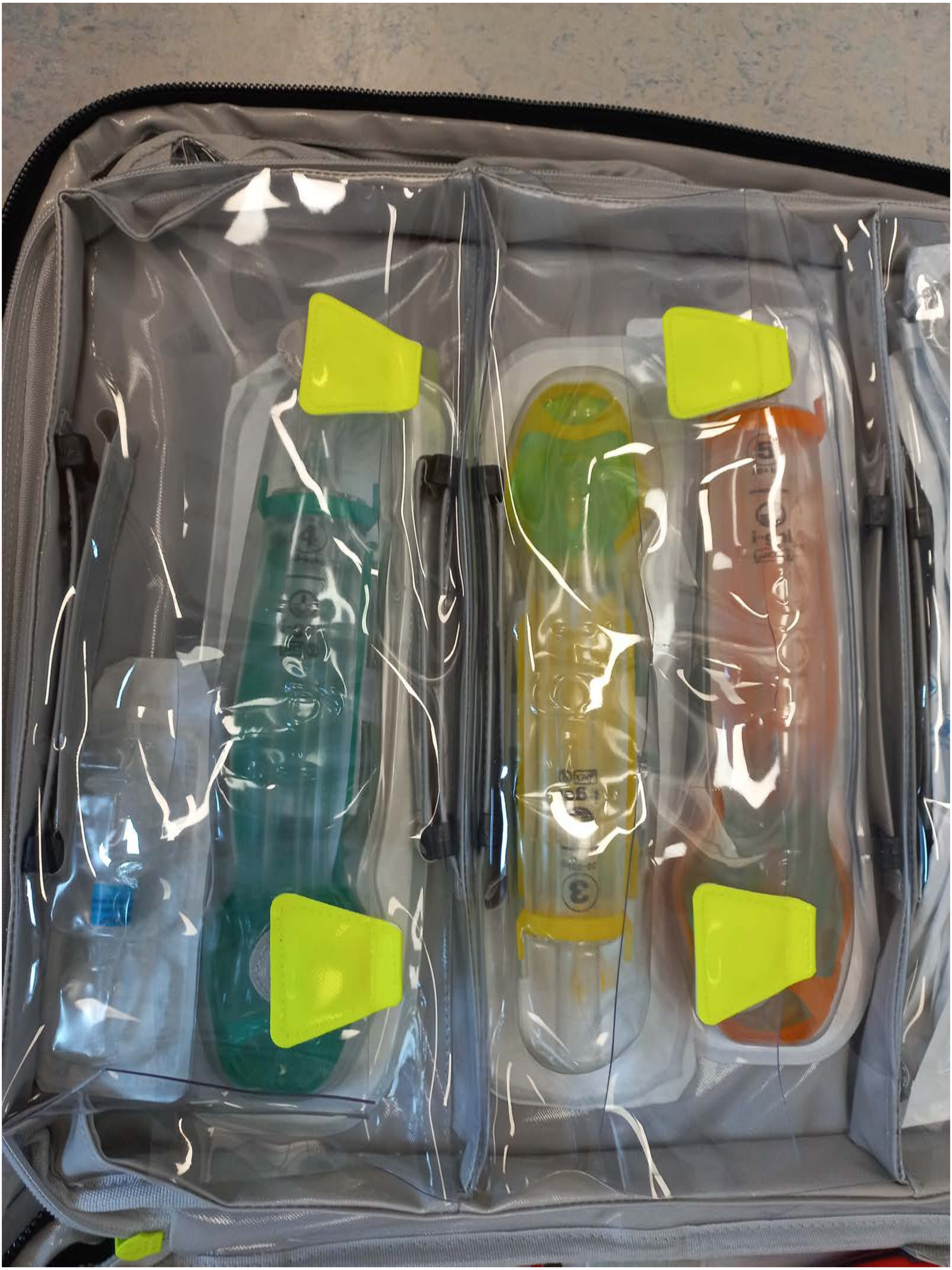

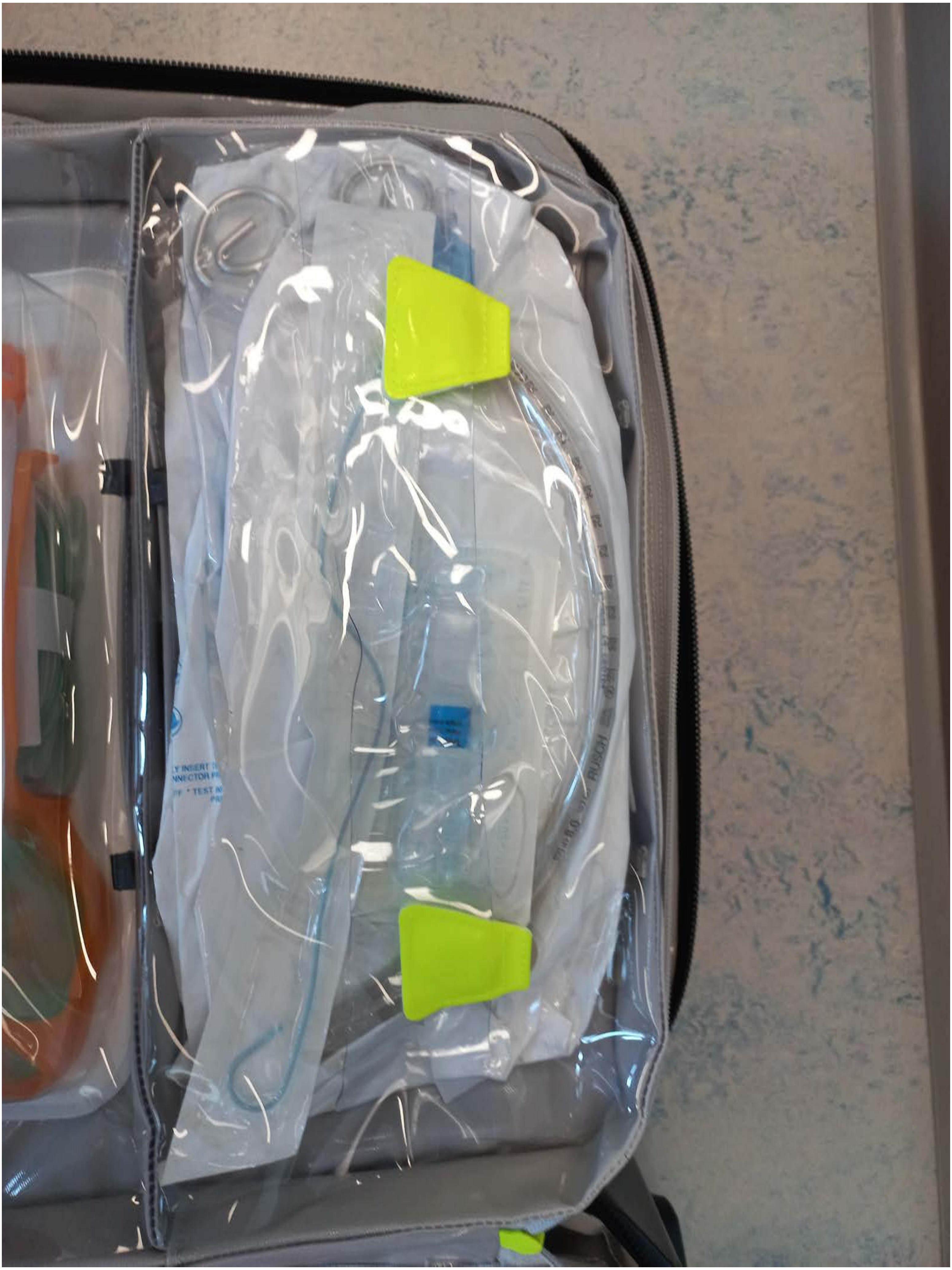
RUSCH

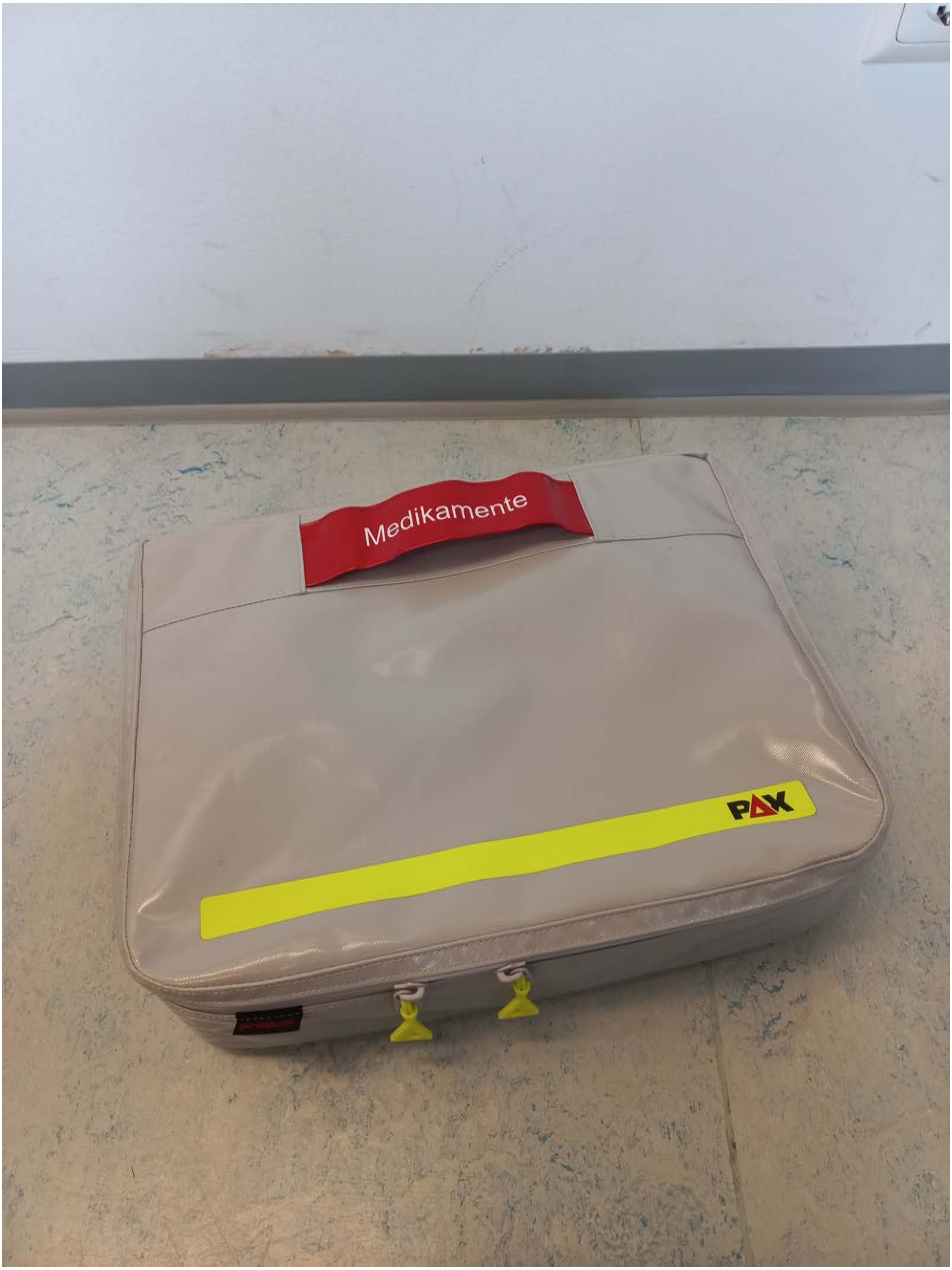

Medikamente
PAX

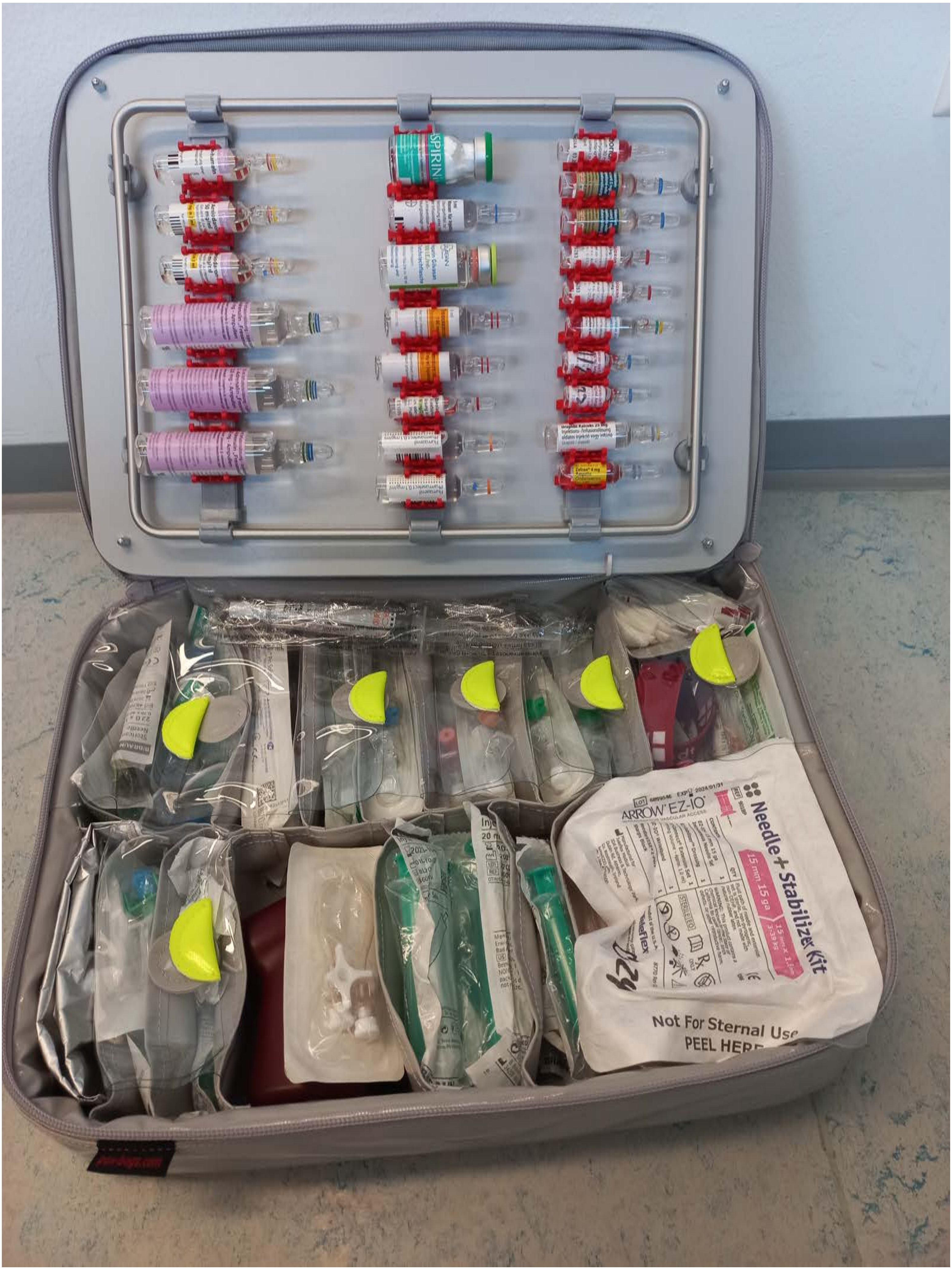
ARROW EZ-IO
Needle + Stabilizer Kit
15 mm 15 ga
Not For Sternal Use

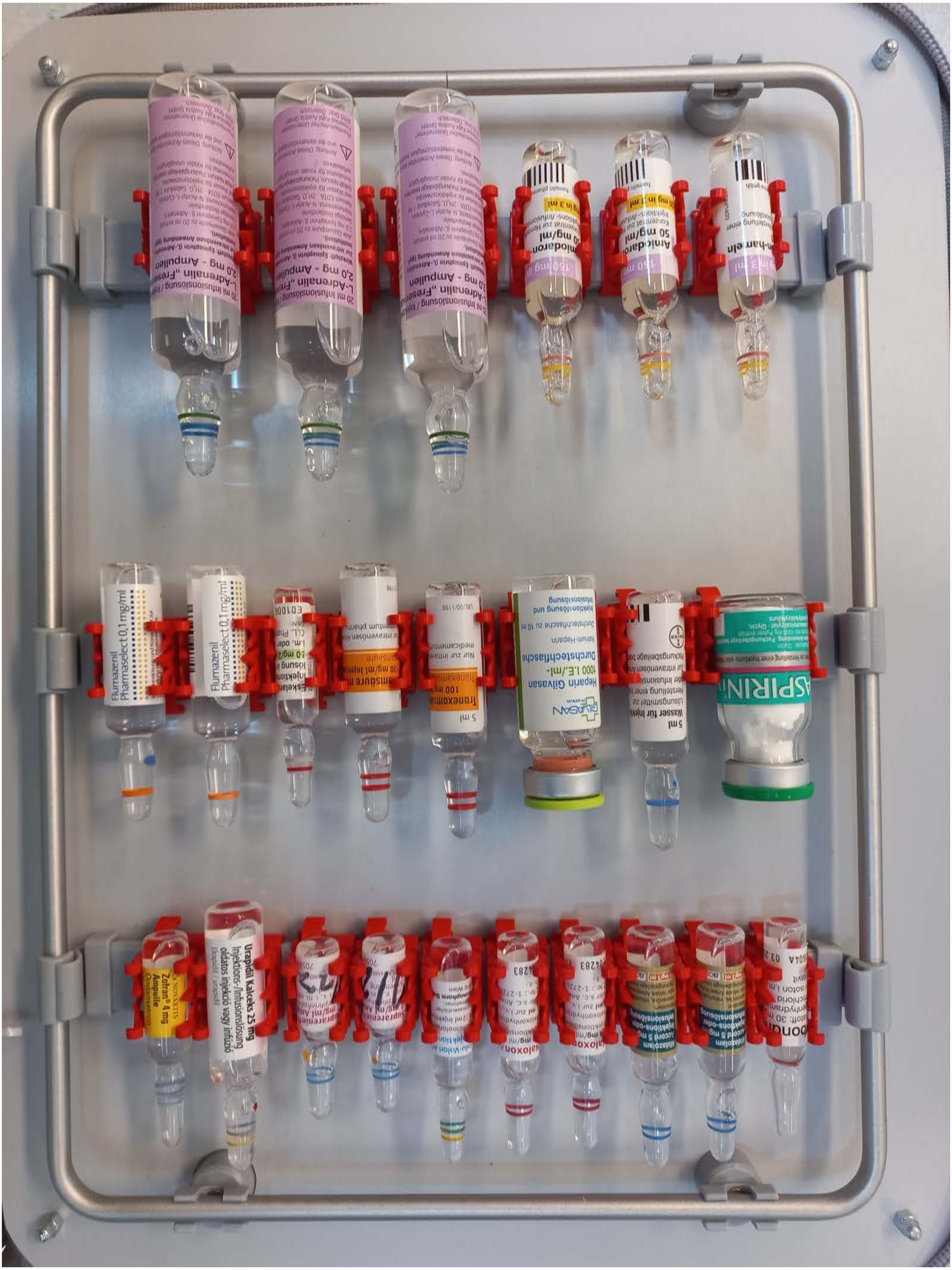
20 ml Infusionslösung
L-Adrenalin „Fresenius"
2,0 mg - Ampullen
Amiodaron
50 mg/ml
Flumazenil
Pharmaselect 0,1 mg/ml
Tranexamsäure
Heparin Gilvasan
1000 I.E./ml-
Durchstechflasche
5 ml
Wasser für Injektionszwecke
ASPIRIN
Zofran® 4 mg
Ampulle
Urapidil Kalceks 25 mg
Injektions-/Infusionslösung
Midazolam
Accord 5 mg/ml

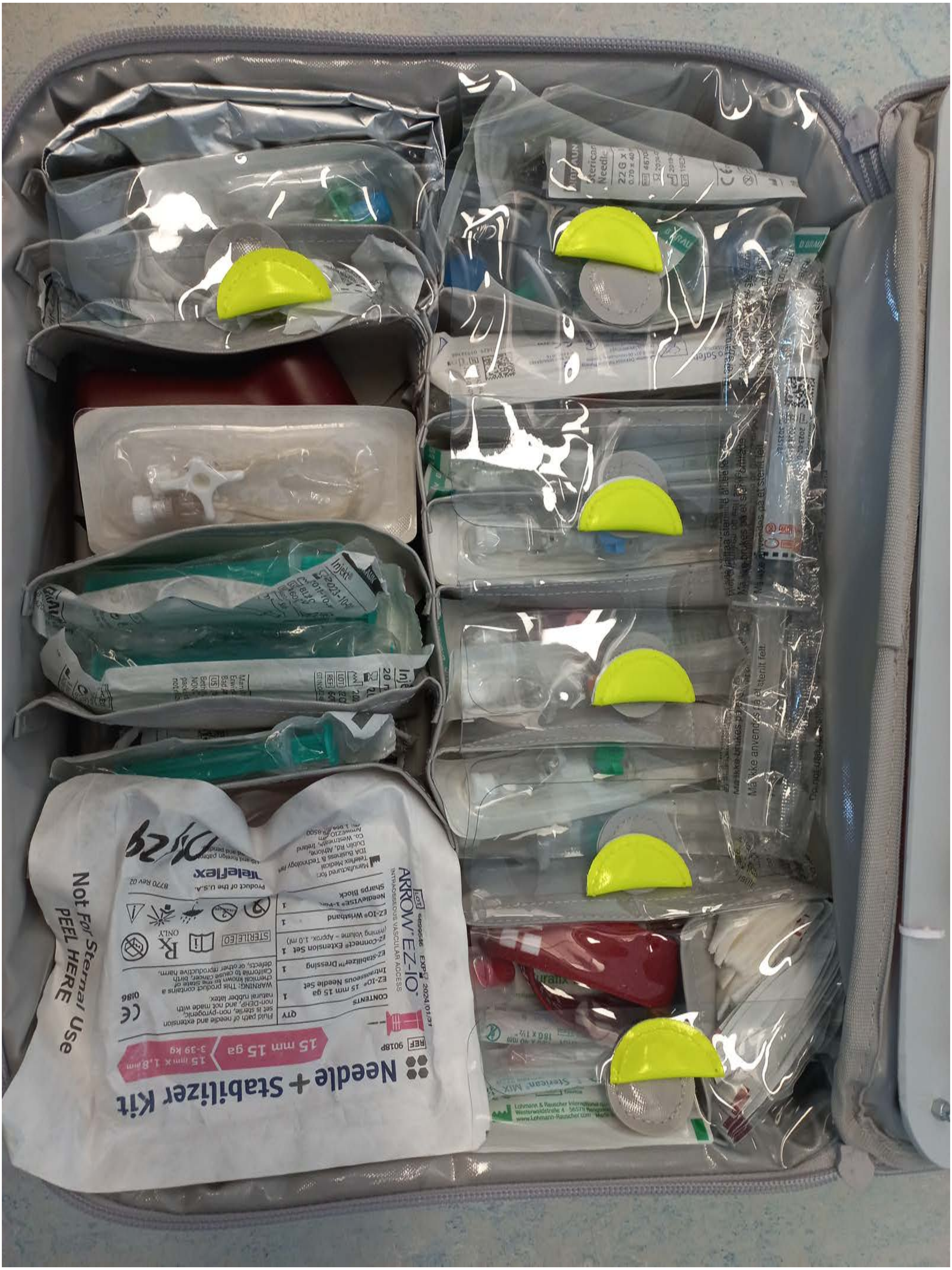
Needle + Stabilizer Kit
15 mm 15 ga
Not For Sternal Use
PEEL HERE
Teleflex
ARROW EZ-IO

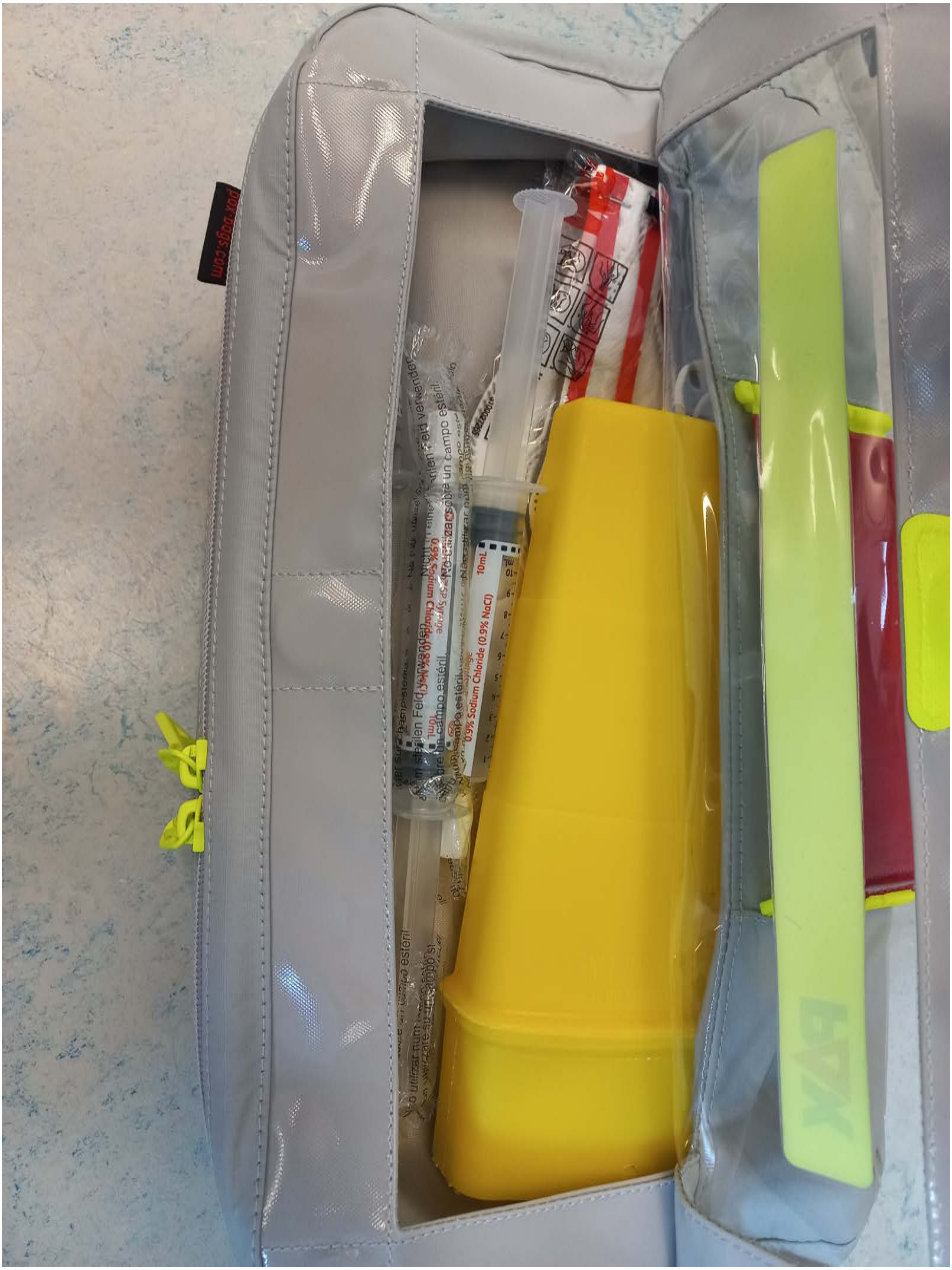
pax-bags.com
0.9% Sodium Chloride (0.9% NaCl)
10mL

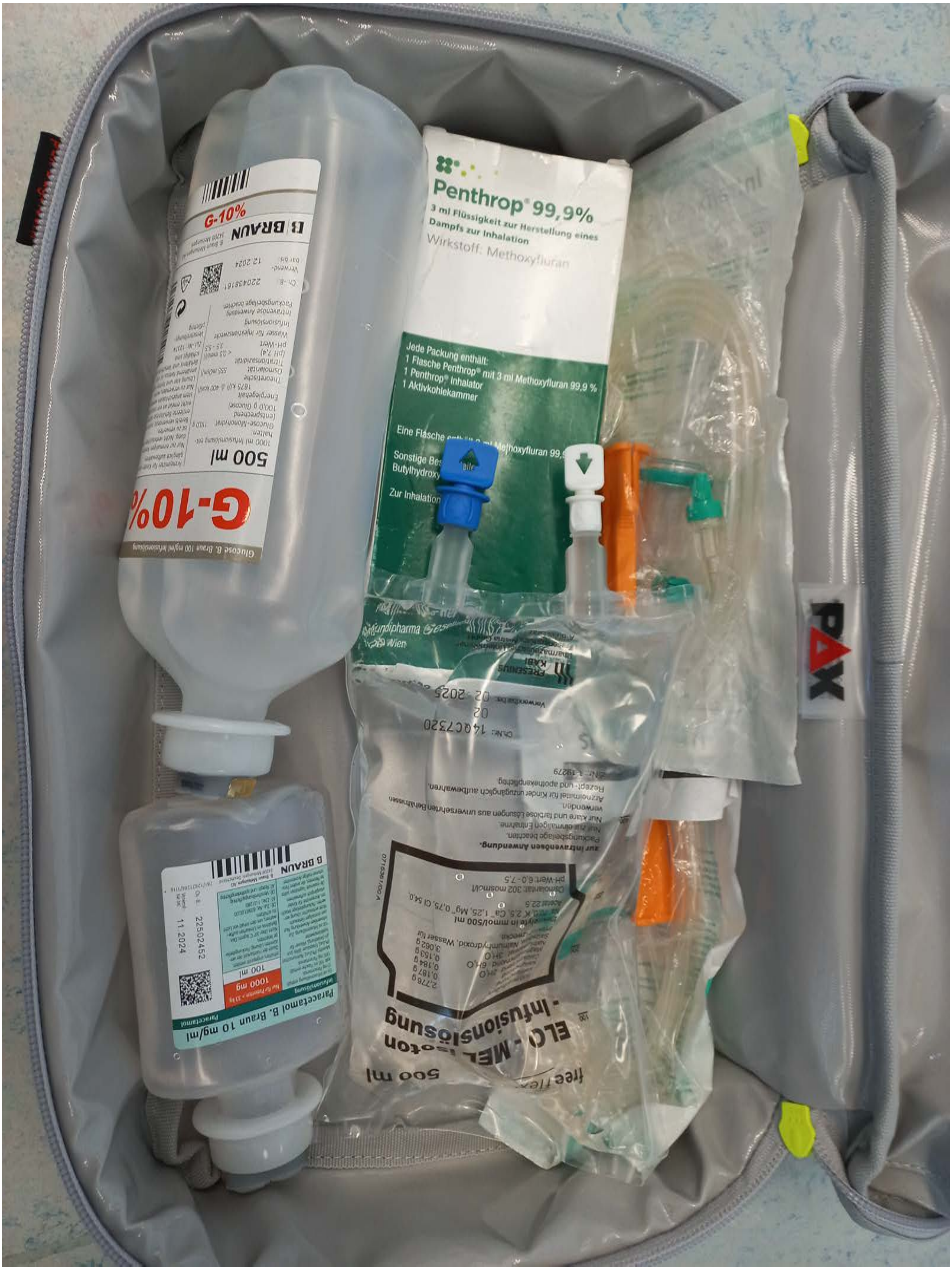
G-10%
B. BRAUN
500 ml
Penthrop® 99,9%
3 ml Flüssigkeit zur Herstellung eines Dampfs zur Inhalation
Wirkstoff: Methoxyfluran
Jede Packung enthält:
1 Flasche Penthrop® mit 3 ml Methoxyfluran 99,9 %
1 Penthrop® Inhalator
1 Aktivkohlekammer
Paracetamol B. Braun 10 mg/ml
1000 mg
100 ml
ELO-MEL isoton
- Infusionslösung
500 ml
PAX

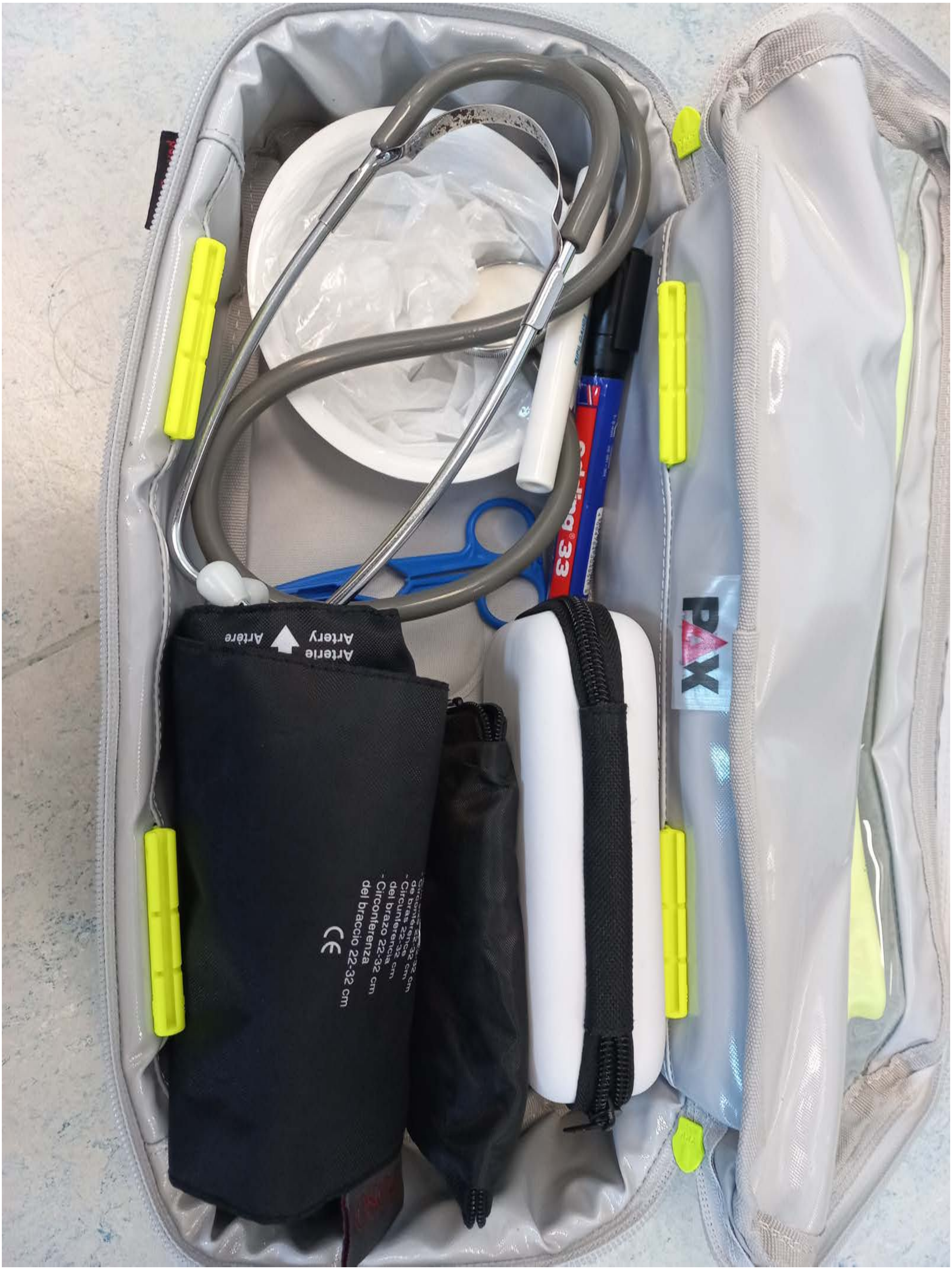
PAX
Artery
Arterie
Artère
Circonferenza
del braccio 22-32 cm

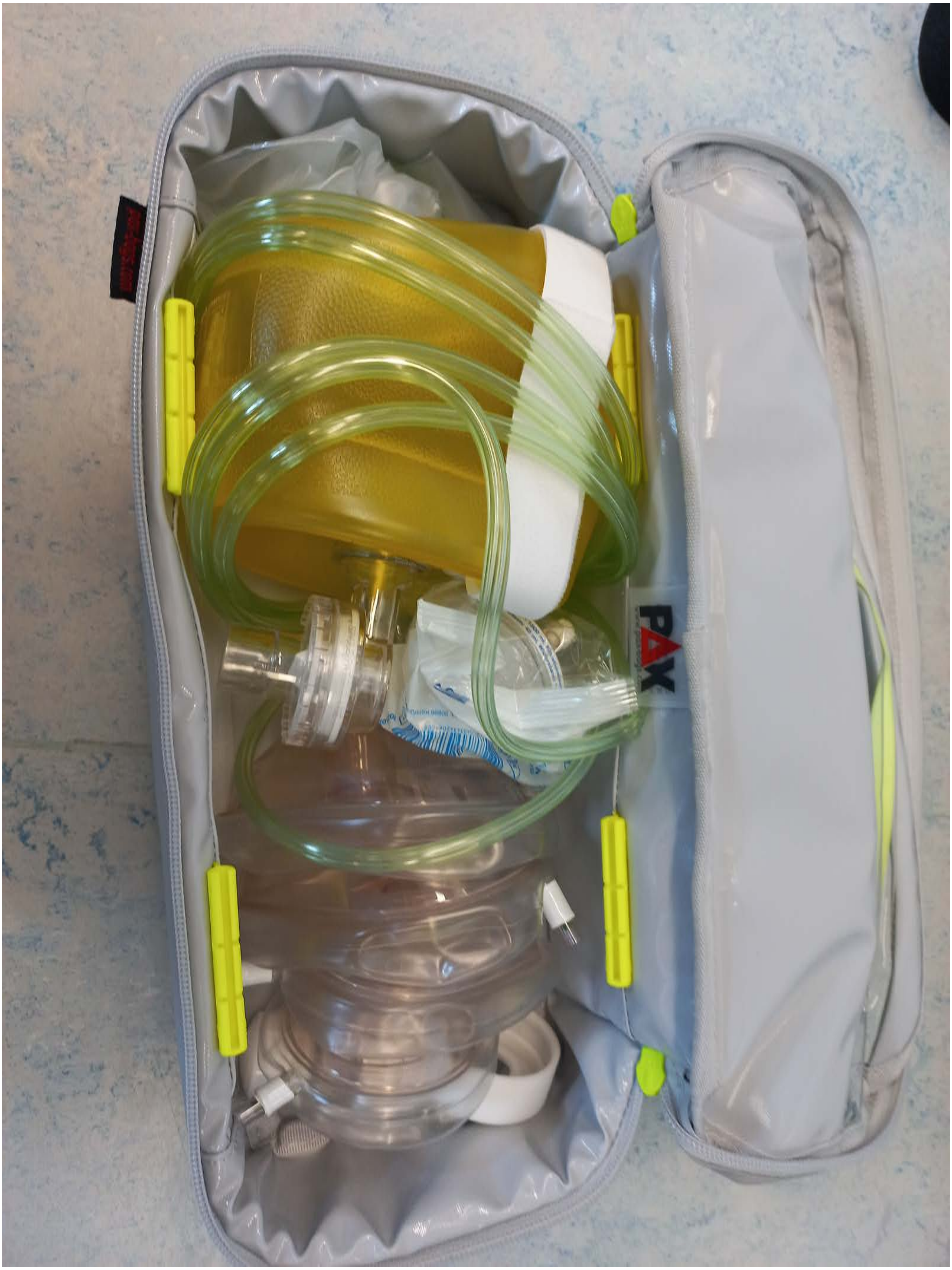
PAX

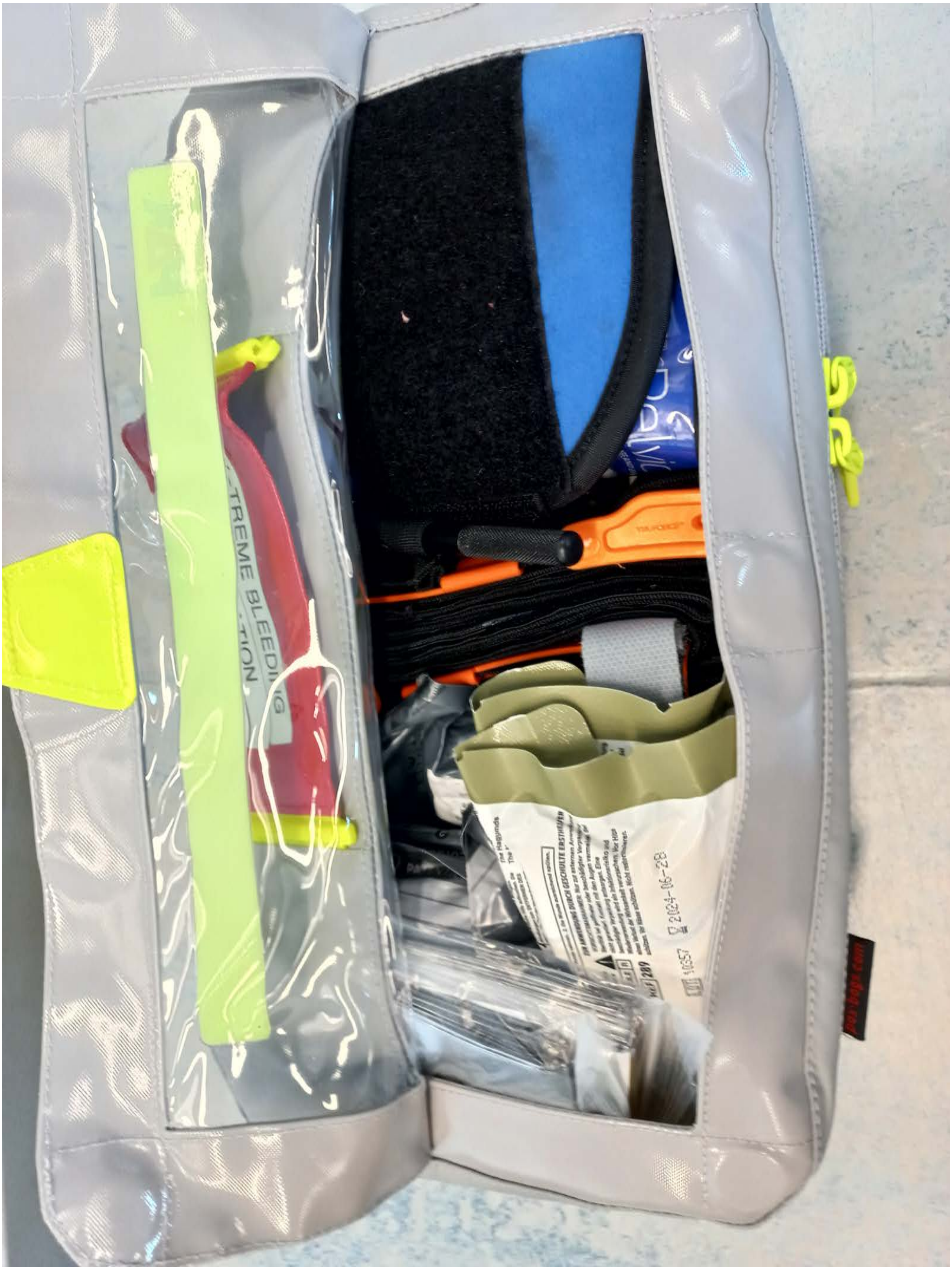

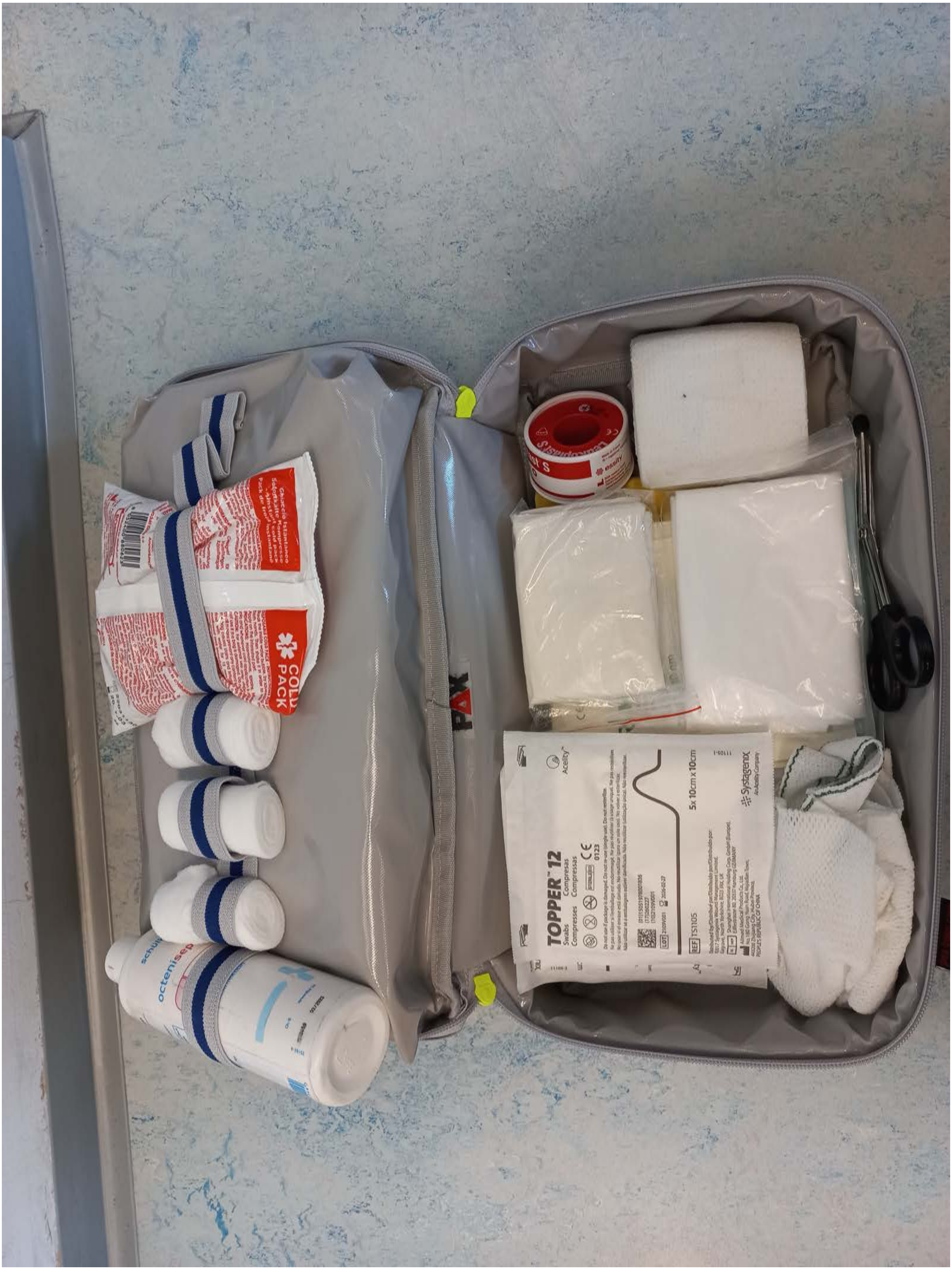
COLD PACK
PAX
TOPPER™ 12
Swabs
Compresses
5x 10cm x 10cm
Systagenix
Acelity
octenisept

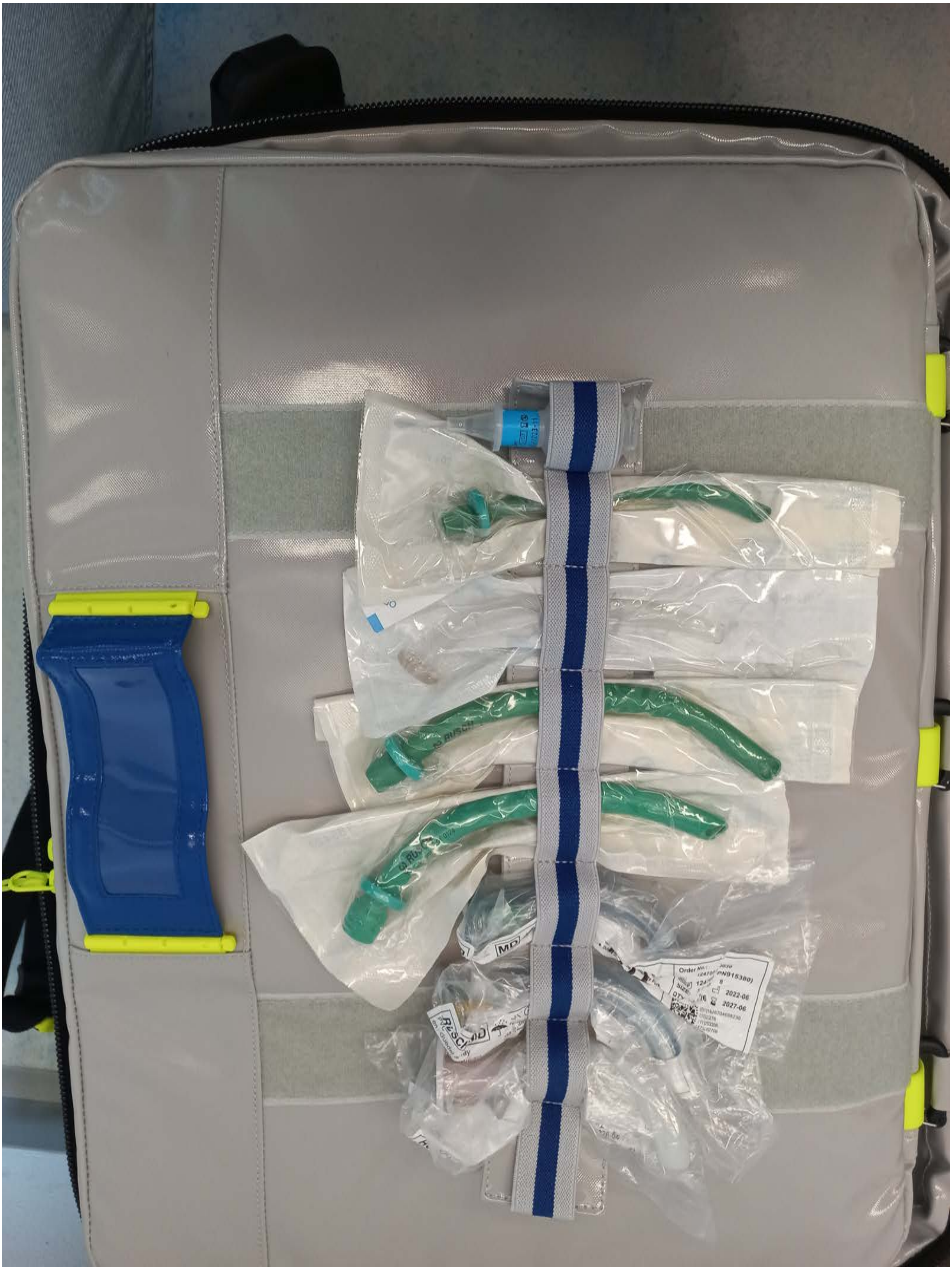
Order No.
2022-06
2027-06
