# Supplementary material for: Conventional versus task-based package organization for out-of-hospital emergency kits: an emergency medical services simulation study
Source: Scand J Trauma Resusc Emerg Med. 2024 Dec 20;32:135. doi: 10.1186/s13049-024-01309-8 (PMC11660938; doi:10.1186/s13049-024-01309-8)

Novel kit (non-TPO)

Ampullarium (top half)	
medication compartment	various i.v. medications<br>rectal midazolam<br>flush syringes<br>various non-i.v. medications<br>blunt needles
infusion compartment	crystalloid infusion<br>glucose infusion<br>methoxyflurane inhaler<br>i.v. lines
Ampullarium (lower half)	
left compartments	syringe connector<br>spike adapter<br>3-way stopcocks<br>syringe caps<br>tourniquet<br>i.v.-catheters (various sizes)
right fold-away compartment	alcoholic swabs<br>dry gauze<br>i.v.-dressings
right compartments	syringes (various sizes)<br>paracetamol (100mL)<br>hypodermic needles<br>MAD adapters
Diagnostics	
left compartment	pelvic binder<br>antiseptic spray
central compartment	ear thermometer<br>blood-pressure cuff<br>pen light<br>stethoscope<br>glucose measurement set
right compartment	intraosseous drill<br>intraosseous needle-kits (various sizes)<br>flush syringes<br>3-way stopcock

Sharps container
Foamed aluminum splint

Bandages	plasters
	scissors
	elastic bandage
Dressings	emergency blankets
	triangular bandages
	various wound dressings
Trauma	adhesive tape
	cool packs
	emergency bandage
	hemostatic dressing
	self-adhesive bandage

Tourniquets
Trauma shears

Endotracheal tubes	stylet
	endotracheal tubes (various sizes)
	lubricant
Respiratory accessories	endotracheal tube fixation material
	10ml syringe
	extension tubing
Airway adjuncts	oropharyngeal Airways (various sizes)
	nasopharyngeal airways (various sizes)
Supraglottic Airway + Intubatior	i-gel (various sizes)
	lubricant
	blades (various sizes)
	video-laryngoscope
	vomit bag
BVM	self-inflating bag
	oxygen tubing
	oxygen resevoir
	masks (various sizes)
	PEEP valve
	respiratory filter (various sizes)

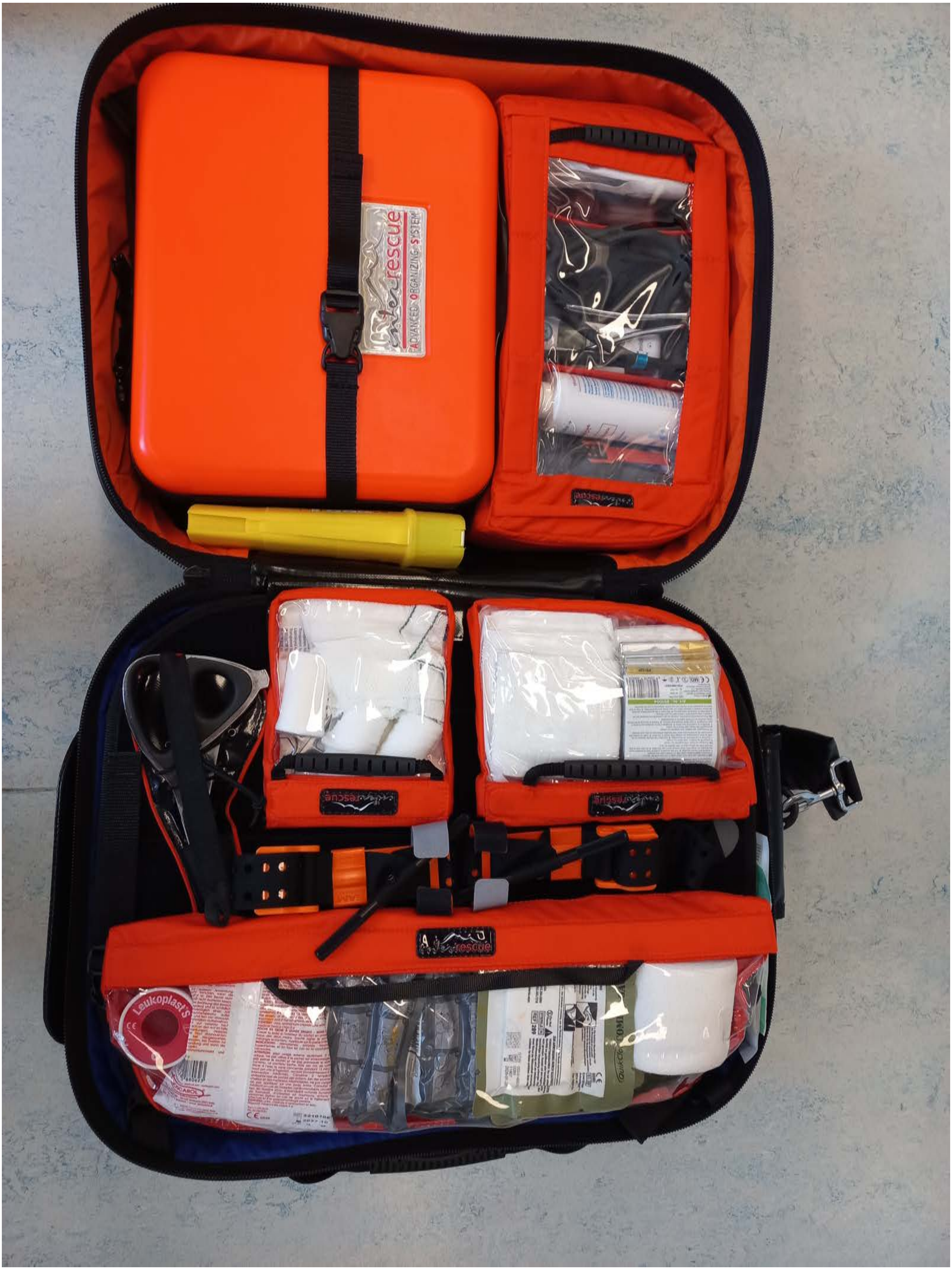
rescue
ADVANCED ORGANIZING SYSTEM
Leukoplast S

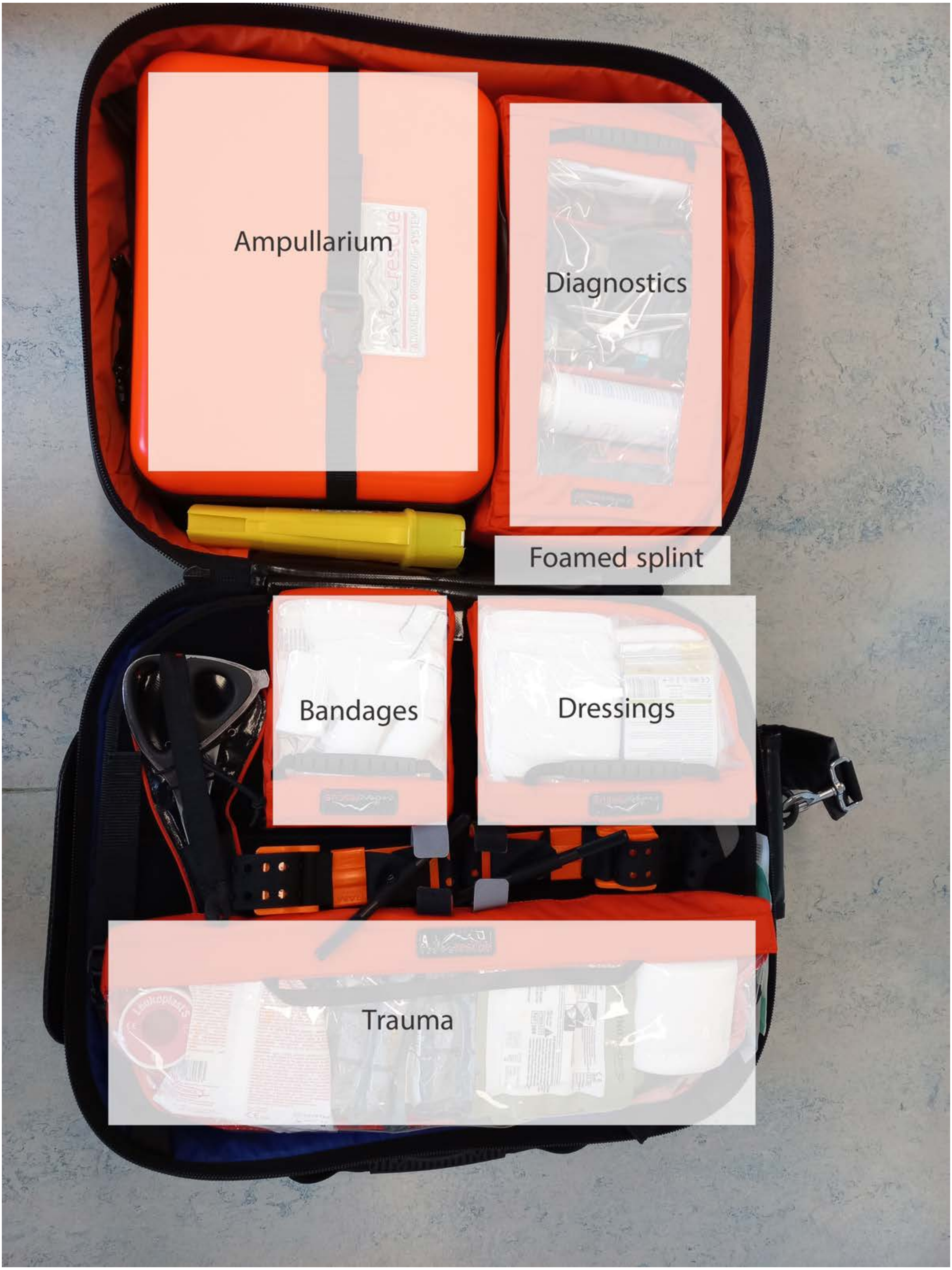
Ampullarium
Diagnostics
Foamed splint
Bandages
Dressings
Trauma

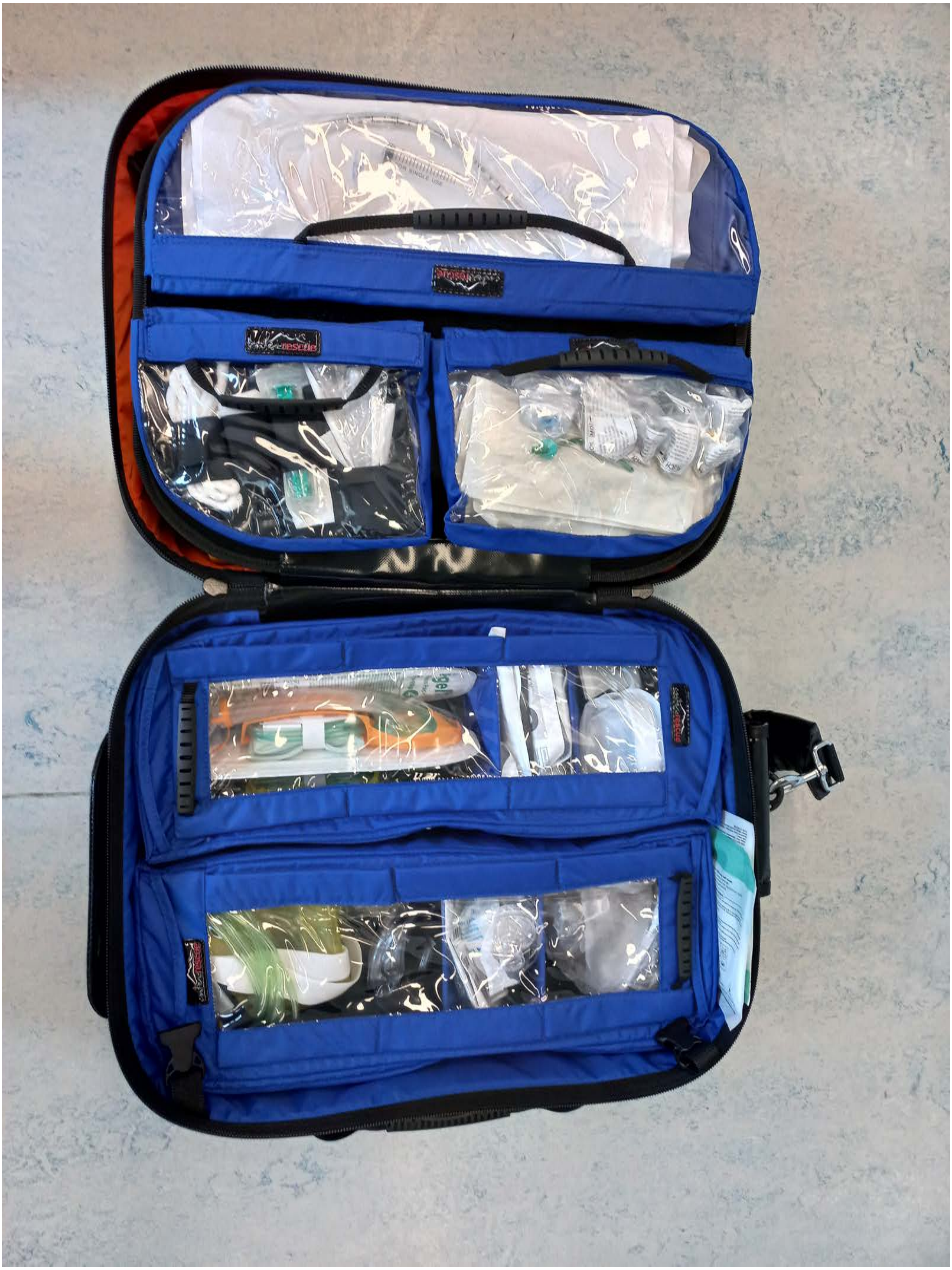

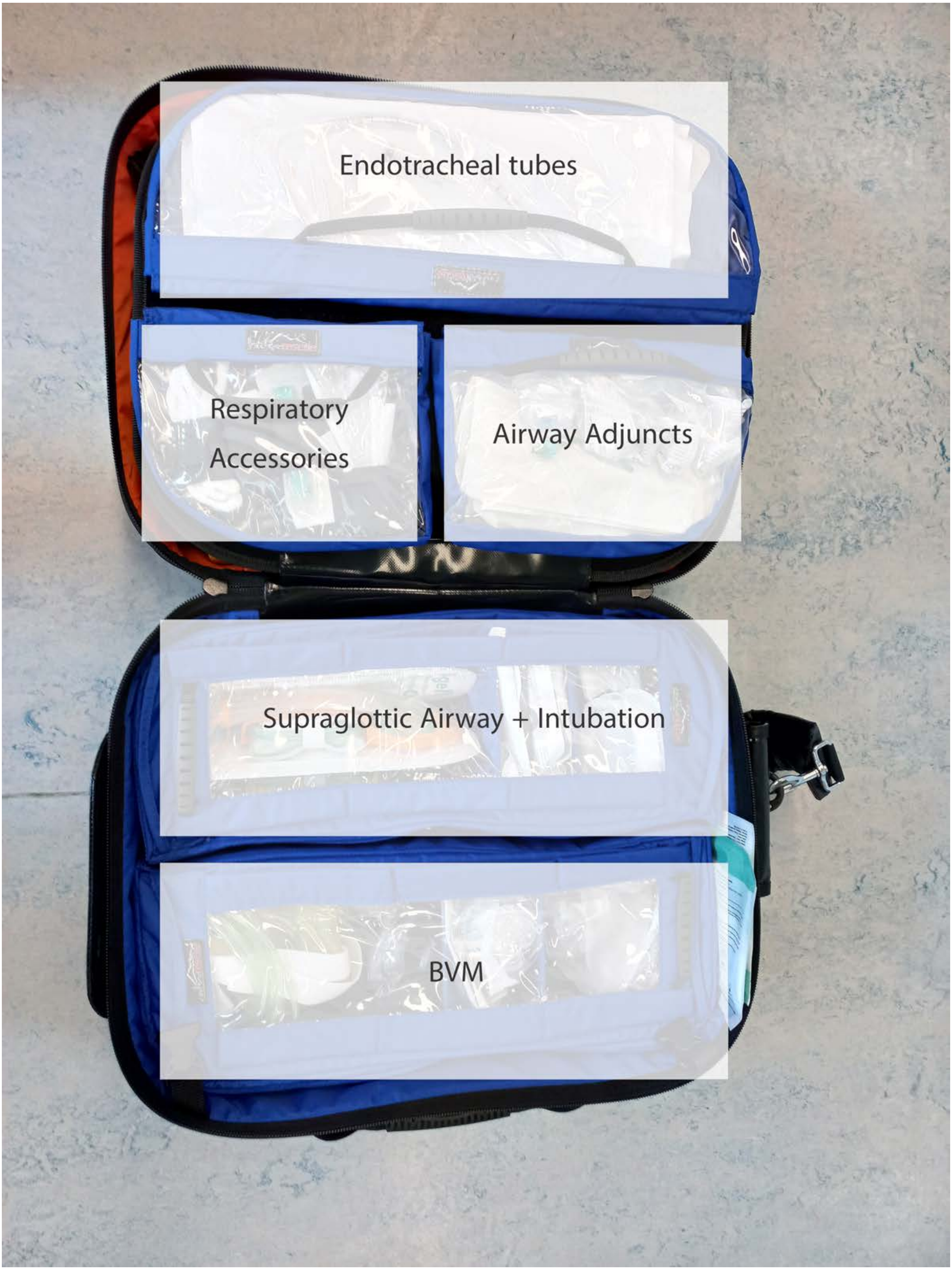
Endotracheal tubes
Respiratory Accessories
Airway Adjuncts
Supraglottic Airway + Intubation
BVM

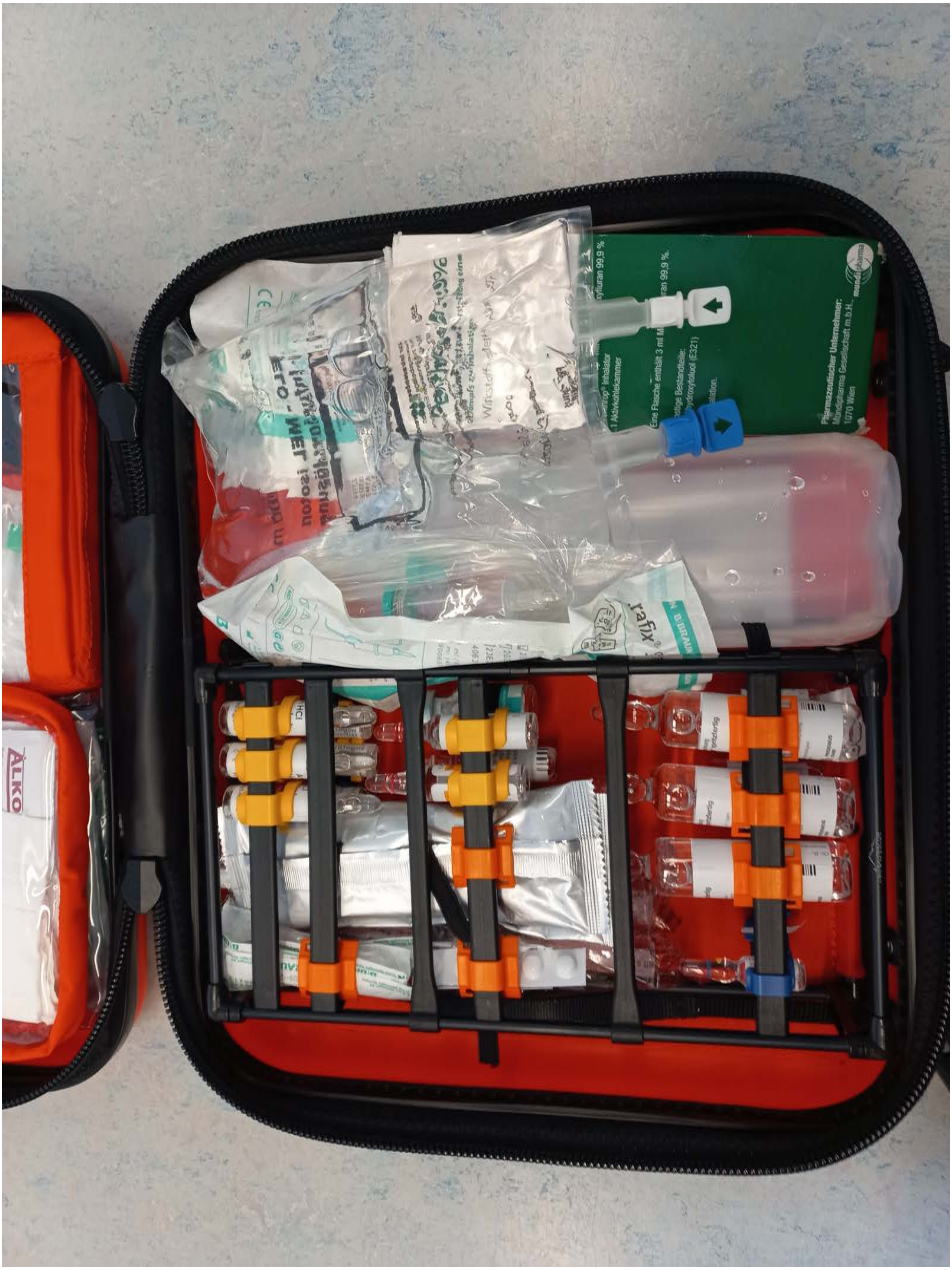

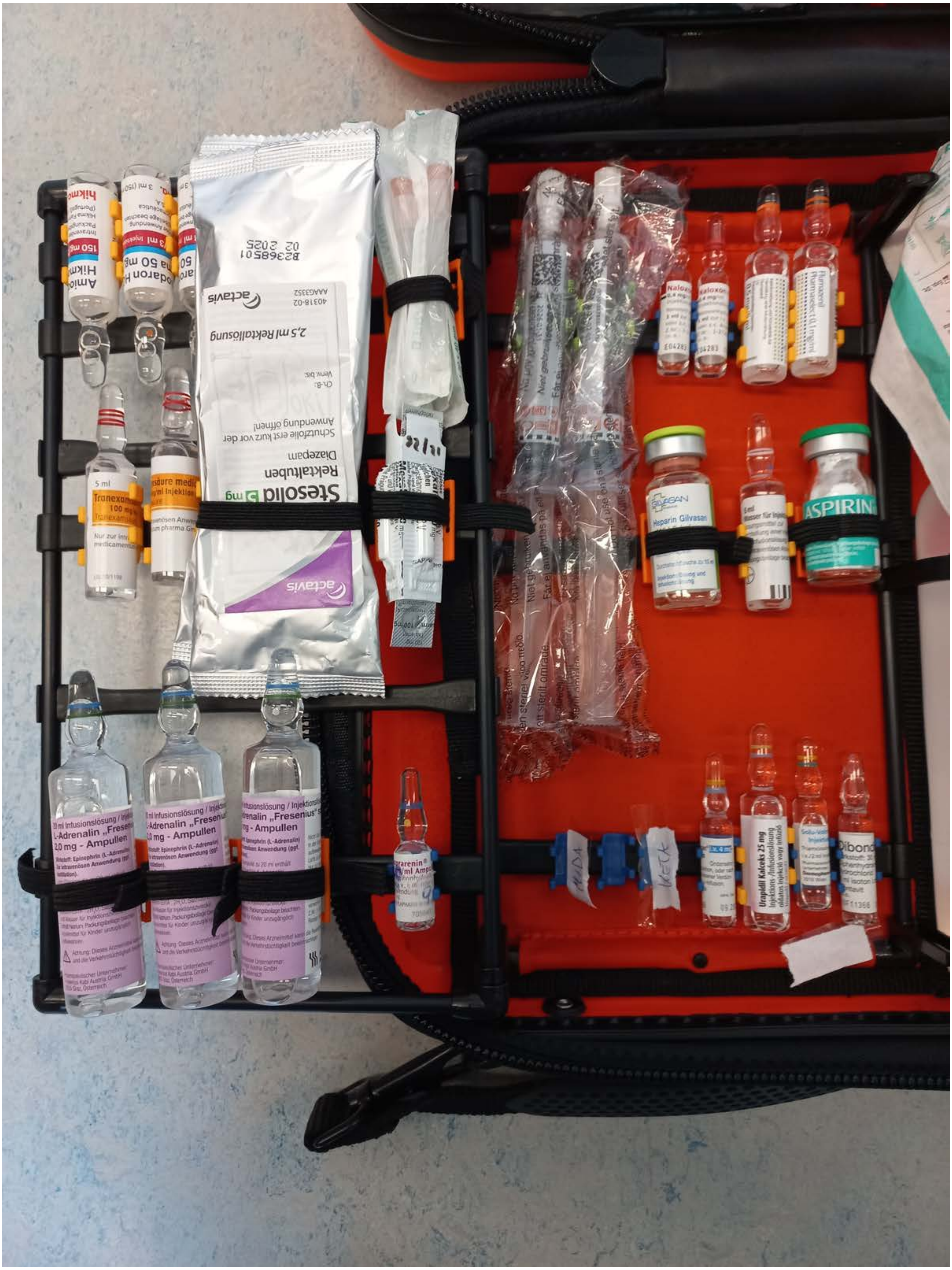
Stesolid 5 mg
Rektaltuben
Diazepam
Schutzfolie erst kurz vor der Anwendung öffnen!
2,5 ml Rektallösung
actavis
Heparin Gilvasan
ASPIRIN
Urapidil Kalceks 25 mg
L-Adrenalin „Fresenius"
1,0 mg - Ampullen
Tranexamsäure
Naloxon
Dibondrin

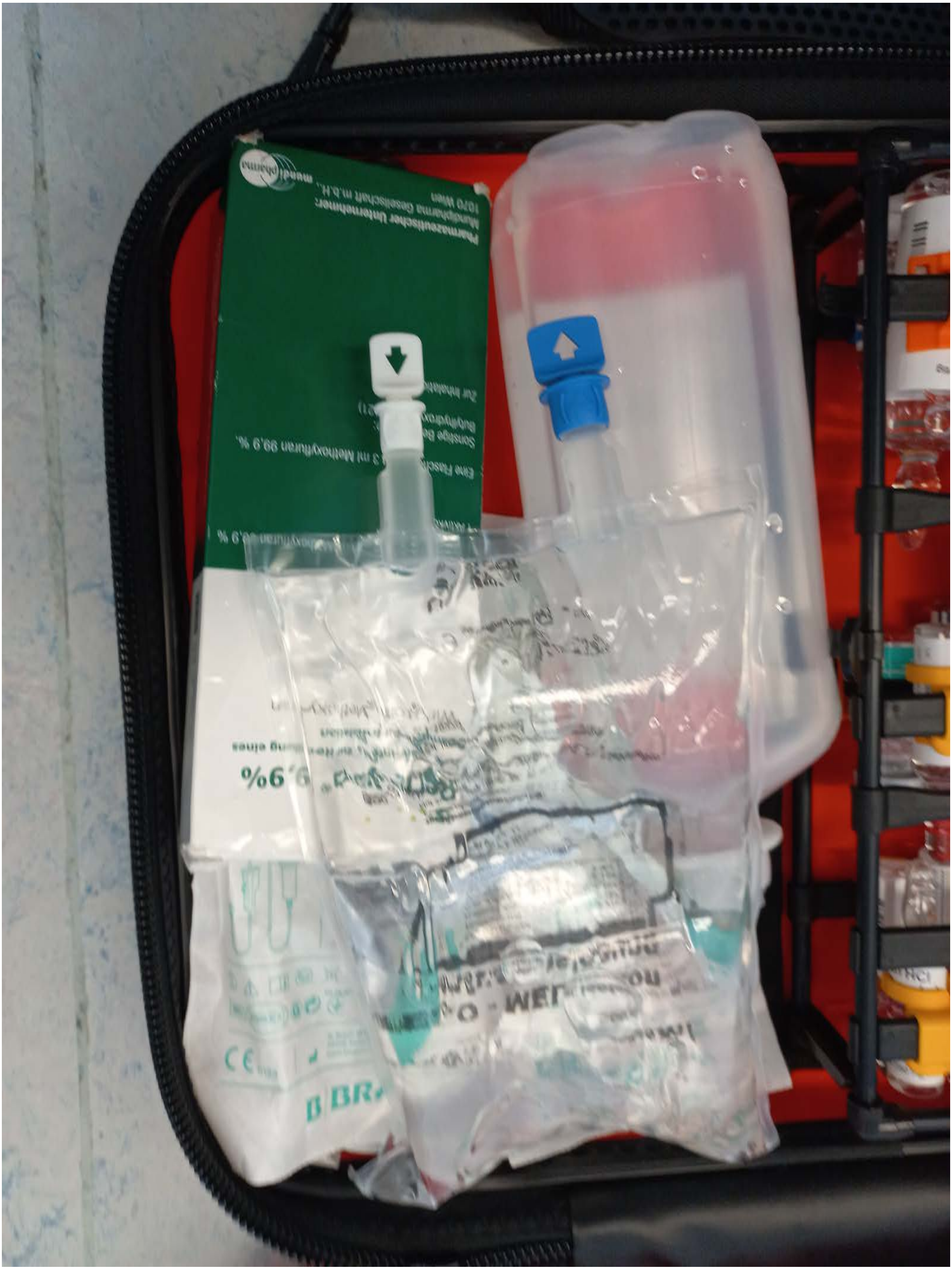
Pharmazeutischer Unternehmer:
Mundipharma Gesellschaft m.b.H.,
1070 Wien

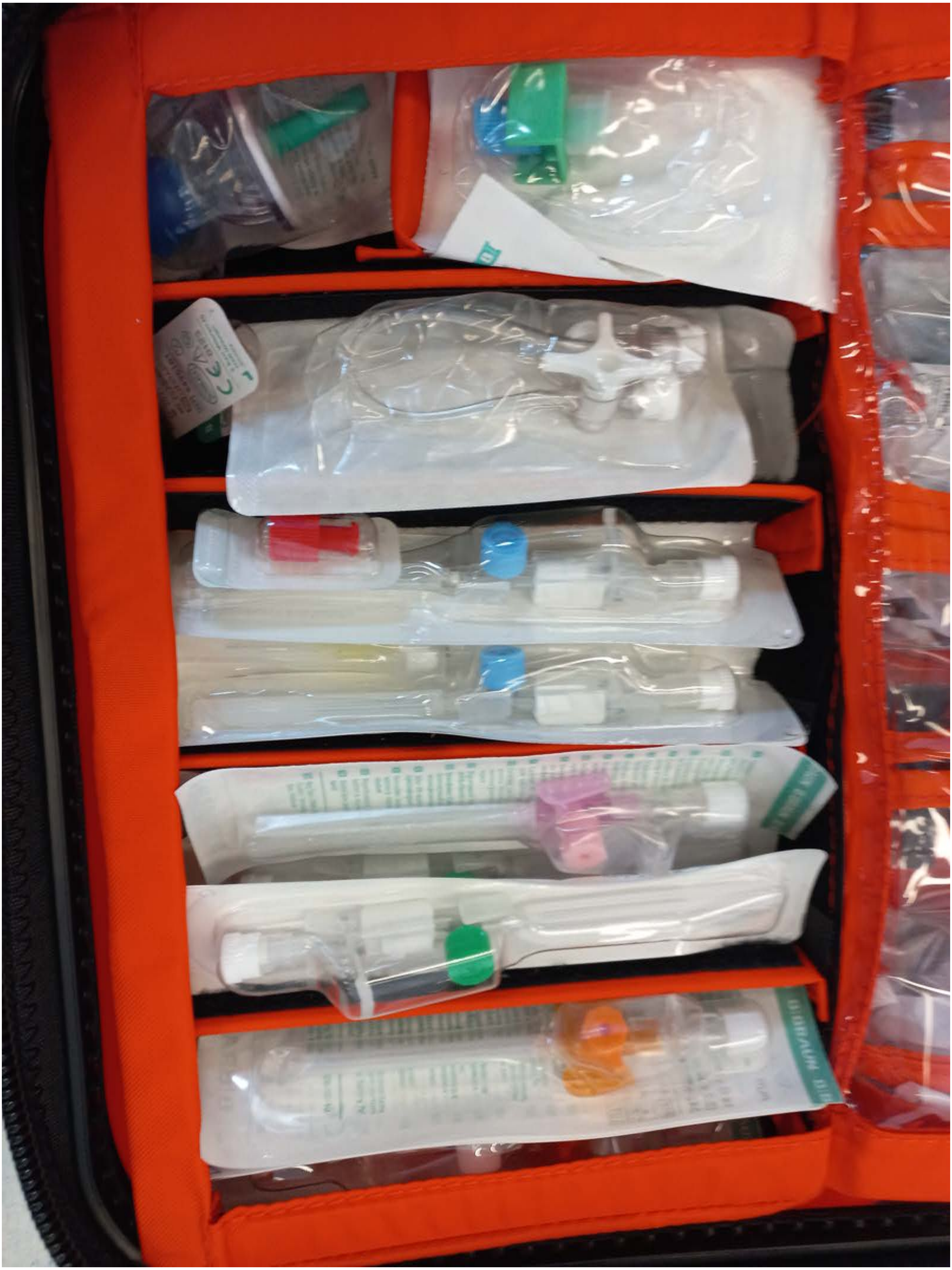

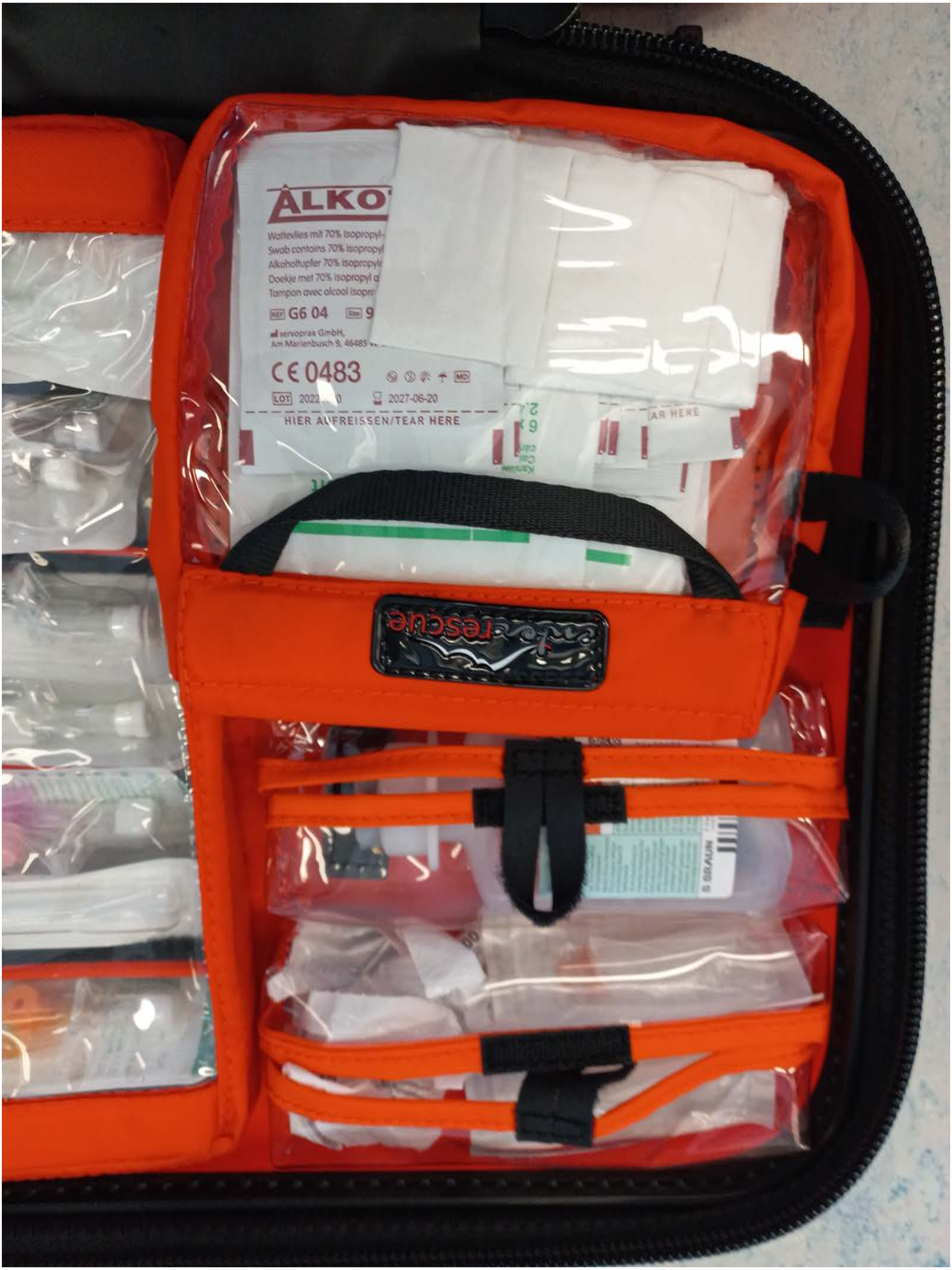
ALKO
Wattevlies mit 70% Isopropyl-
Swab contains 70% isopropyl
Alkoholtupfer 70% isopropyl
Doekje met 70% isopropyl a
Tampon avec alcool isopr
G6 04
servoprax GmbH,
Am Marienbusch 9, 46485 W
CE 0483
2027-06-20
HIER AUFREISSEN/TEAR HERE
rescue

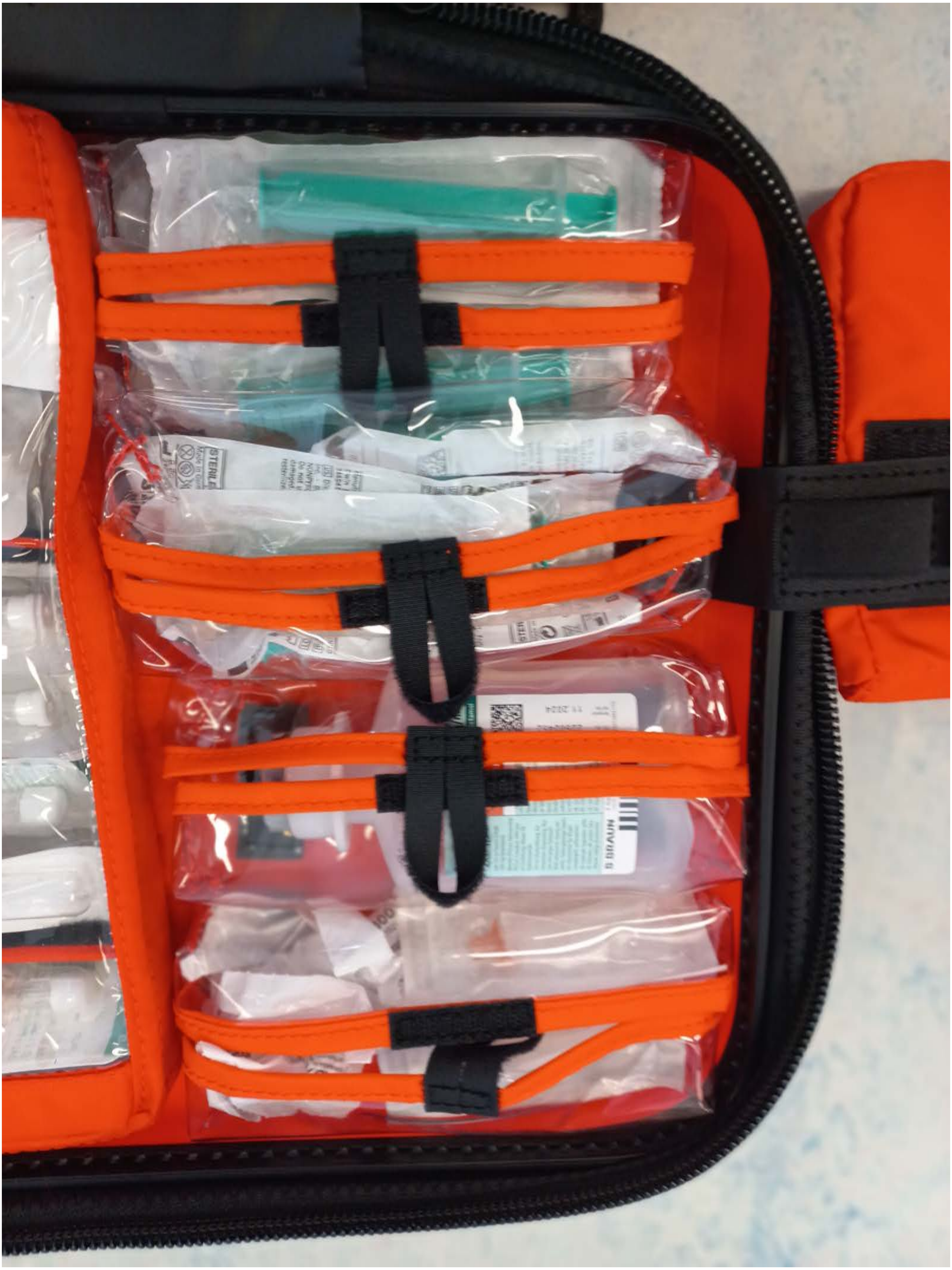

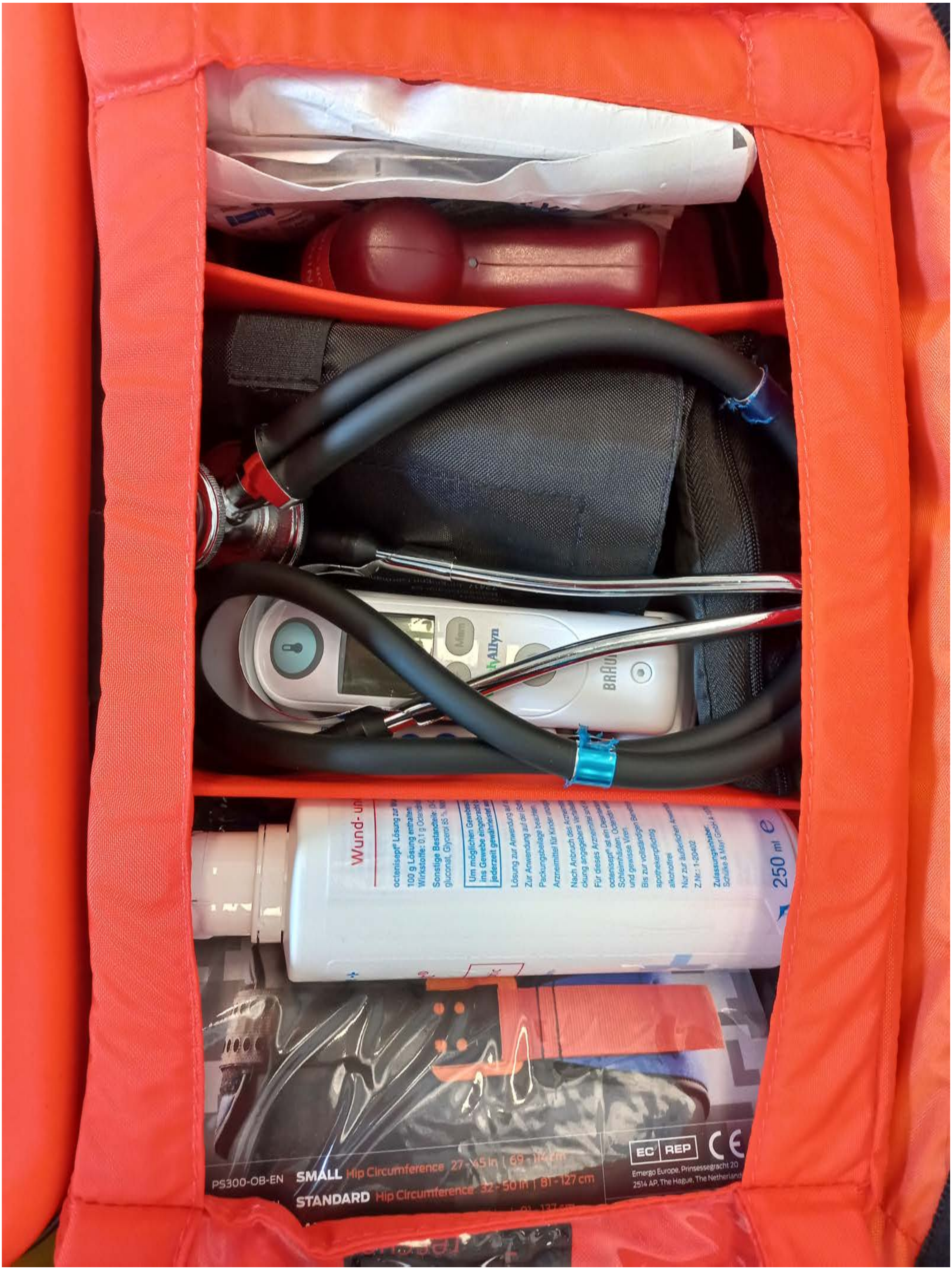

Mem
BRAUN
250 ml
PS300-OB-EN
SMALL Hip Circumference 27 - 45 in | 69 - 114 cm
STANDARD Hip Circumference 32 - 50 in | 81 - 127 cm
EC REP
Emergo Europe, Prinsessegracht 20

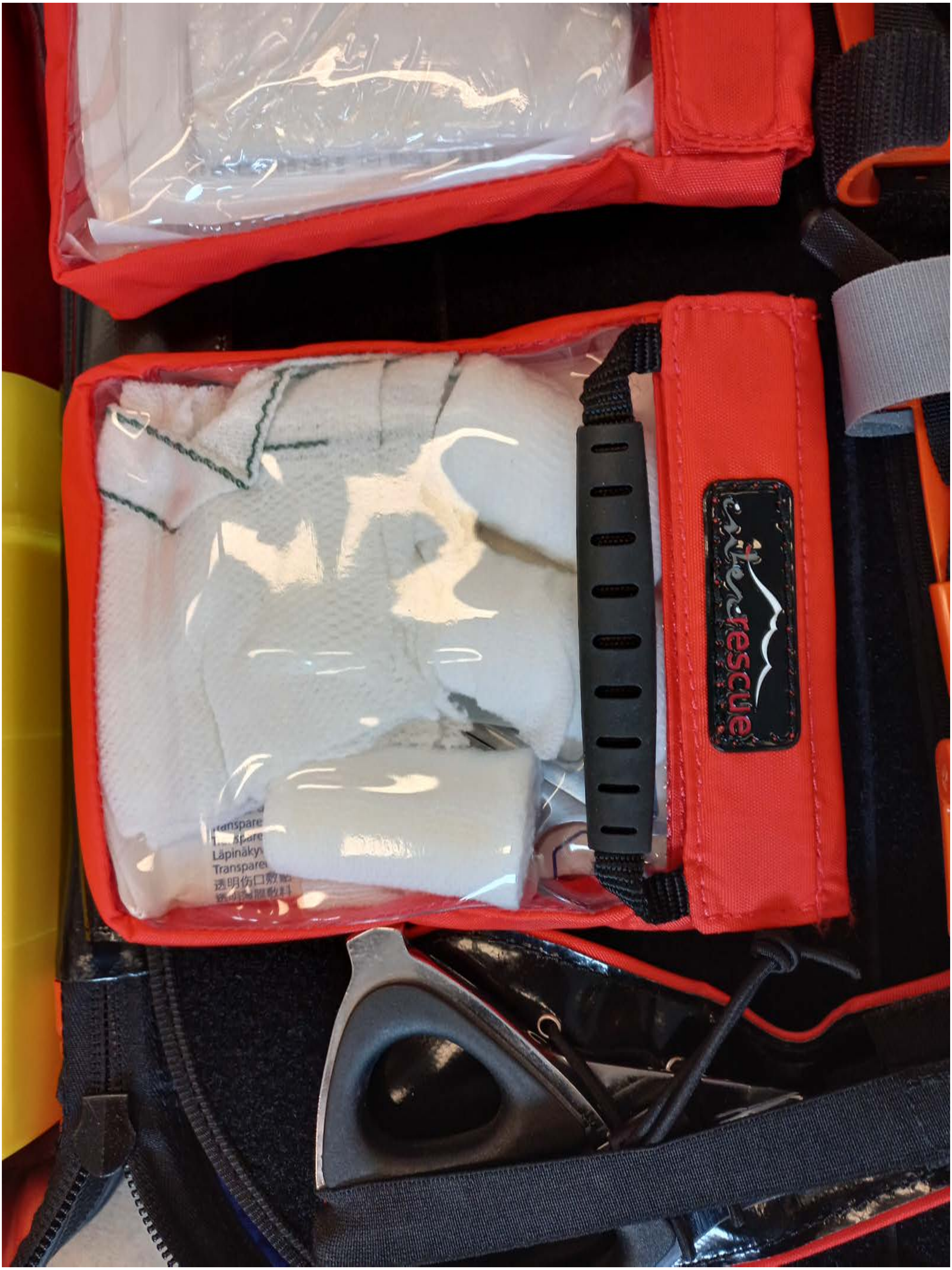
rescue

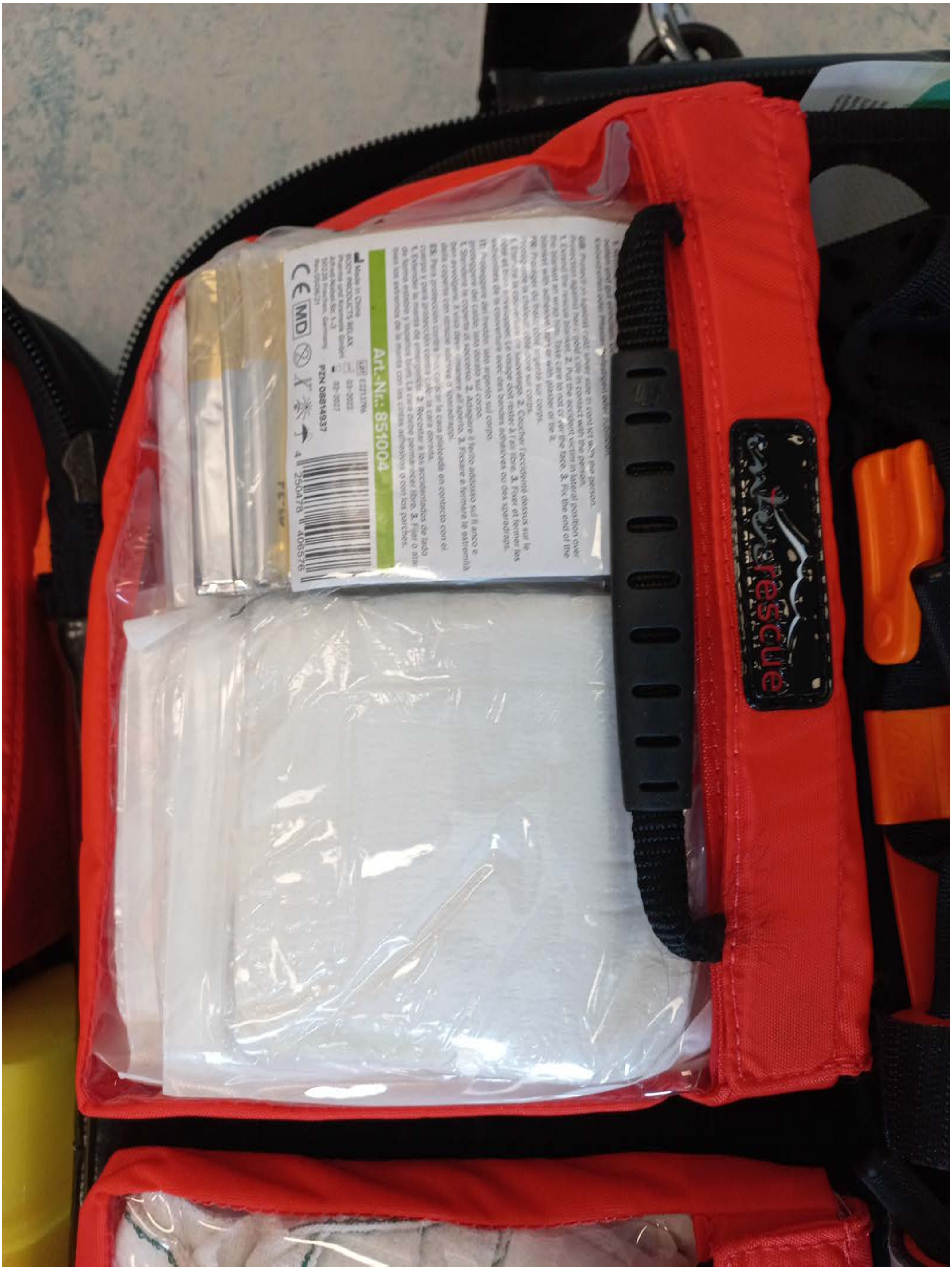
Art.-Nr.: 851004
rescue

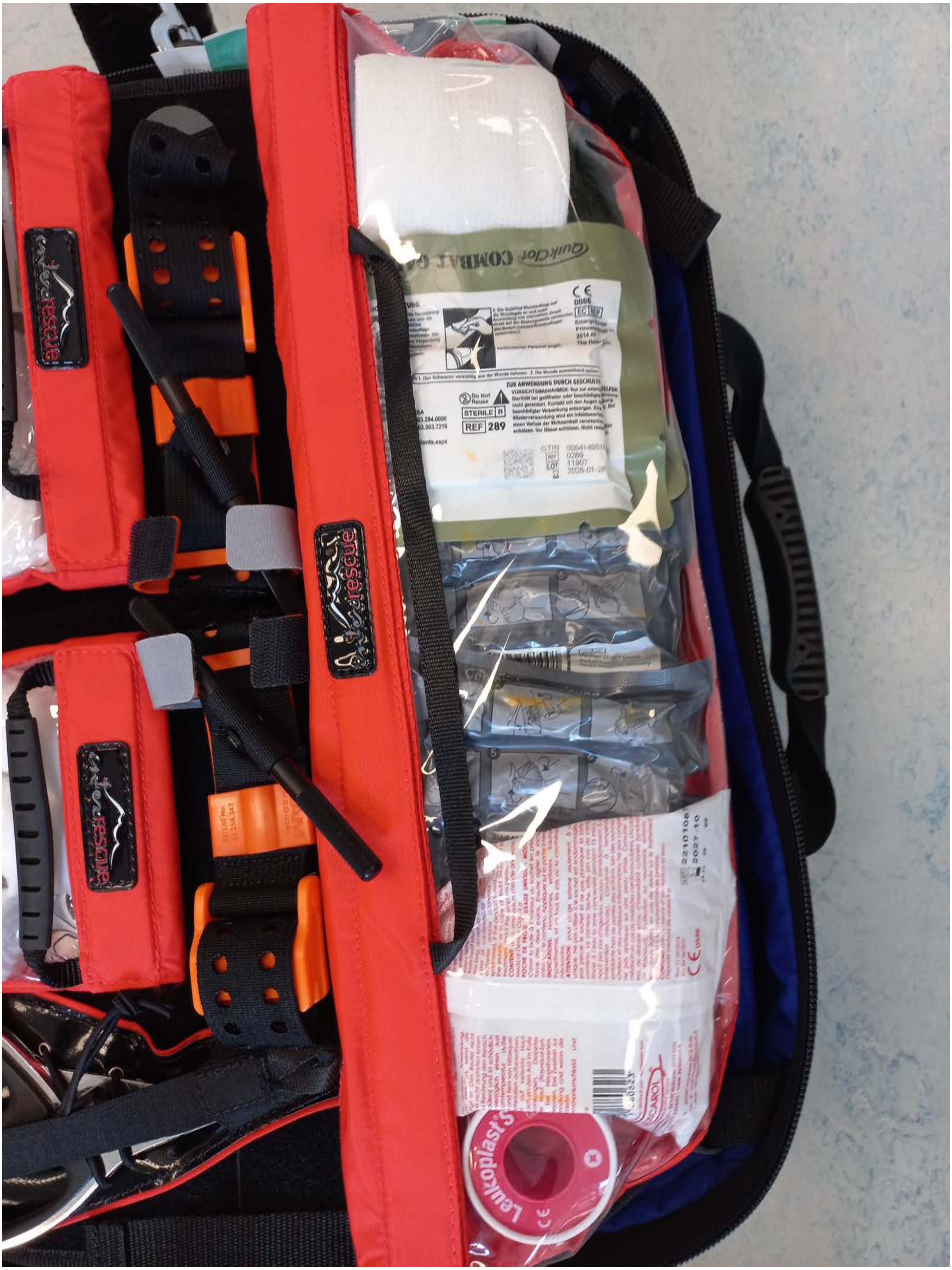
QuikClot
Do Not Reuse
STERILE R
REF 289
ZUR ANWENDUNG DURCH GESCHULTE
Leukoplast S

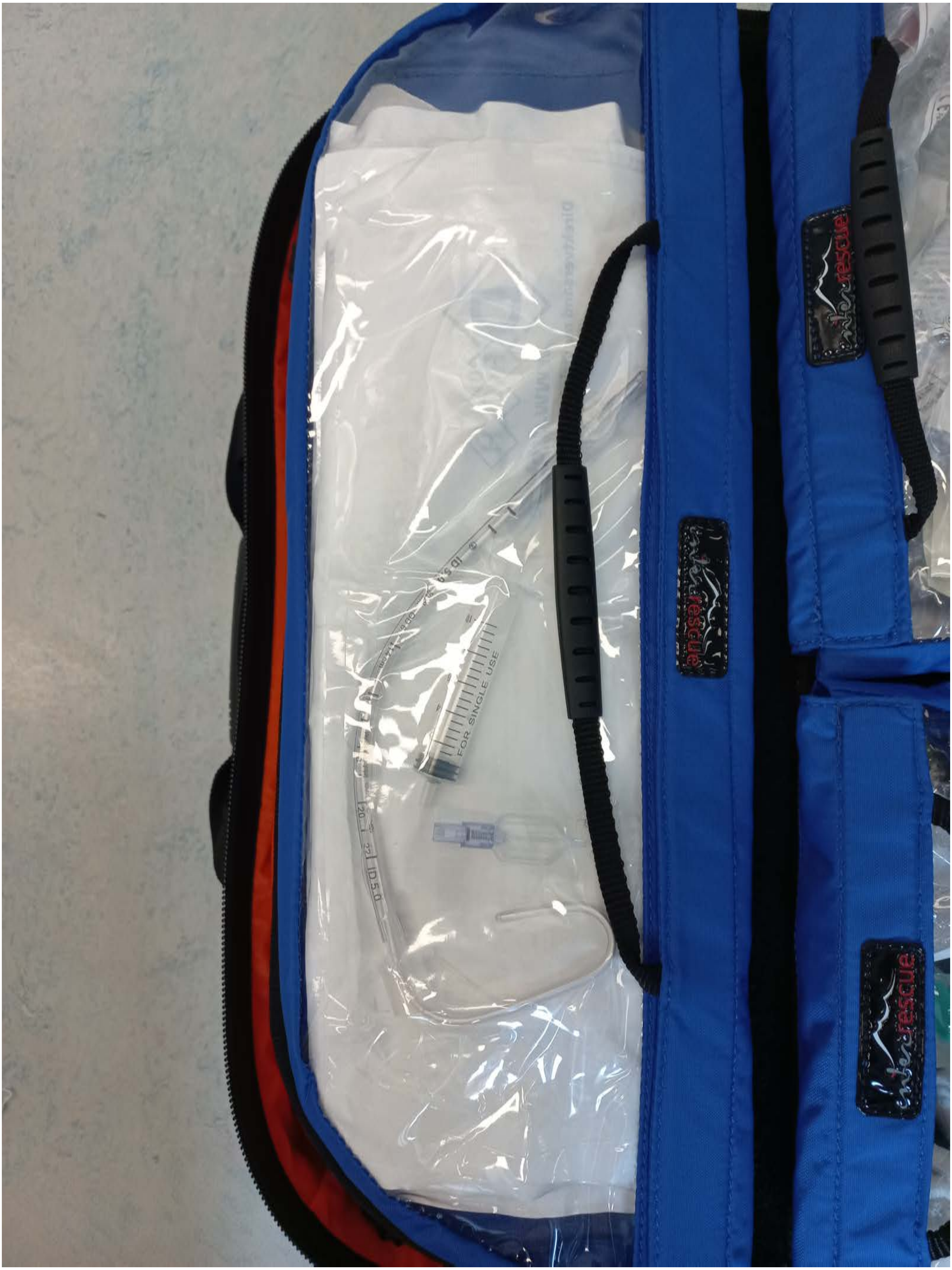
FOR SINGLE USE

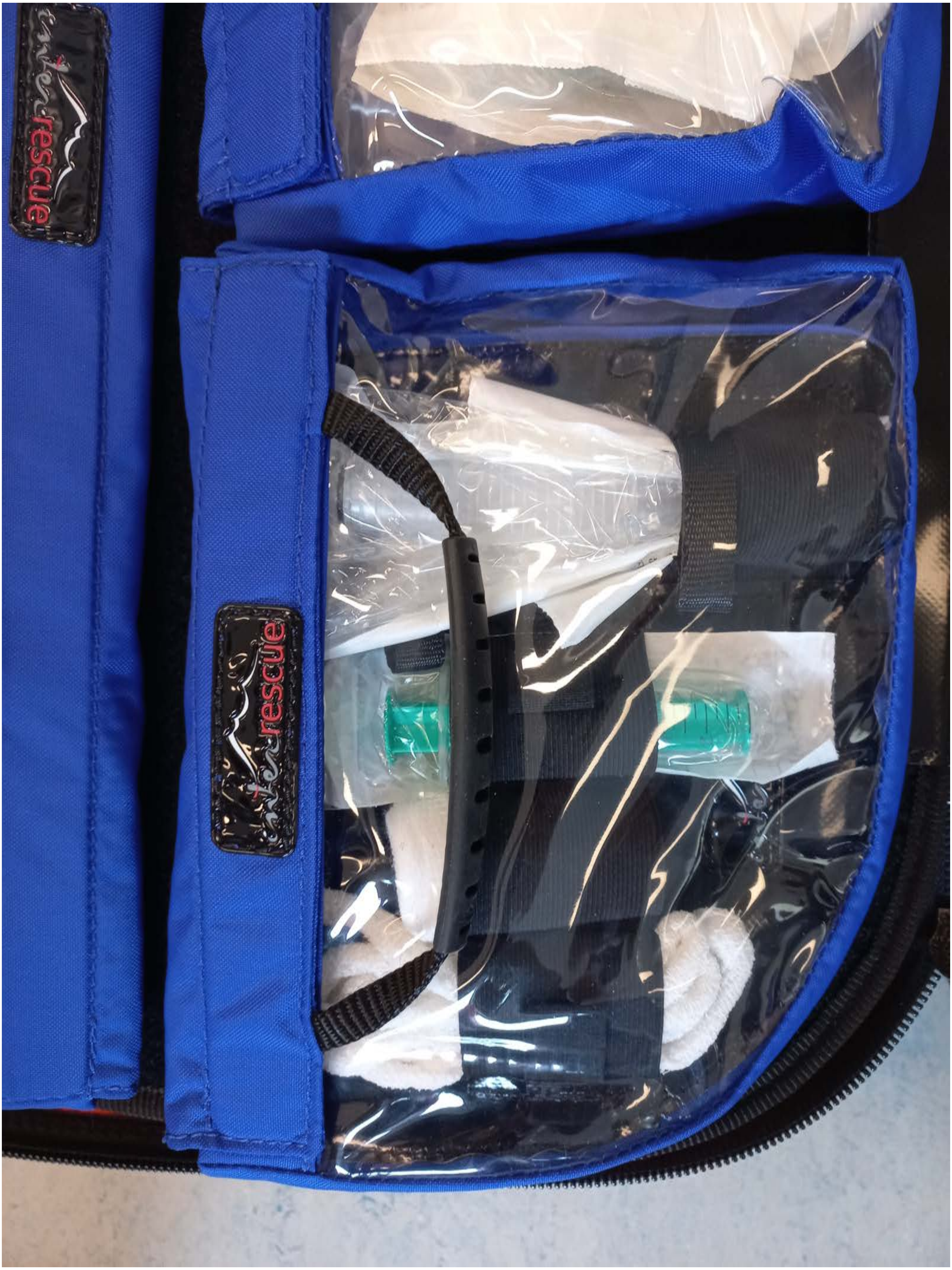
rescue
rescue

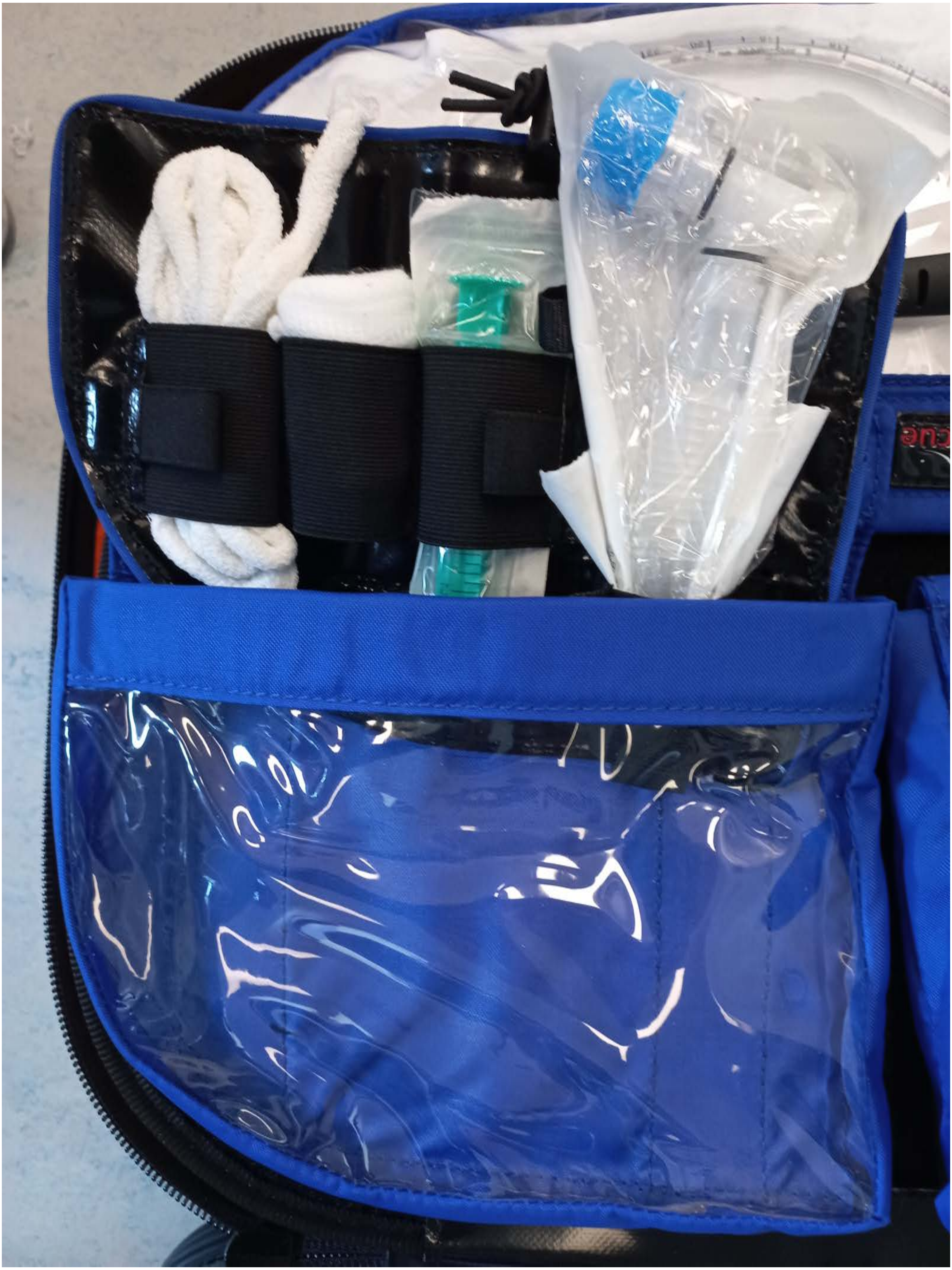

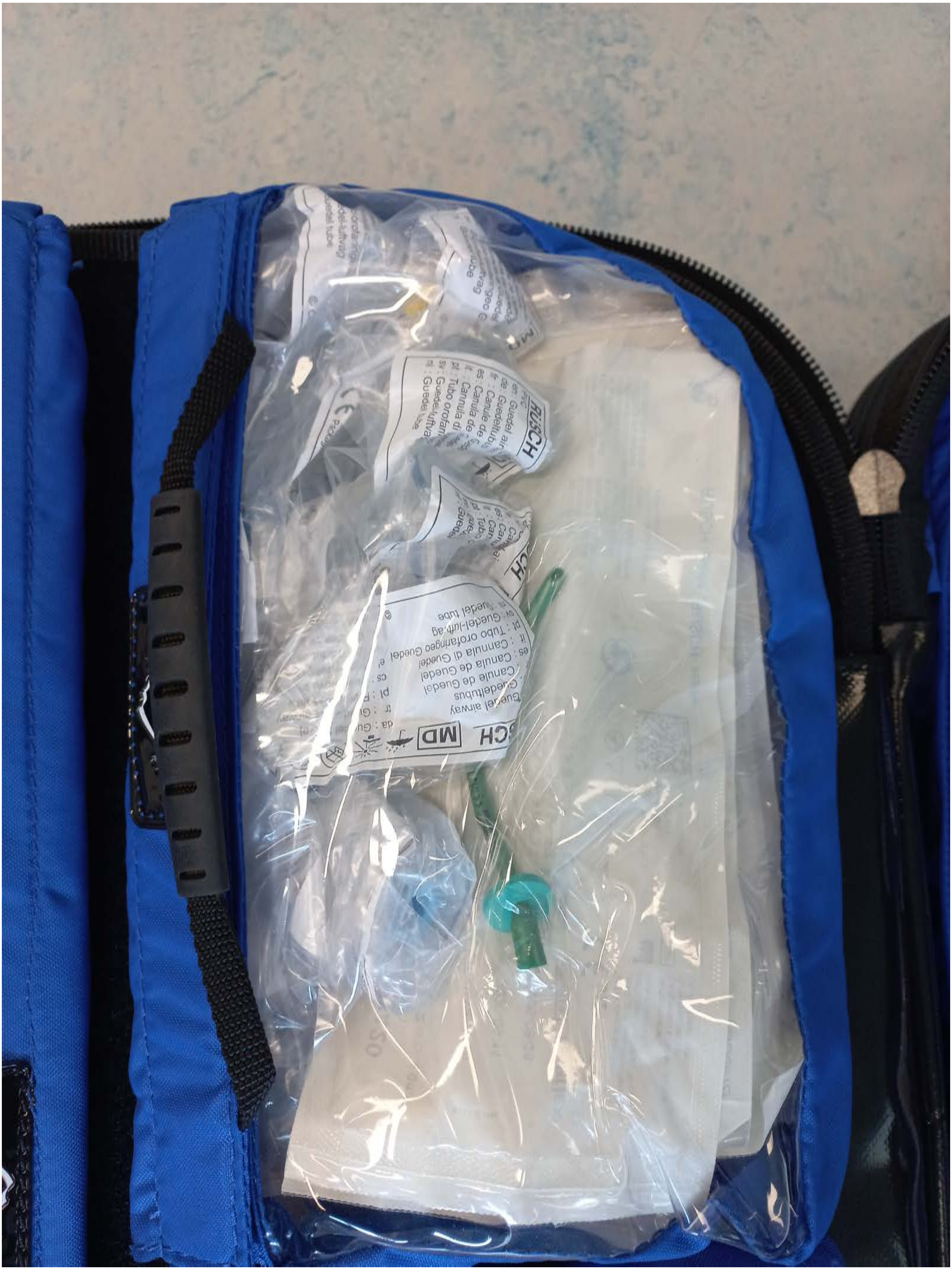
RÜSCH
MD
Guedel airway
Guedeltubus
Canule de Guedel
Canula de Guedel
Cannula di Guedel
Tubo orofaringeo Guedel
Guedel-luftväg
Guedel tube

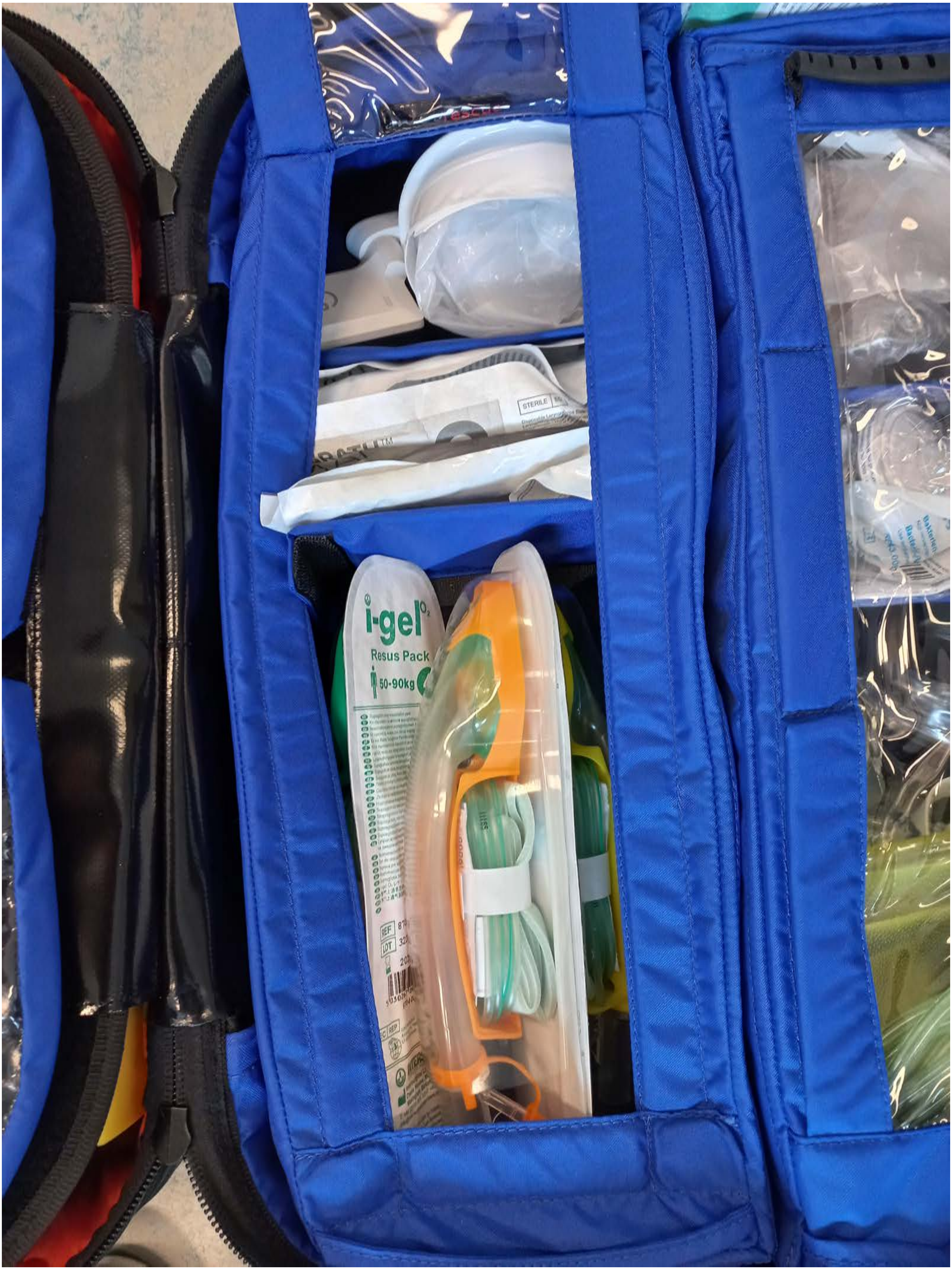
i-gel
Resus Pack
50-90kg
STERILE

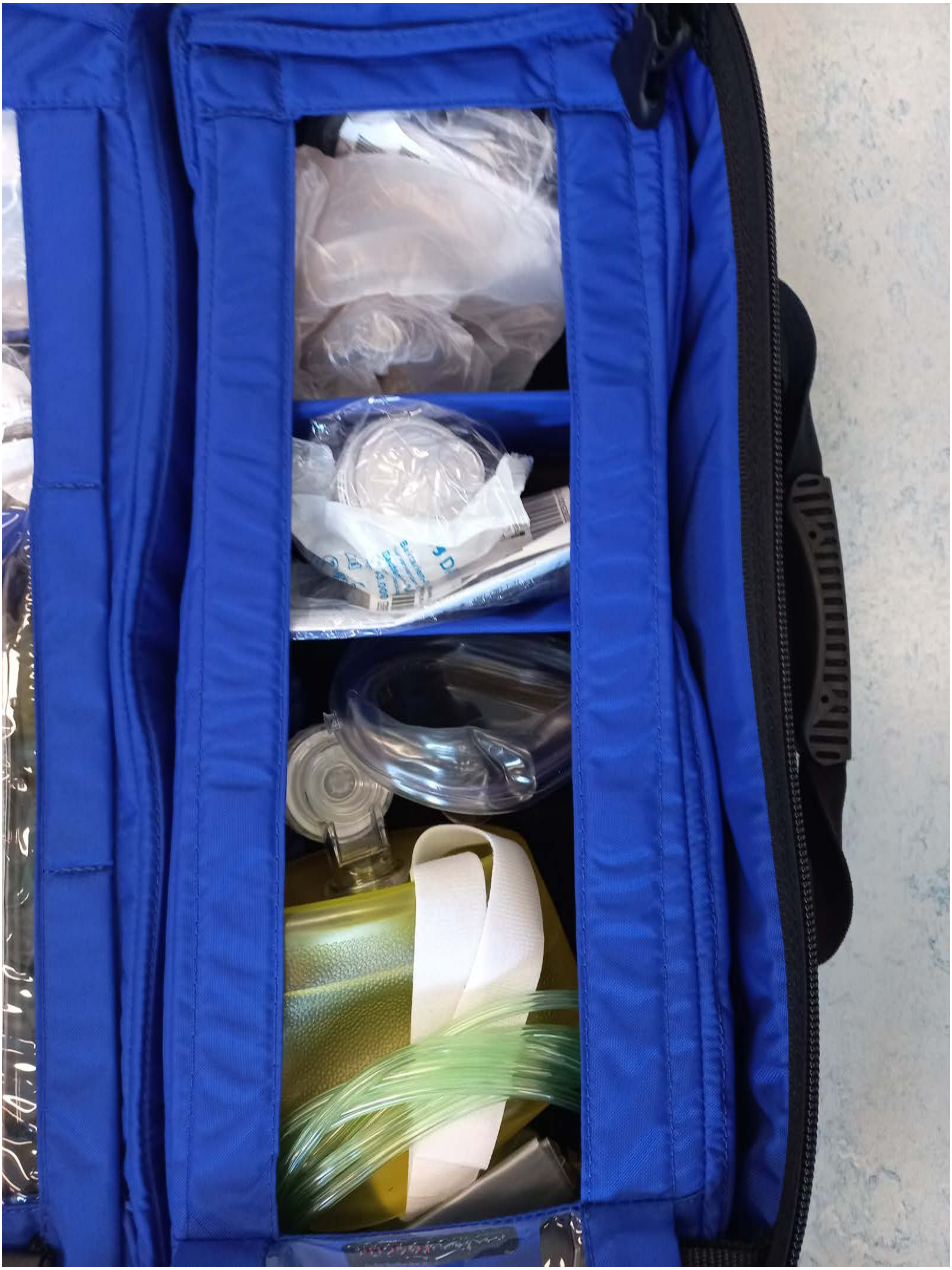

Supplement: Supplementary file 3 — Additional file3 (PDF 2841 kb) [file 13049_2024_1309_MOESM3_ESM.pdf]
