# Supplementary material for: Conventional versus task-based package organization for out-of-hospital emergency kits: an emergency medical services simulation study
Source: Scand J Trauma Resusc Emerg Med. 2024 Dec 20;32:135. doi: 10.1186/s13049-024-01309-8 (PMC11660938; doi:10.1186/s13049-024-01309-8)
Supplement: Supplementary file 4 — Additional file4 (PDF 2160 kb) [file 13049_2024_1309_MOESM4_ESM.pdf]

Existing Kit (modified to TPO)

Supraglottic Airway / Plan-B	i-gel laryngeal masks (various sizes)<br>lubricant
Airway Adjuncts	oropharyngeal Airways (various sizes)<br>nasopharyngeal airways (various sizes)<br>lubricant
BVM	self-inflating bag<br>oxygen tubing<br>oxygen resevoir<br>masks (various sizes)<br>PEEP valve<br>respiratory filter (various sizes)
Intubation	endotracheal tubes (various sizes)<br>lubricant<br>10ml syringe<br>endotracheal tube fixation material<br>magill forceps<br>stylet<br>manometer (for cuff)<br>extension tubing
Infusion	crystalloid infusion<br>glucose infusion<br>i.v. lines<br>3-way stopcock<br>spike adapter
Bandages	elastic bandage<br>scissors<br>self-adhesive bandage<br>plasters<br>various wound dressings
i.o. access	intraosseous drill<br>intraosseous needle-kits (various sizes)<br>3-way stopcock<br>flush-syringe
Video-Laryngoscope	video-laryngoscope<br>blades (various sizes)
Ampullarium	various medications<br>blunt needle

	Inset – see below<br>Paracetamol (100mL)<br>i.v. line<br>syringes (various sizes)
Ampullarium Inset	i.v.-catheters (various sizes)<br>3-way stopcocks<br>alcoholic swabs<br>dry gauze<br>i.v.-dressings<br>tourniquet<br>hypodermic needles<br>spike adapter<br>syringe caps<br>various non-i.v. medications
Diagnostics compartment	pen light<br>stethoscope<br>single-use clamp<br>pediatric-dosing aid<br>ear thermometer<br>blood-pressure cuff<br>glucose measurement set<br>antiseptic spray
Trauma compartment	foamed aluminium splint<br>pelvic binder<br>bleeding set – see below<br>bandages set – see below<br>tourniquets
Trauma Bleeding set	emergency bandage<br>hemostatic dressing<br>self-adhesive bandage
Trauma Bandages set	scissors<br>triangular bandages<br>emergency blankets<br>adhesive tape

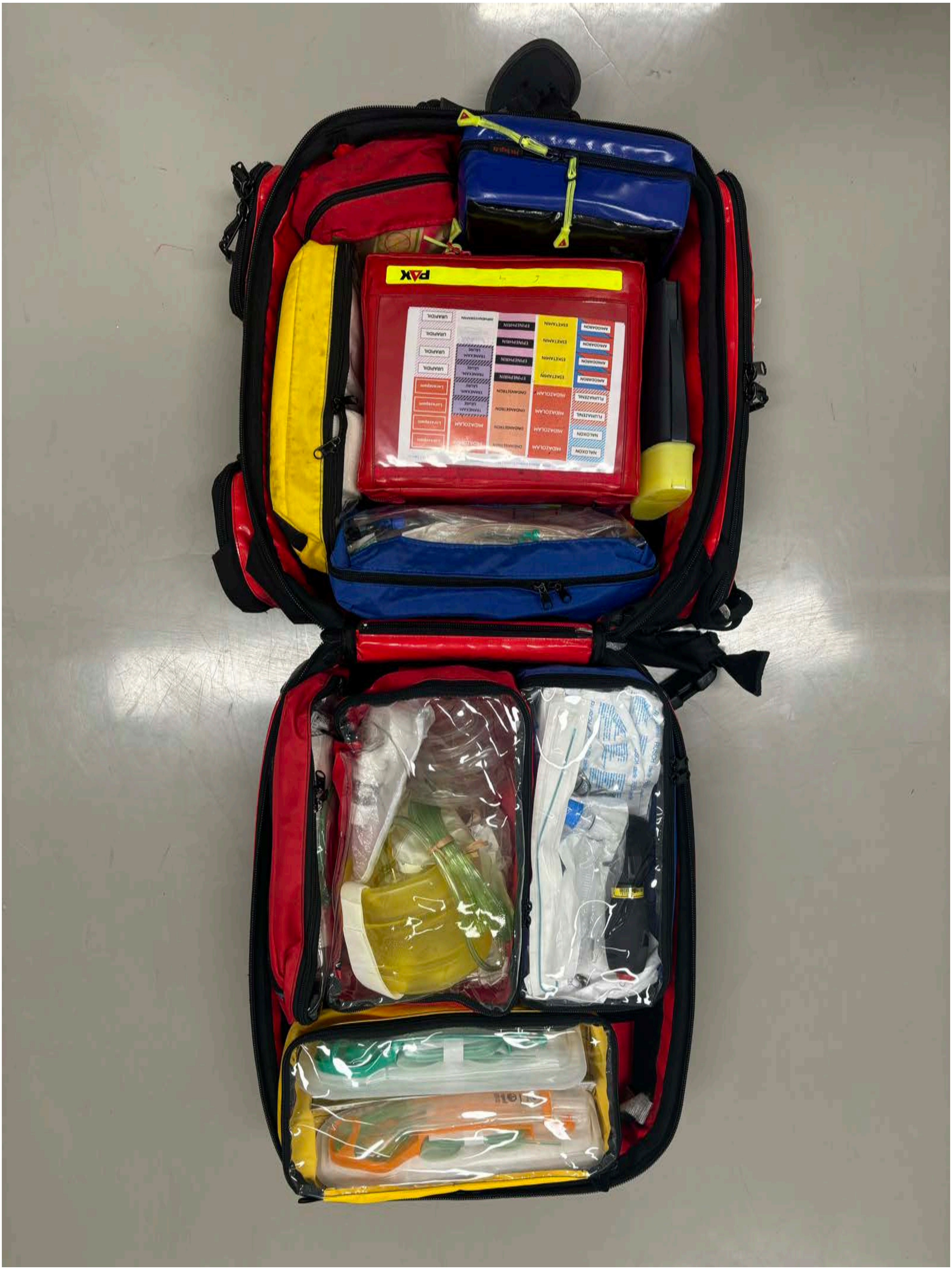
PAX
URAPIDIL
EPINEPHRIN
ESKETAMIN
AMIODARON
TRANEXAM
ONDANSETRON
MIDAZOLAM
FLUMAZENIL
NALOXON

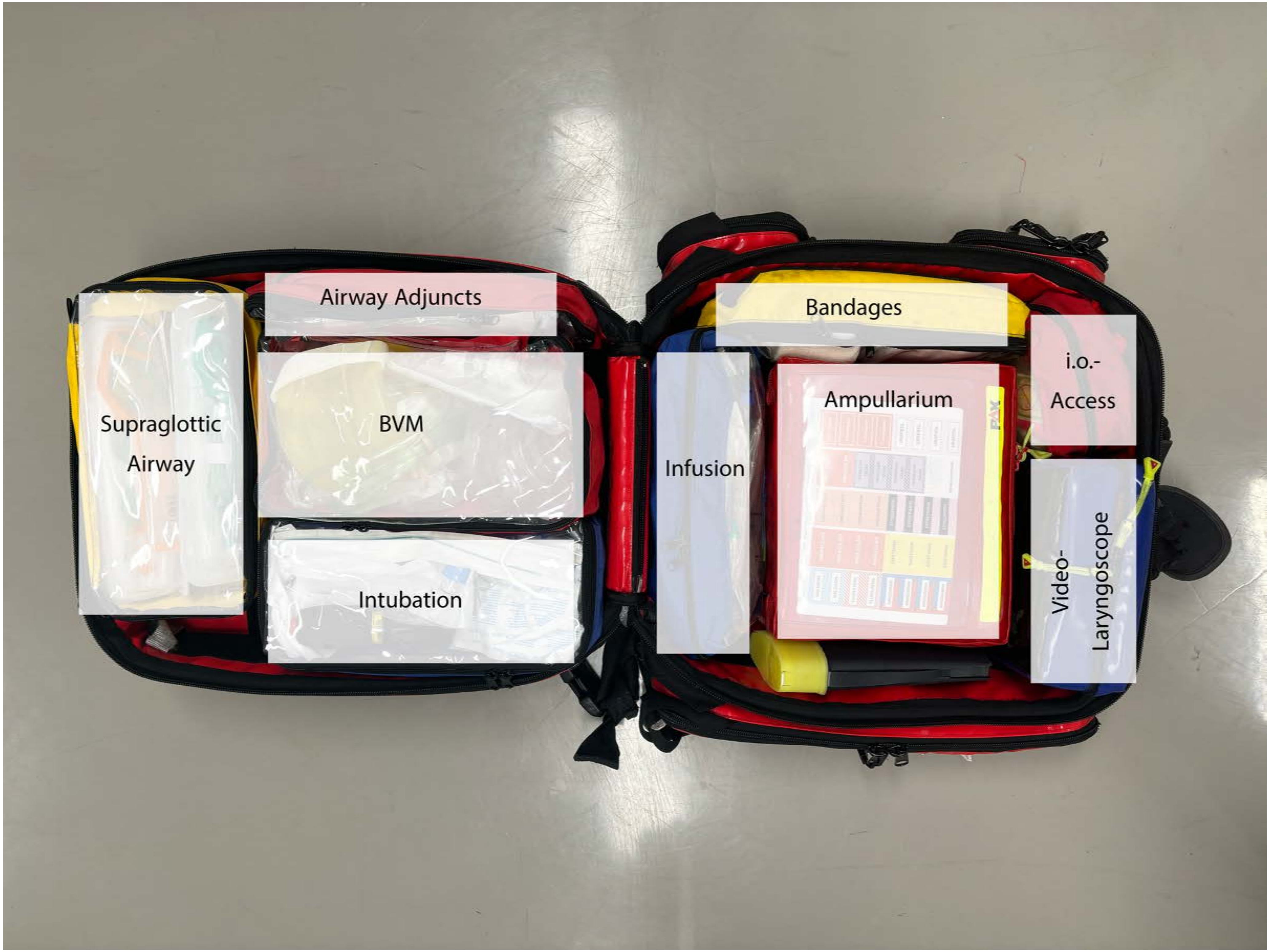

Airway Adjuncts
Bandages
Supraglottic
Airway
BVM
i.o.-
Access
Ampullarium
Infusion
Intubation
Video-
Laryngoscope

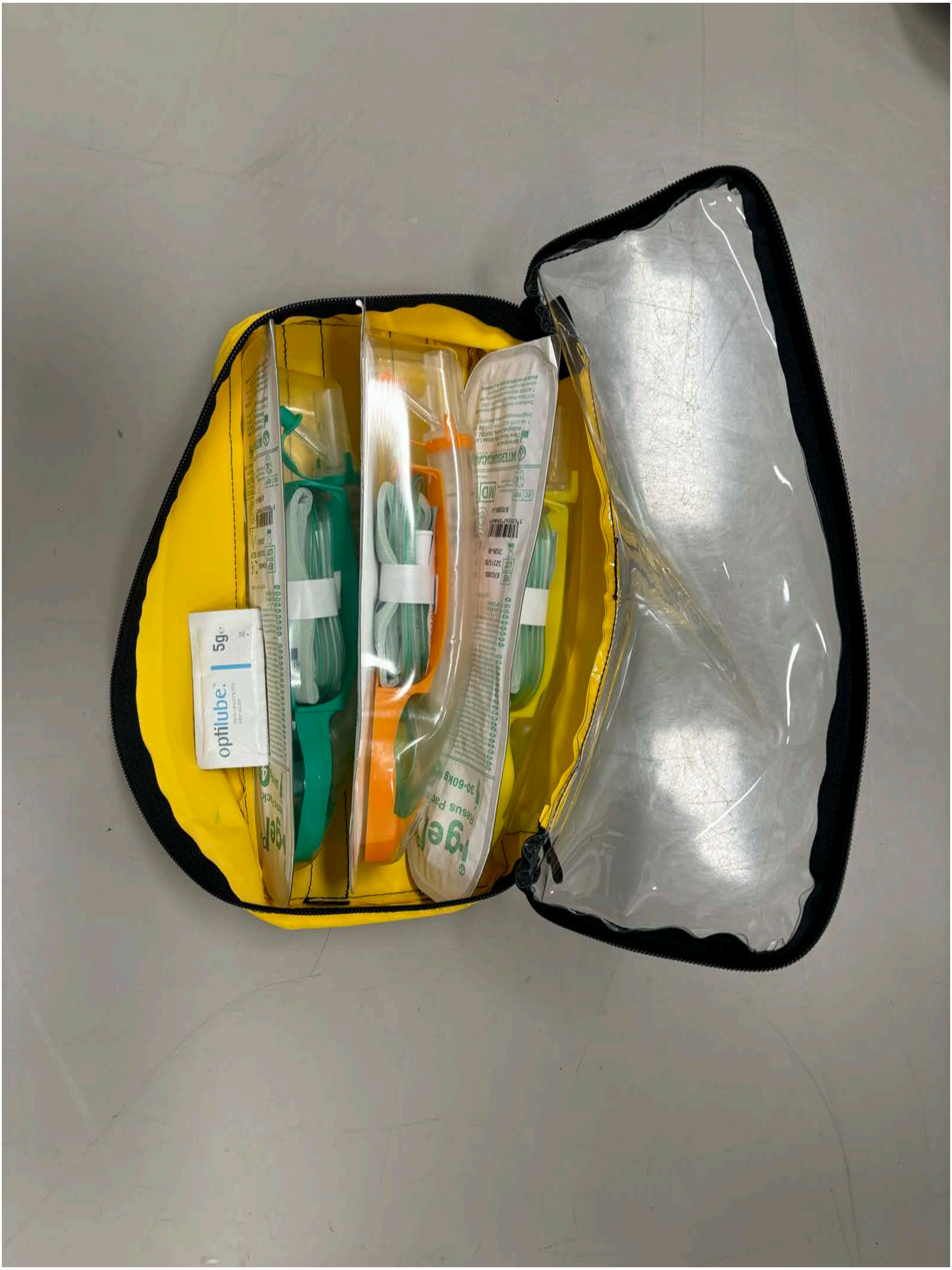
optilube
5g
i-gel
Resus Pac
30-60kg

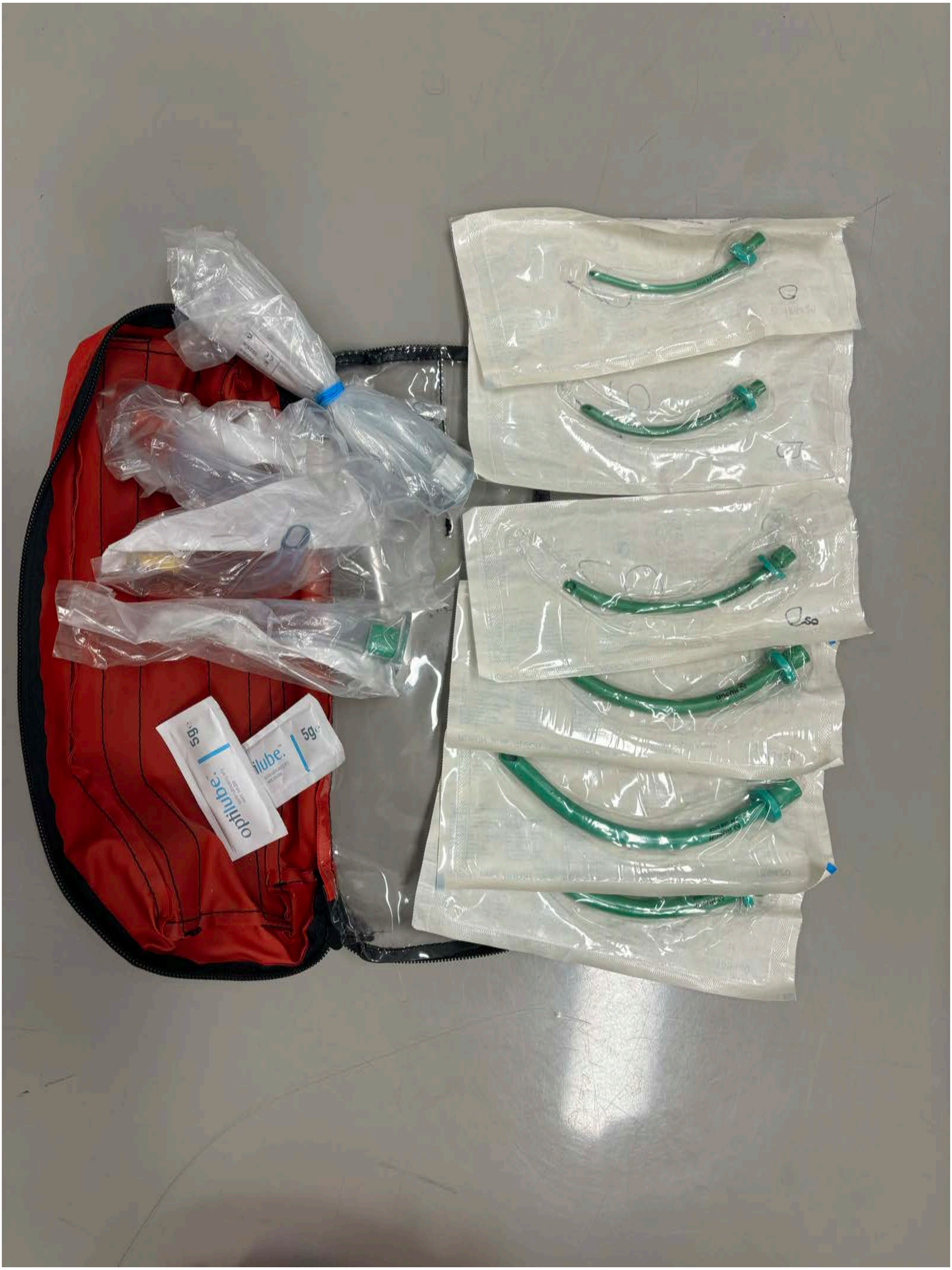

5g
optilube.
5g

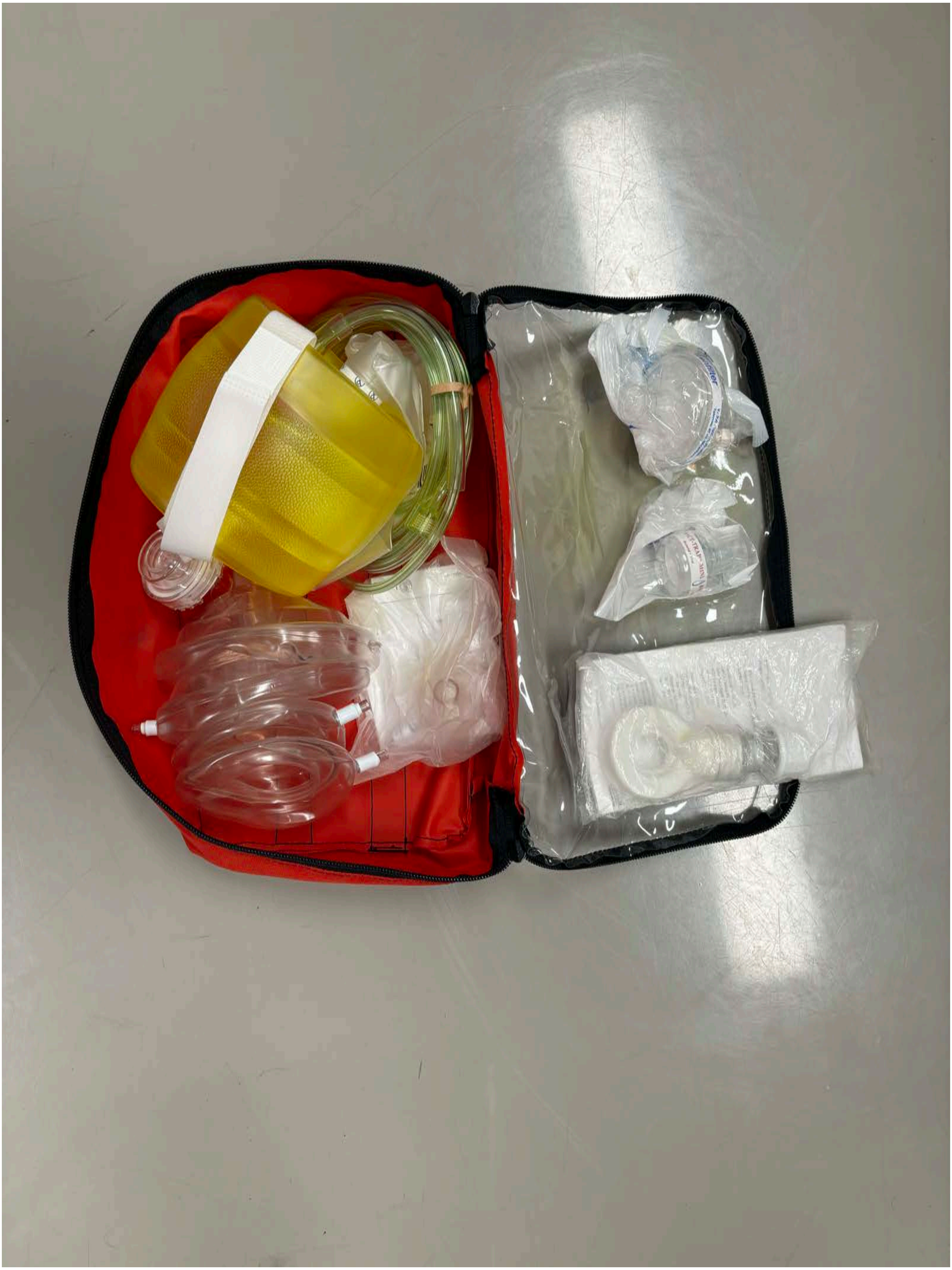

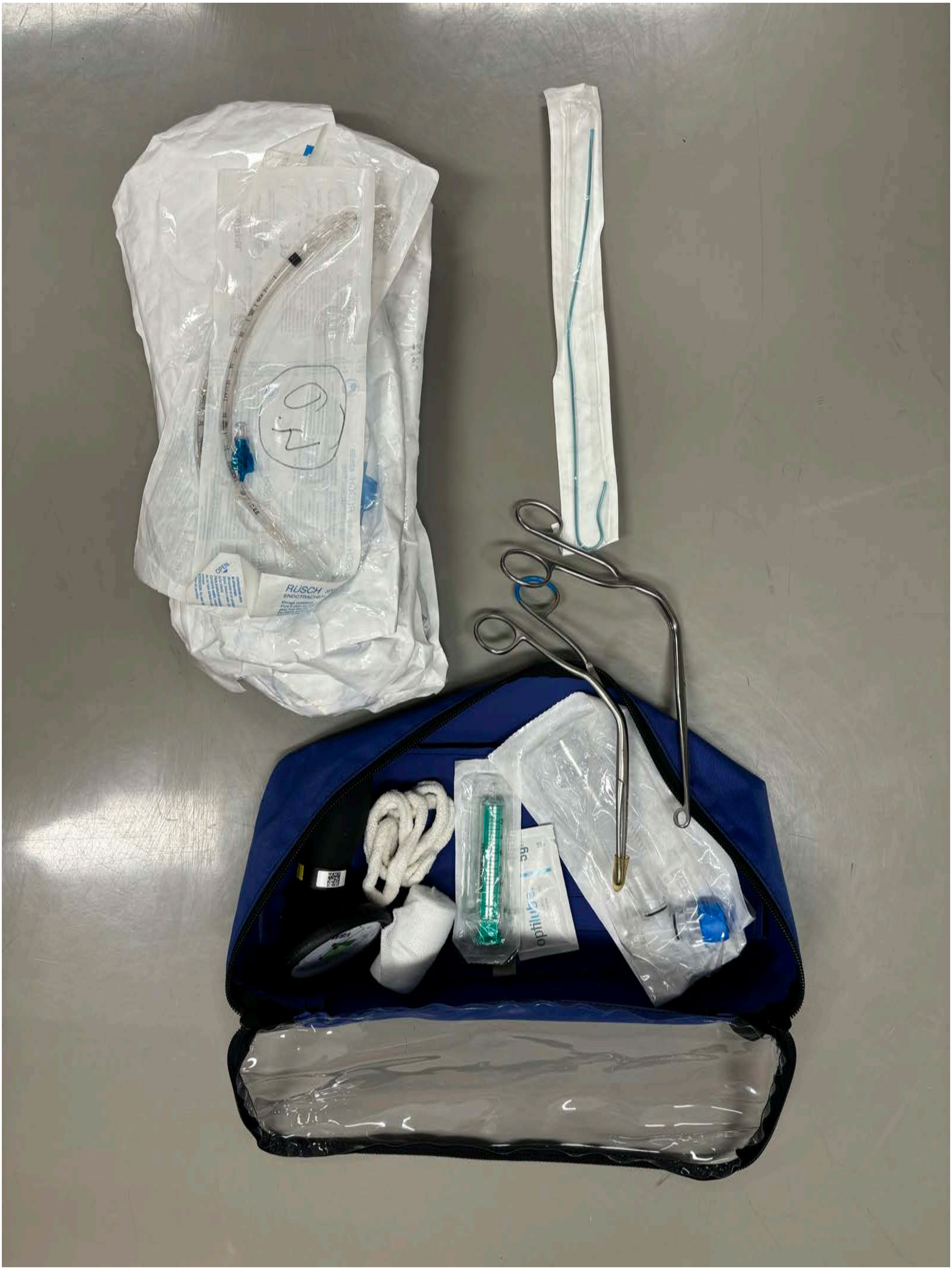
RUSCH

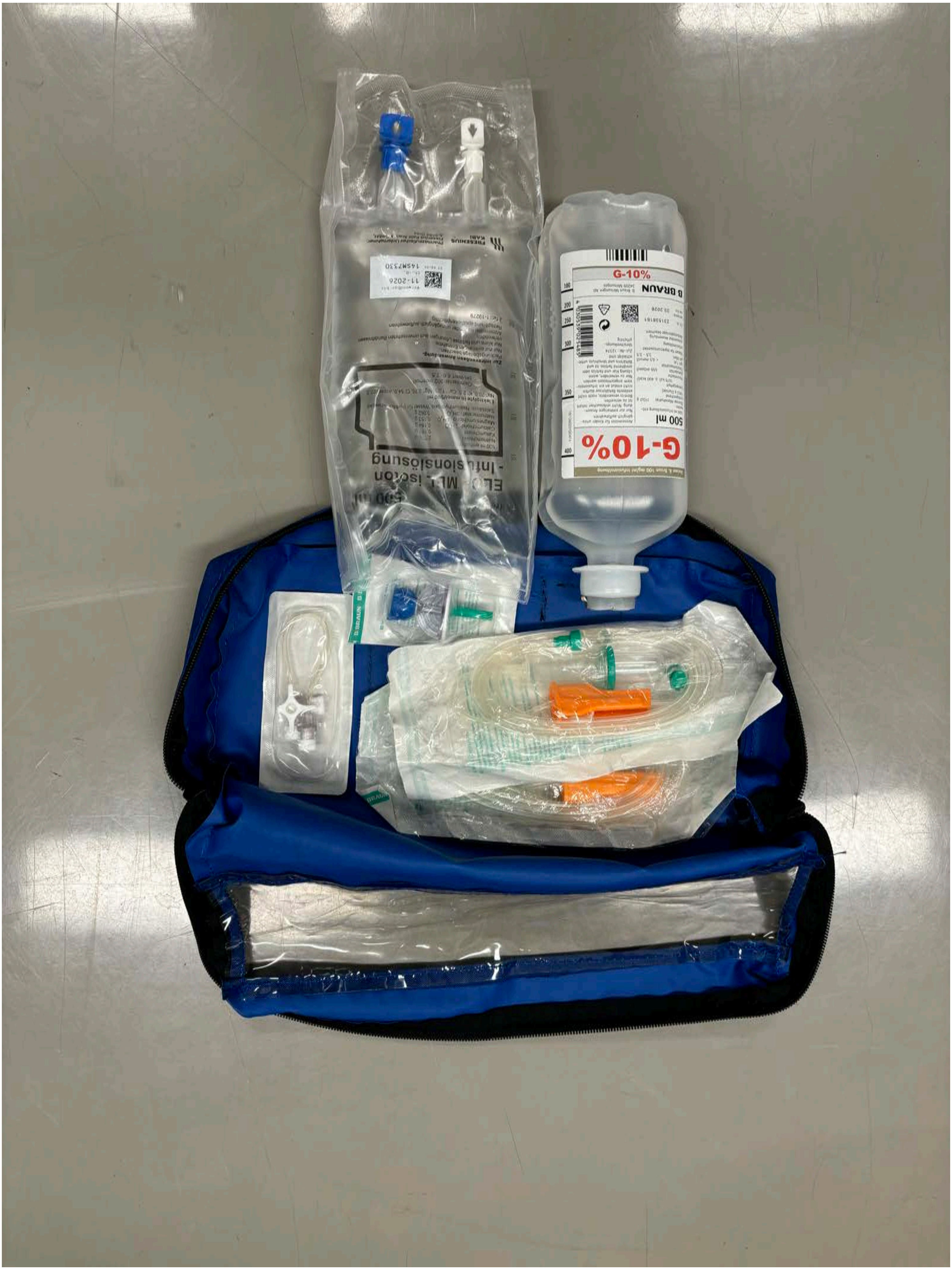
G-10%
B.BRAUN
500 ml
G-10%

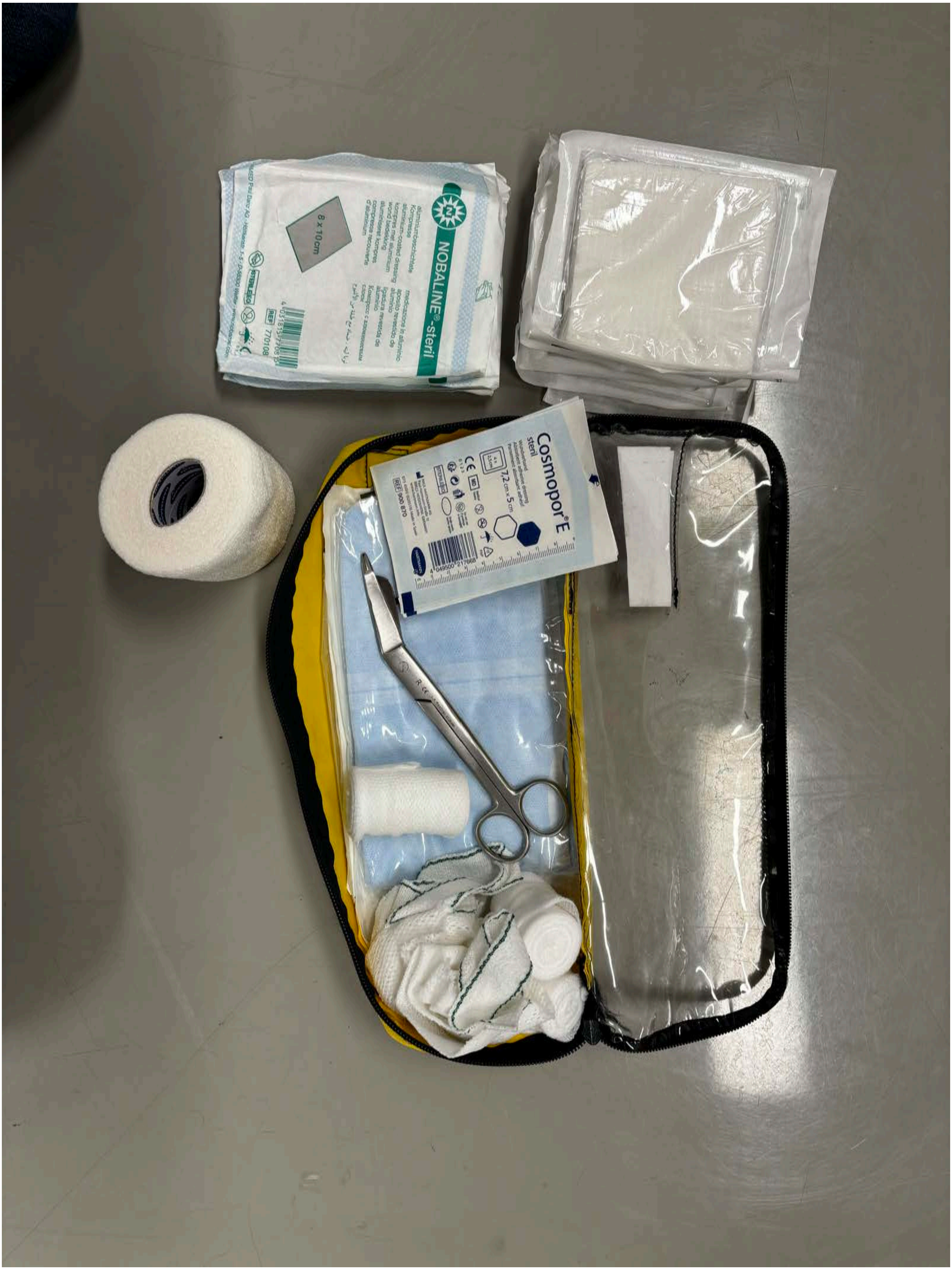
NOBALINE®-steril
8 x 10 cm
REF 770108
Cosmopor® E
steril
7,2 cm x 5 cm
REF 900 870

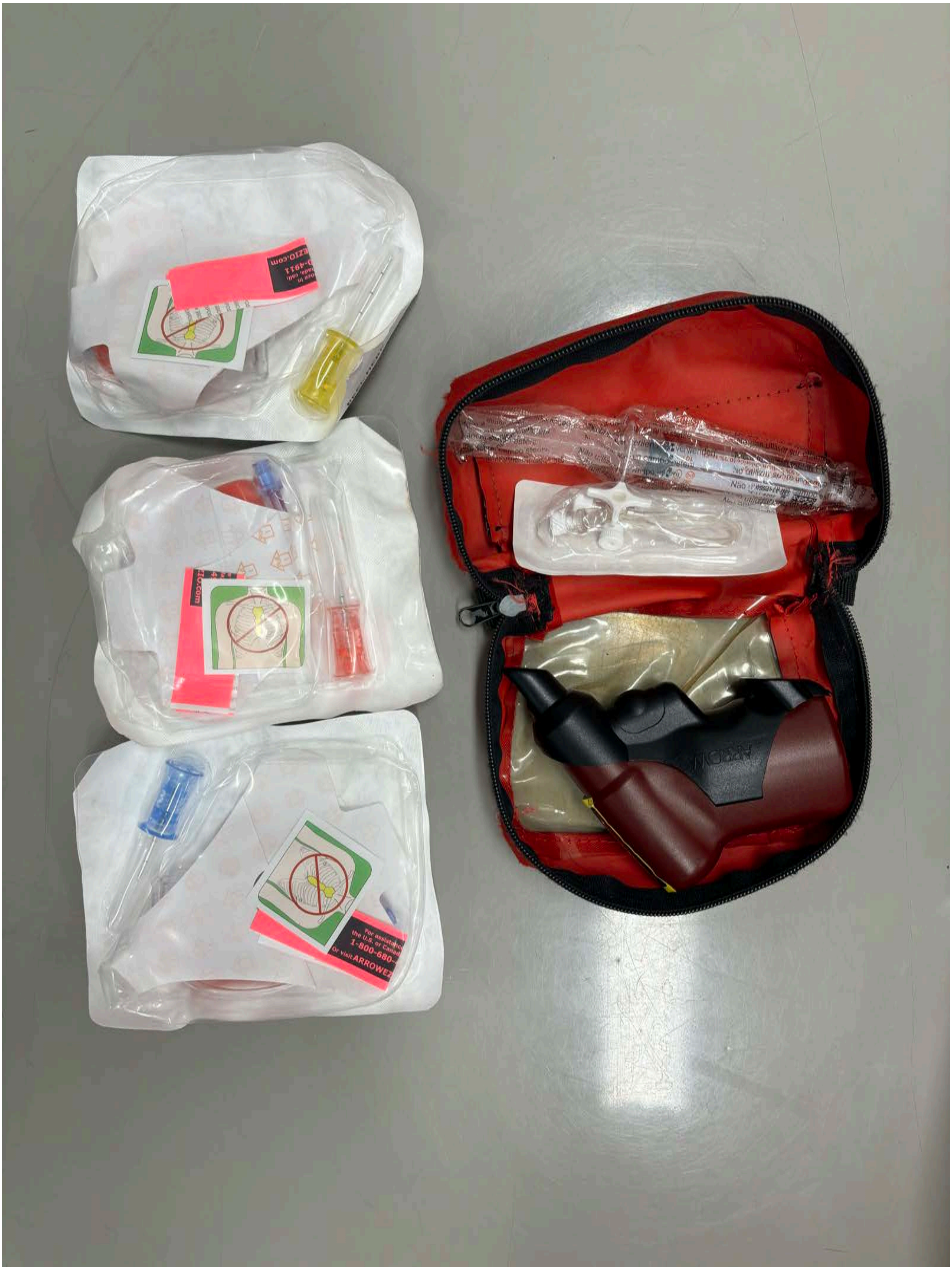

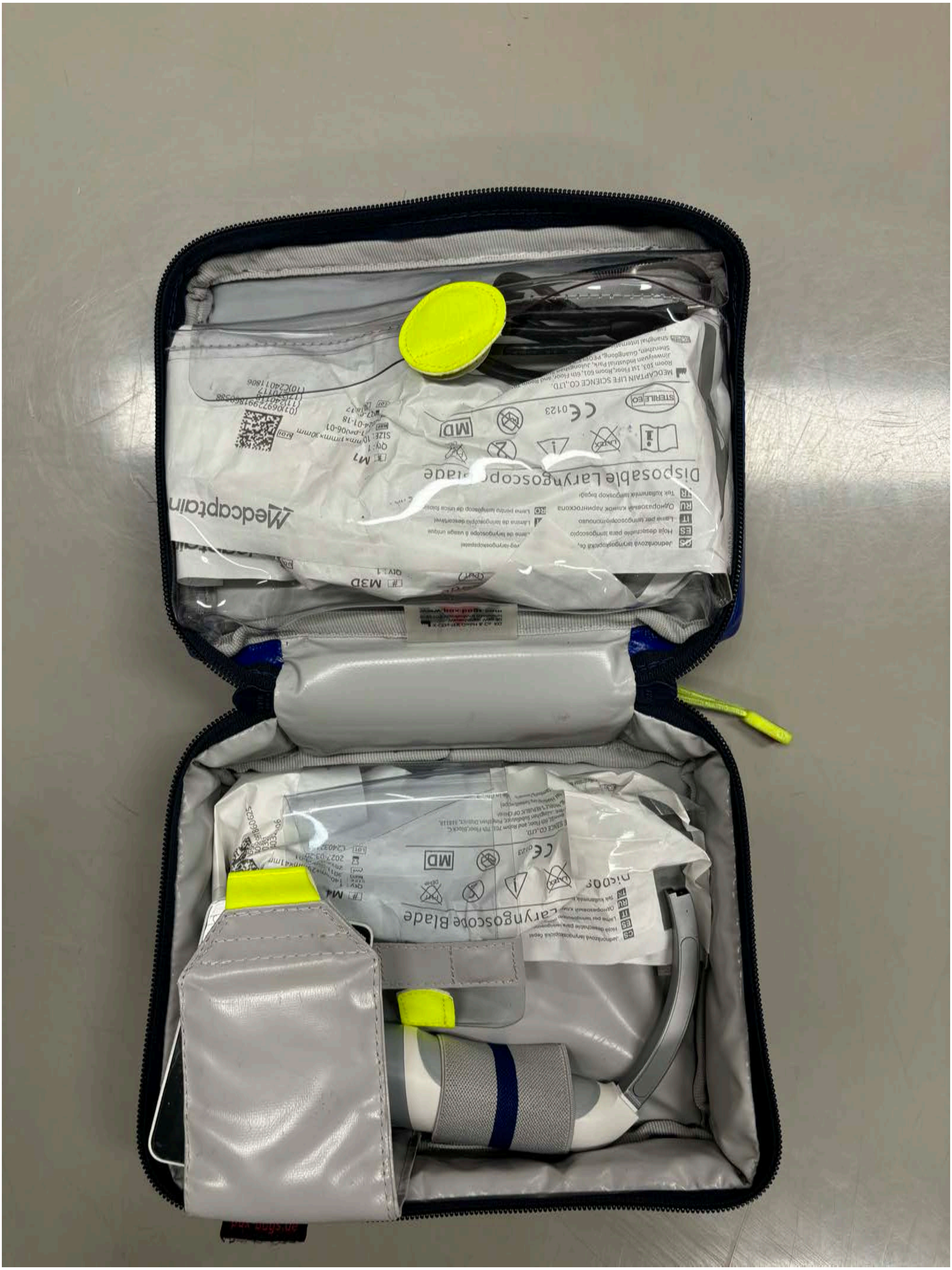
Disposable Laryngoscope Blade
Medcaptain
MEDCAPTAIN LIFE SCIENCE CO.,LTD.
Laryngoscope Blade

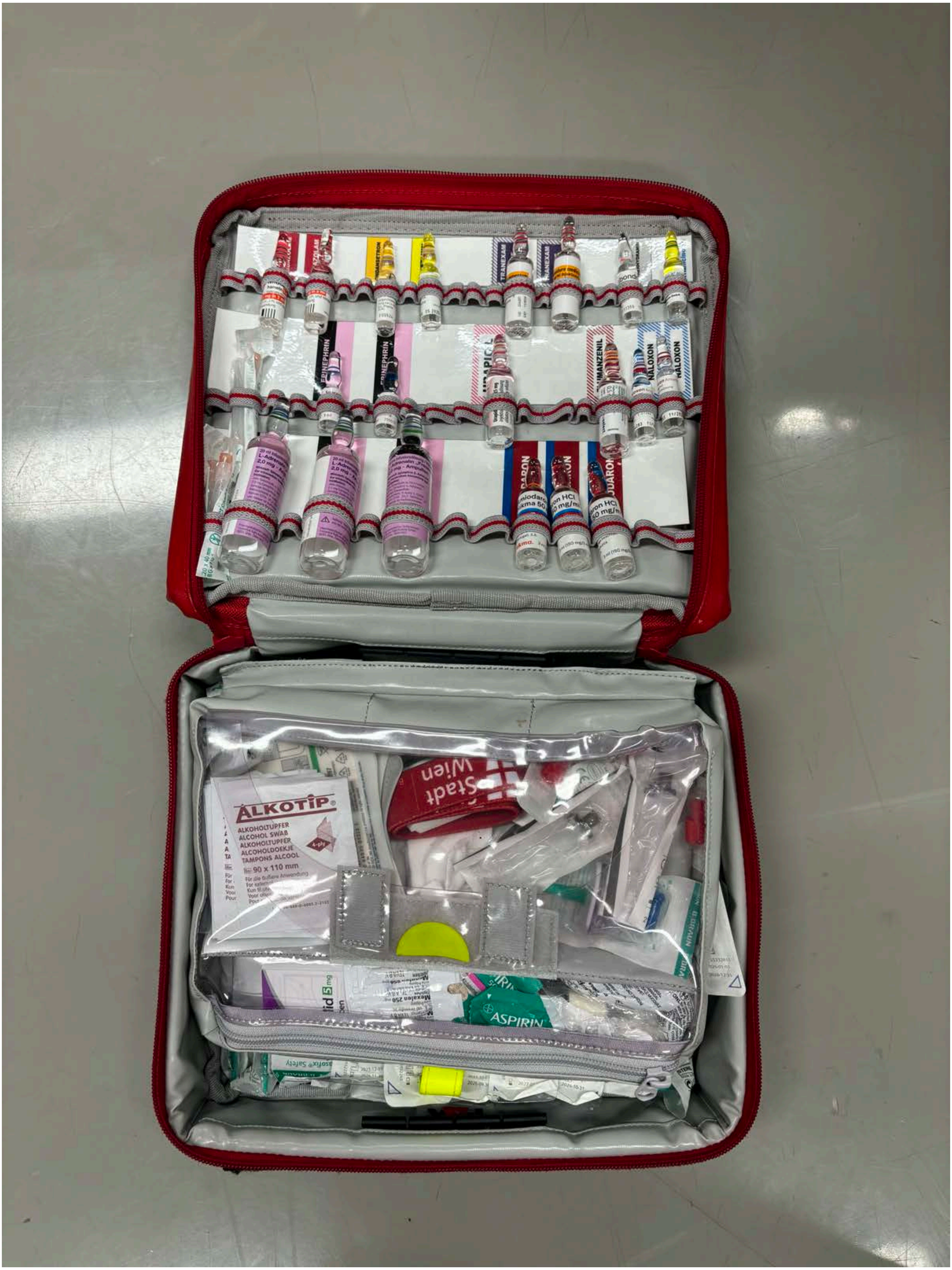
TRANEXAM
TRANEXAM
EPINEPHRIN
EPINEPHRIN
NALOXON
NALOXON
ALKOTIP®
ALKOHOLTUPFER
ALCOHOL SWAB
ALKOHOLTUPFER
ALCOHOLDOEKJE
TAMPONS ALCOOL
90 x 110 mm
Stadt Wien
ASPIRIN
Mexalen 250

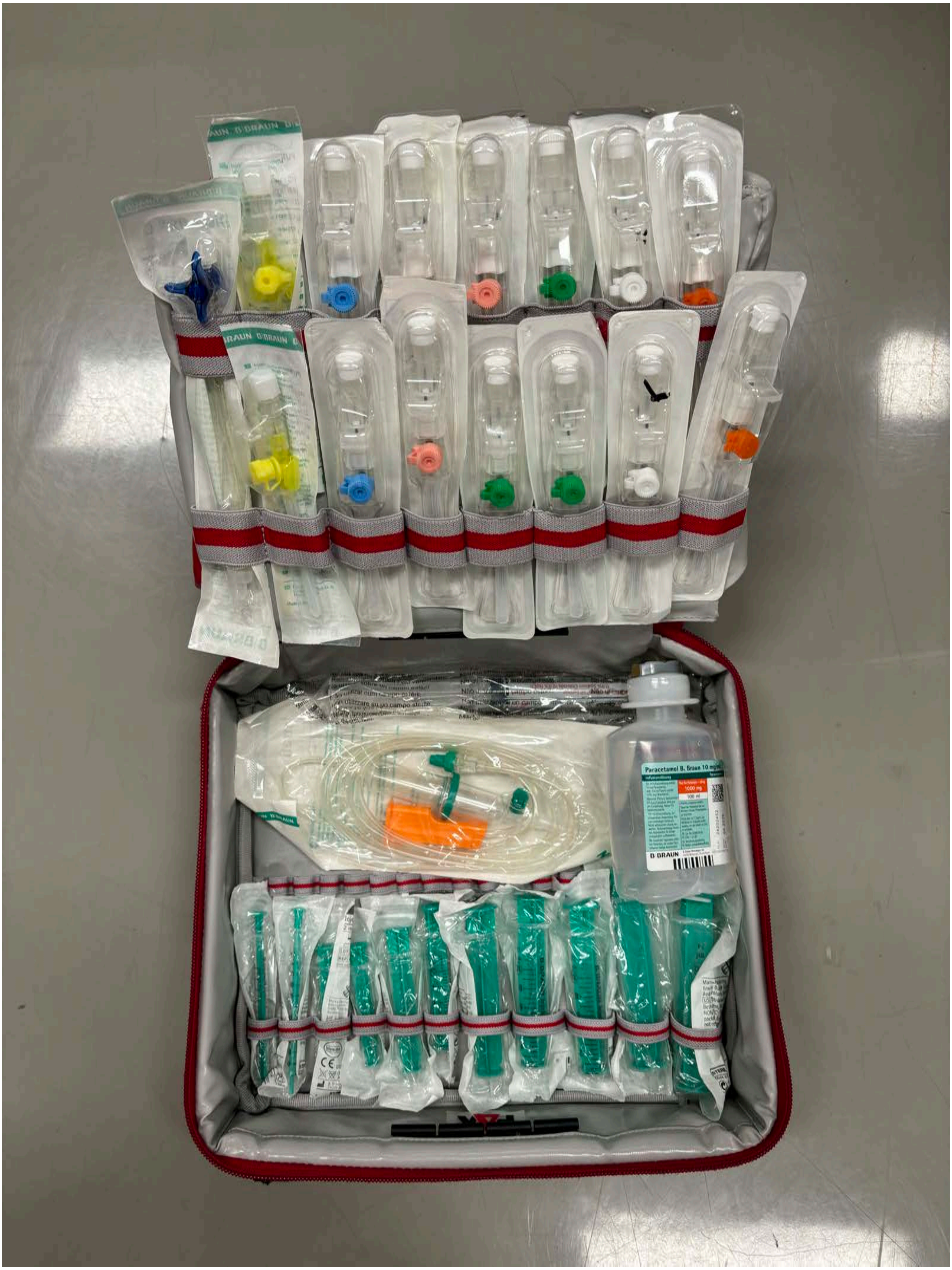
1000 mg
100 ml
B BRAUN

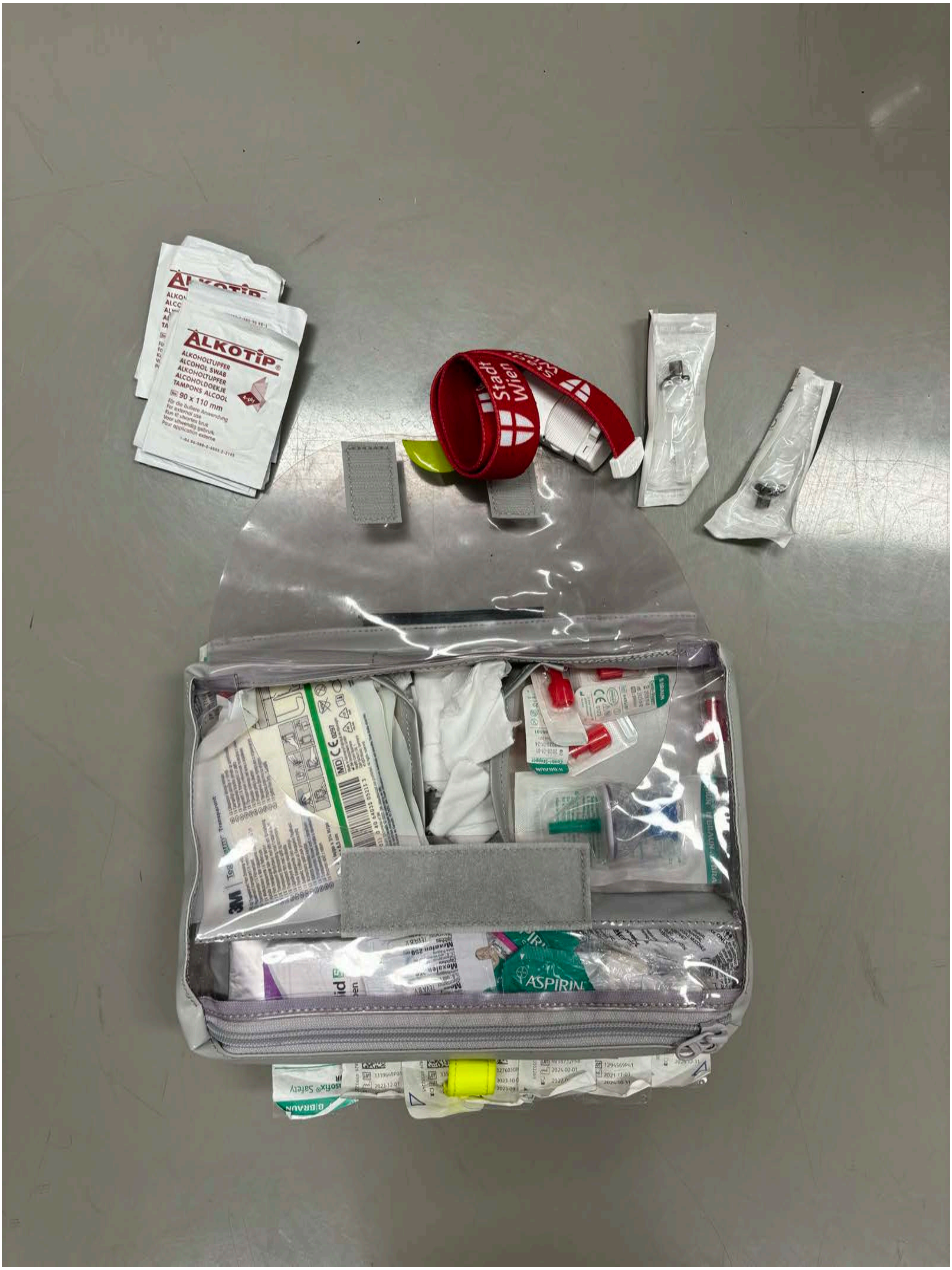
ALKOTIP
ALKOHOLTUPFER
ALCOHOL SWAB
ALCOHOLTUPFER
ALCOHOLDOEKJE
TAMPONS ALCOOL
90 x 110 mm
Stadt Wien
ASPIRIN
B|BRAUN

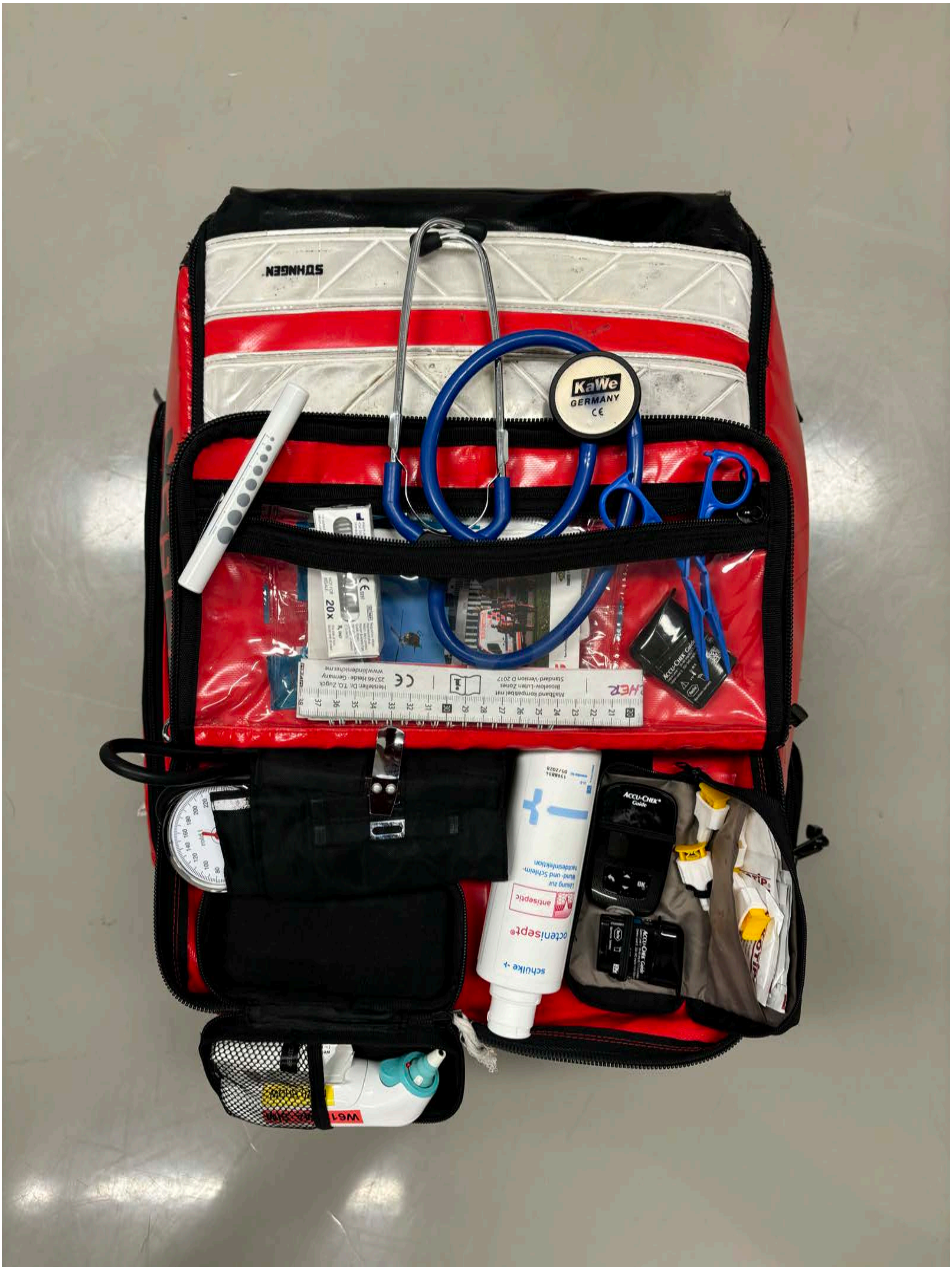

KaWe
GERMANY
ACCU-CHEK Guide
octenisept
schülke
antiseptic

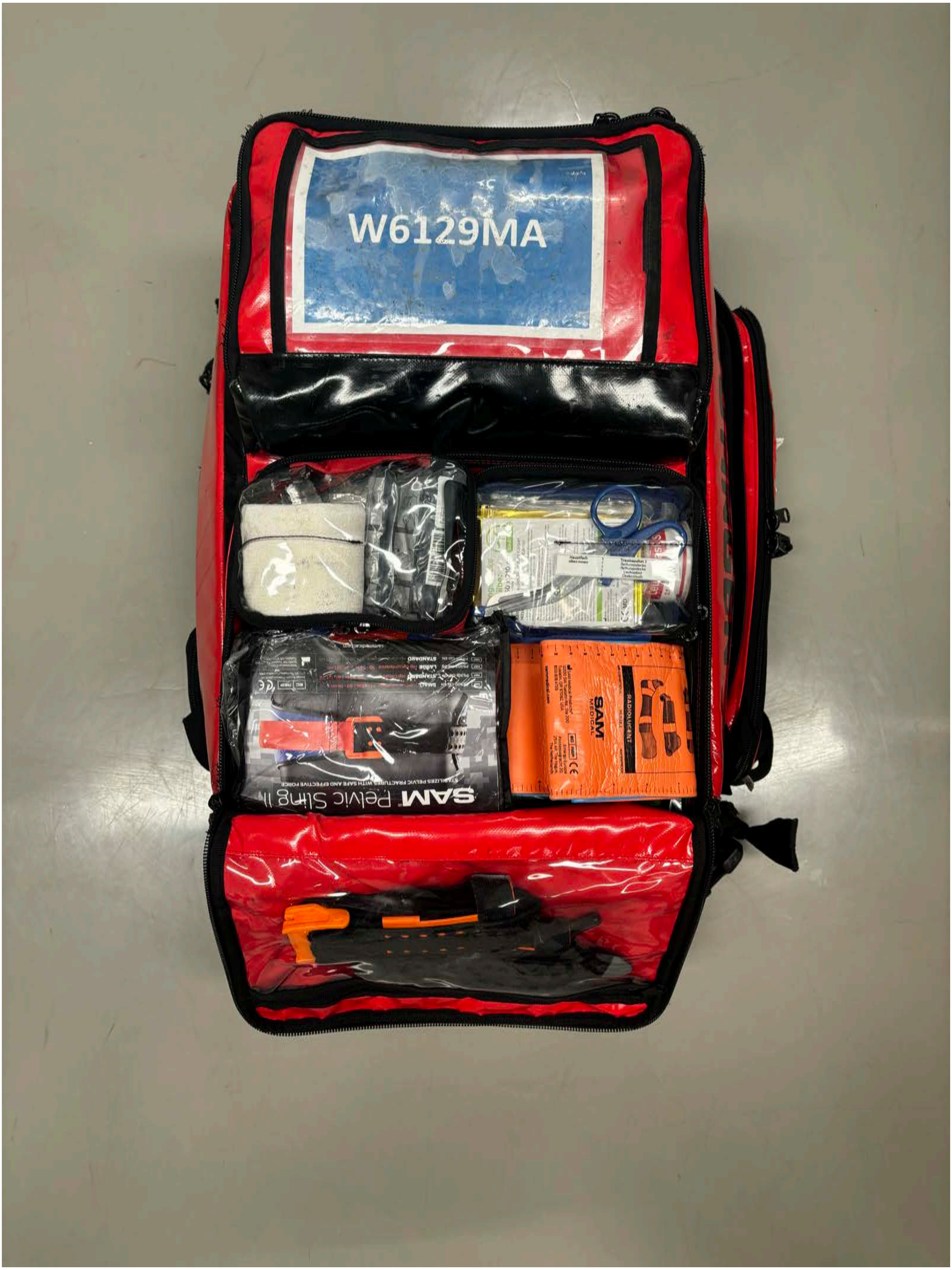
W6129MA
SAM Pelvic Sling II

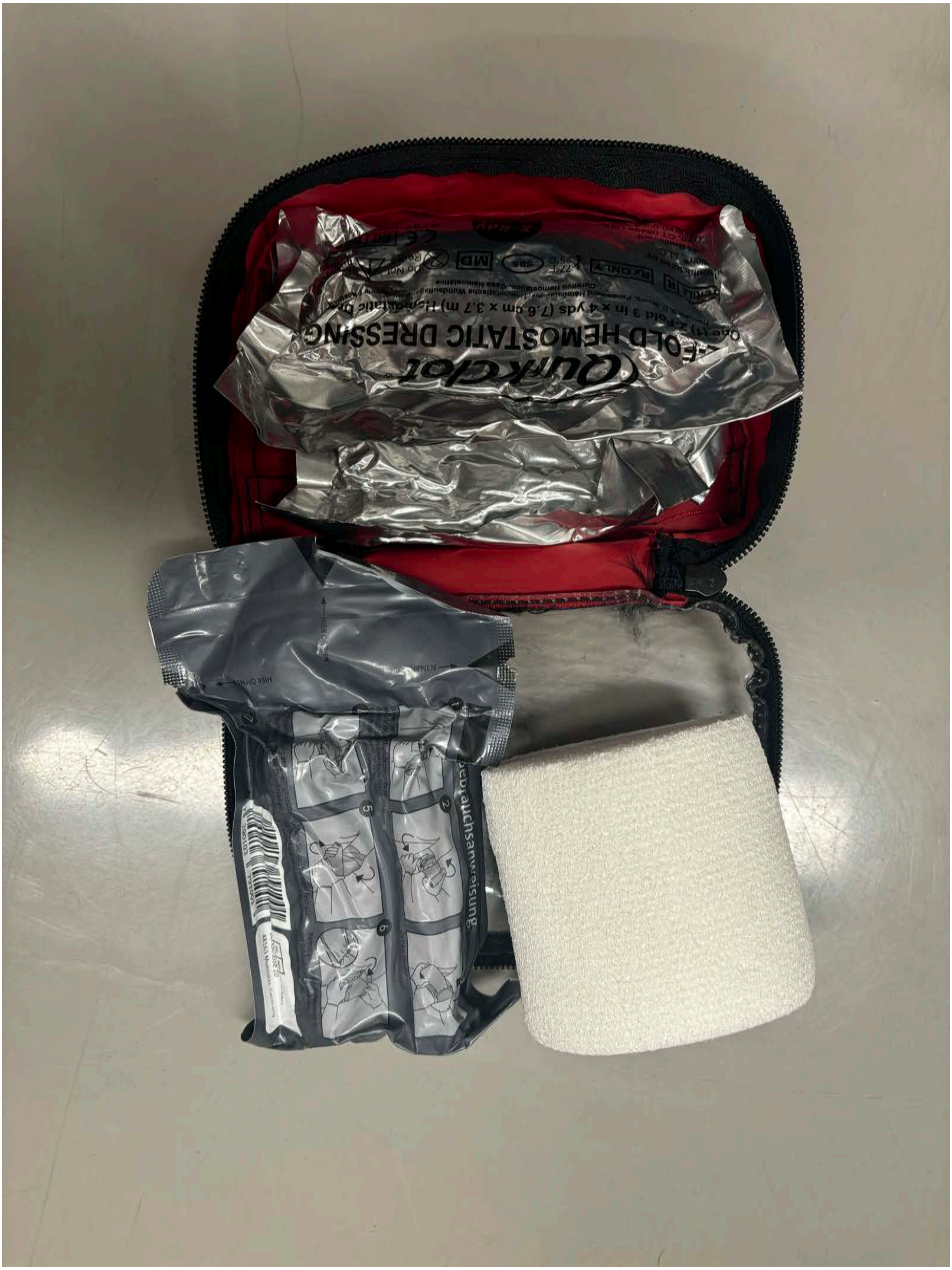
QuikClot
FOLD HEMOSTATIC DRESSING
Gebrauchsanweisung

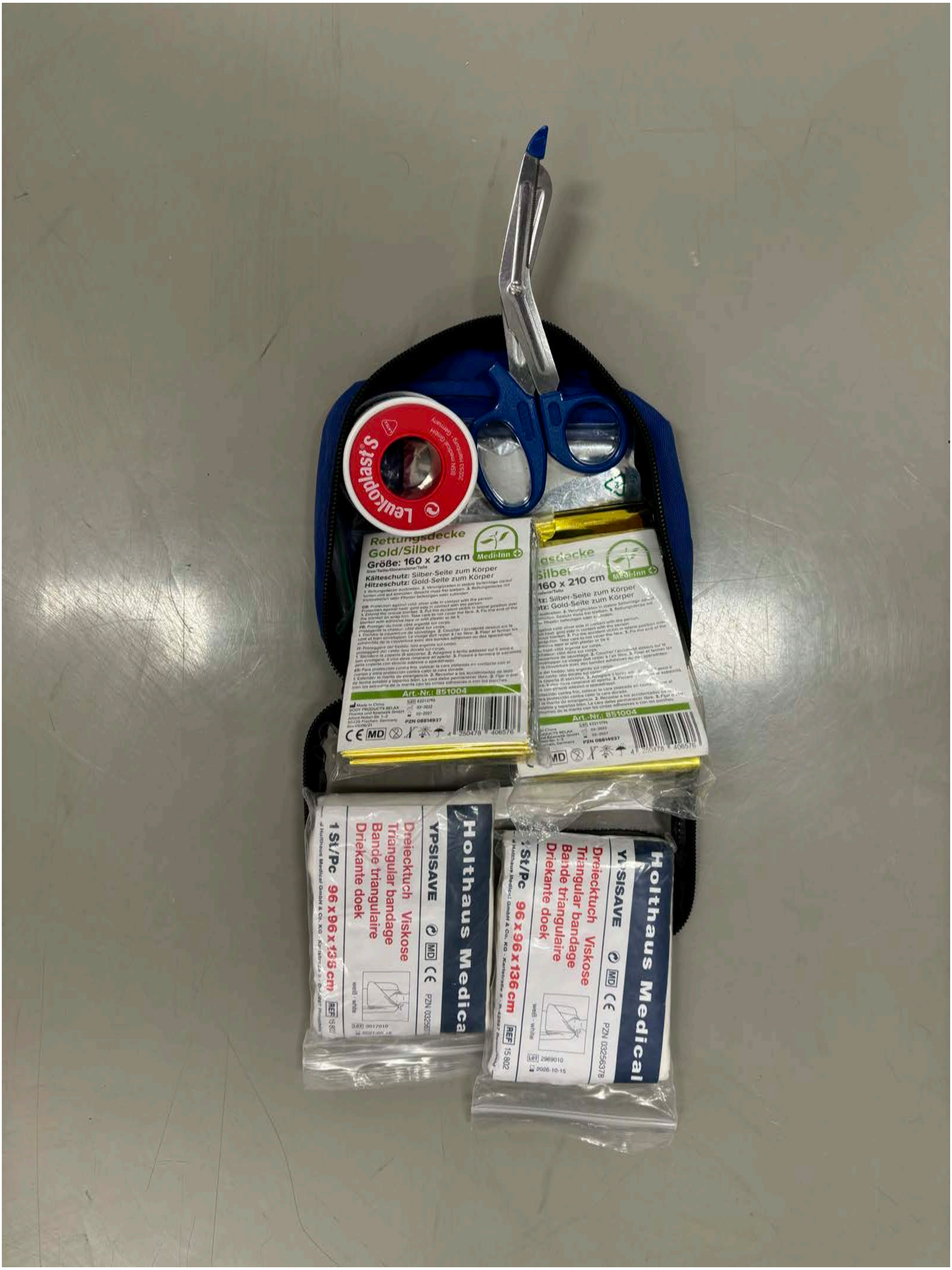
Leukoplast S
Rettungsdecke
Gold/Silber
Größe: 160 x 210 cm
Medi-Inn
Kälteschutz: Silber-Seite zum Körper
Hitzeschutz: Gold-Seite zum Körper
Art.-Nr.: 851004
Holthaus Medical
YPSISAVE
Dreiecktuch Viskose
Triangular bandage
Bande triangulaire
Driekante doek
1 St/Pc 96 x 96 x 136 cm
PZN 03256378
REF 15.802
