# Supplementary material for: Conventional versus task-based package organization for out-of-hospital emergency kits: an emergency medical services simulation study
Source: Scand J Trauma Resusc Emerg Med. 2024 Dec 20;32:135. doi: 10.1186/s13049-024-01309-8 (PMC11660938; doi:10.1186/s13049-024-01309-8)
Supplement: Supplementary file 5 — Additional file5 (PDF 2314 kb) [file 13049_2024_1309_MOESM5_ESM.pdf]

## Existing kit (non-TPO)

| | |
|---|---|
| Supraglottic Airway / Plan-B | i-gel laryngeal masks (various sizes)<br>lubricant |
| Video-Laryngoscope | video-laryngoscope<br>blades (various sizes) |
| Bleeding | emergency bandage<br>hemostatic dressing |
| Intubation | endotracheal tubes (various sizes)<br>lubricant<br>10ml syringe<br>endotracheal tube fixation material<br>magill forceps<br>stylet<br>extension tubing<br>laryngoscope handle<br>laryngoscope blades (various sizes) |
| Minor Trauma | triangular bandages<br>emergency blankets<br>adhesive tape |
| Bandages | elastic bandage<br>scissors<br>self-adhesive bandage<br>plasters<br>various wound dressings |
| i.o. access | intraosseous drill<br>intraosseous needle-kits (various sizes)<br>3-way stopcock<br>flush-syringe |
| Ampullarium | various medications<br>blunt needle<br>Inset – see below<br>Paracetamol (100mL)<br>crystalloid infusion<br>glucose infusion<br>i.v. lines<br>syringes (various sizes) |
| Ampullarium Inset | i.v.-catheters (various sizes) |

| | |
|---|---|
| | 3-way stopcocks |
| | alcoholic swabs |
| | dry gauze |
| | i.v.-dressings |
| | tourniquet |
| | hypodermic needles |
| | spike adapter |
| | syringe caps |
| | various non-i.v. medications |
| BVM | self-inflating bag |
| | oxygen tubing |
| | oxygen resevoir |
| | masks (various sizes) |
| | PEEP valve |
| | respiratory filter (various sizes) |
| Airway Adjuncts | oropharyngeal Airways (various sizes) |
| | nasopharyngeal airways (various sizes) |
| | lubricant |
| Pelvic binder | |
| Foamed aluminum splint | |
| Diagnostics compartment | pen light |
| | stethoscope |
| | single-use clamp |
| | pediatric-dosing aid |
| | ear thermometer |
| | blood-pressure cuff |
| | glucose measurement set |
| | antiseptic spray |
| | scissors |
| Large front compartment | tourniquets |
| | (free space was used for Covid-19 PPE) |

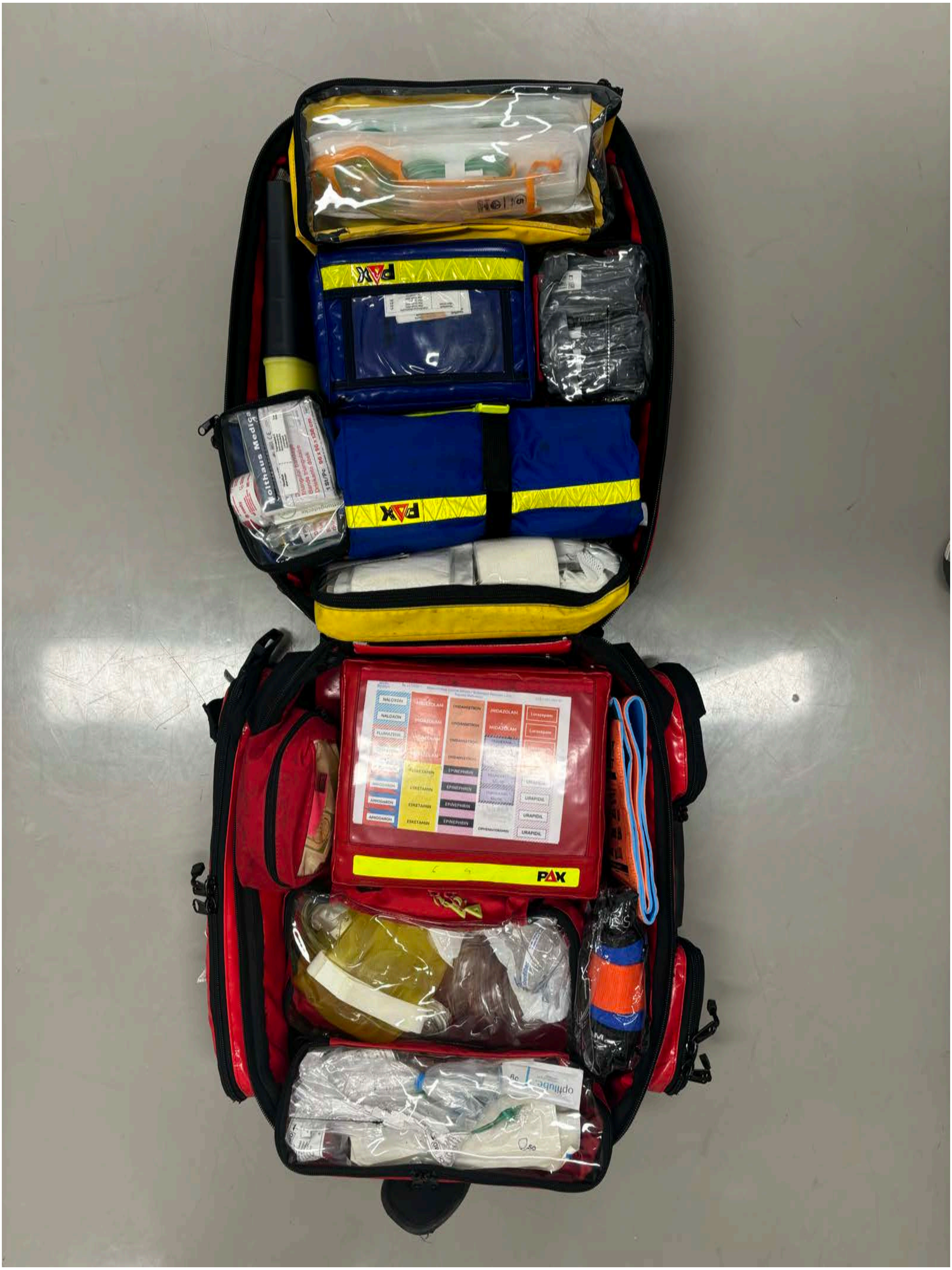
PAX
PAX
Holthaus Medical
NALOXON
MIDAZOLAM
ONDANSETRON
ESKETAMIN
EPINEPHRIN
URAPIDIL
AMIODARON
PAX

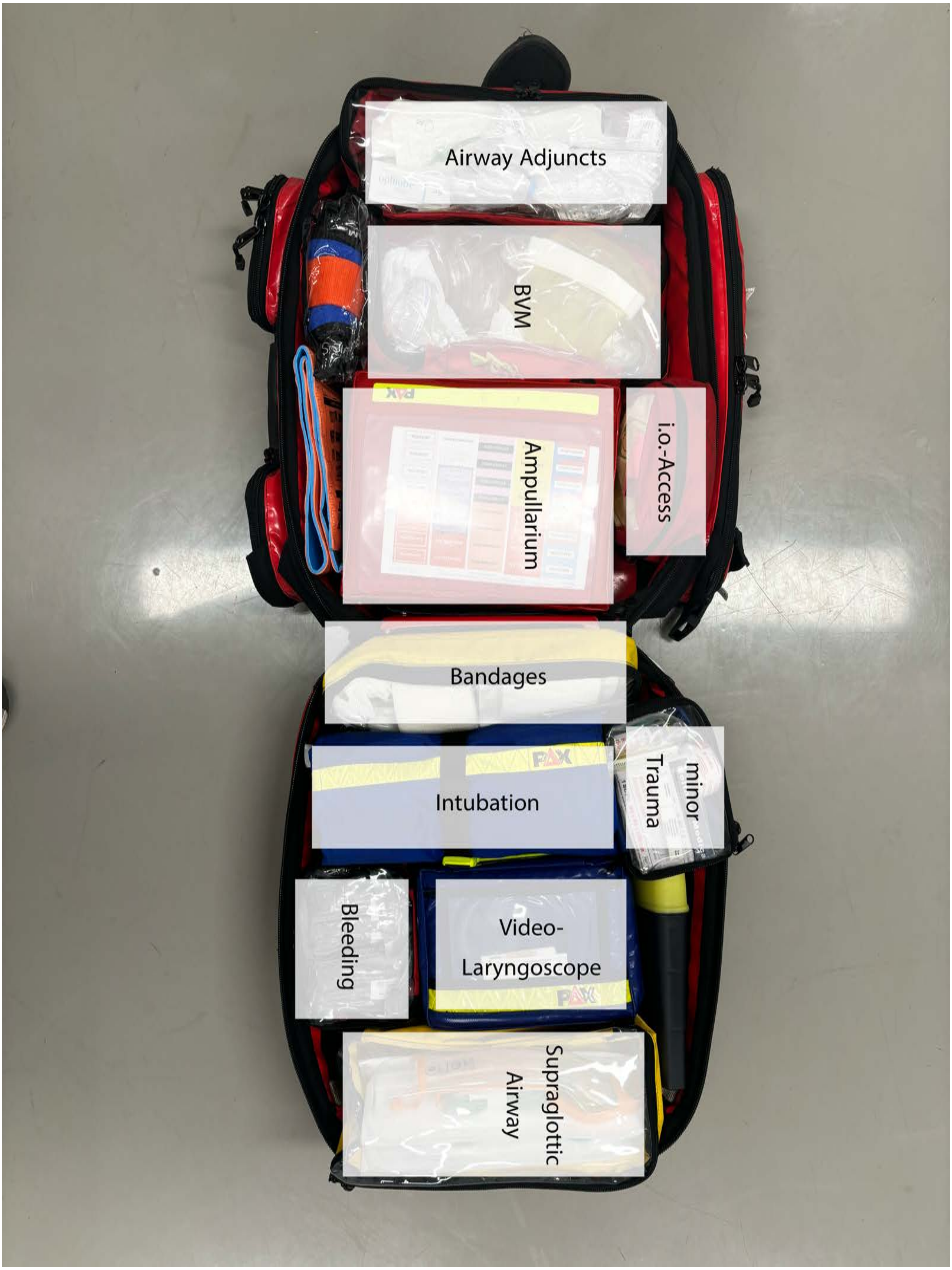
Airway Adjuncts
BVM
Ampullarium
i.o.-Access
Bandages
Intubation
minor Trauma
Bleeding
Video-Laryngoscope
Supraglottic Airway

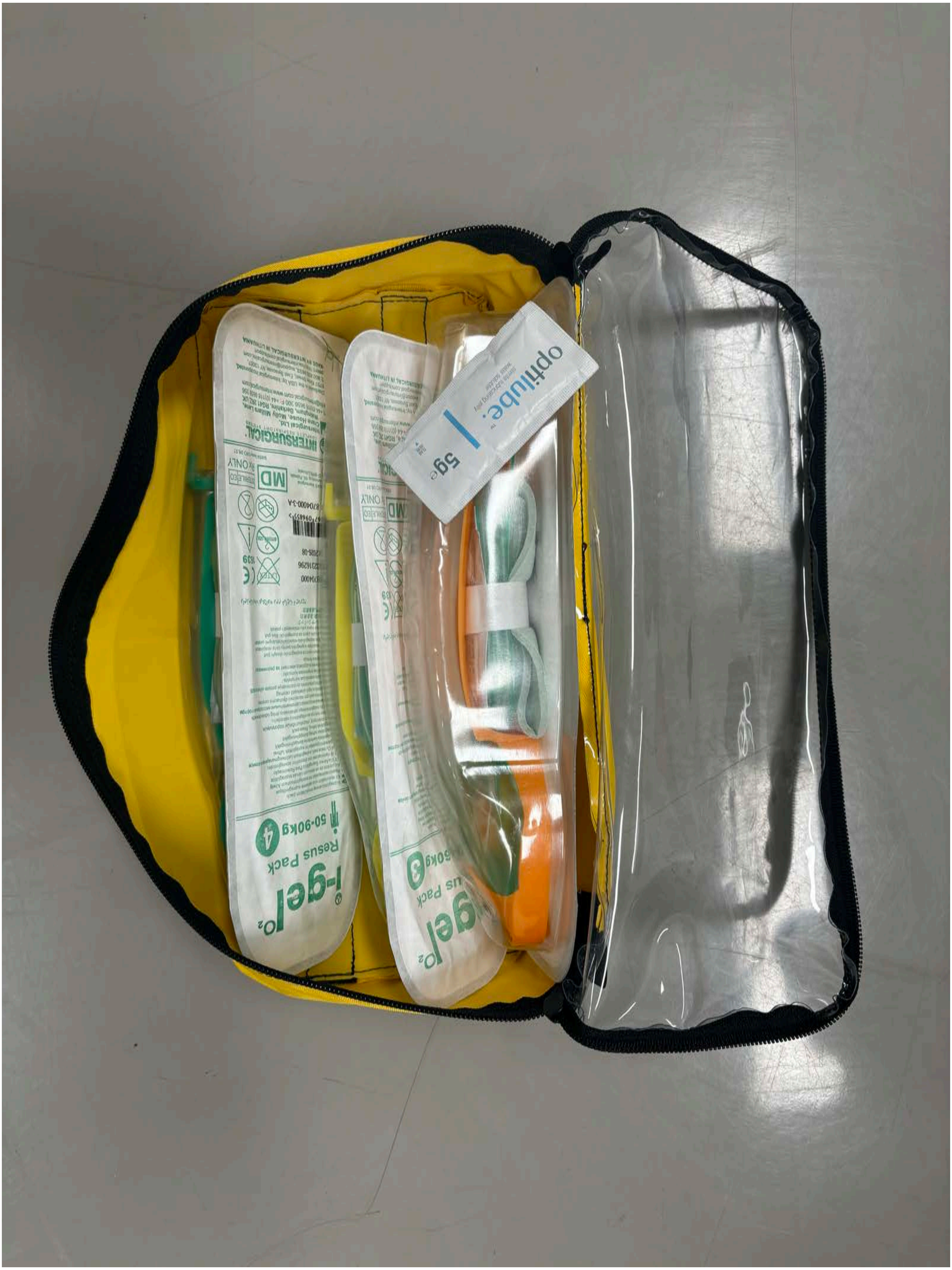
i-gel
Resus Pack
50-90kg
INTERSURGICAL
5g

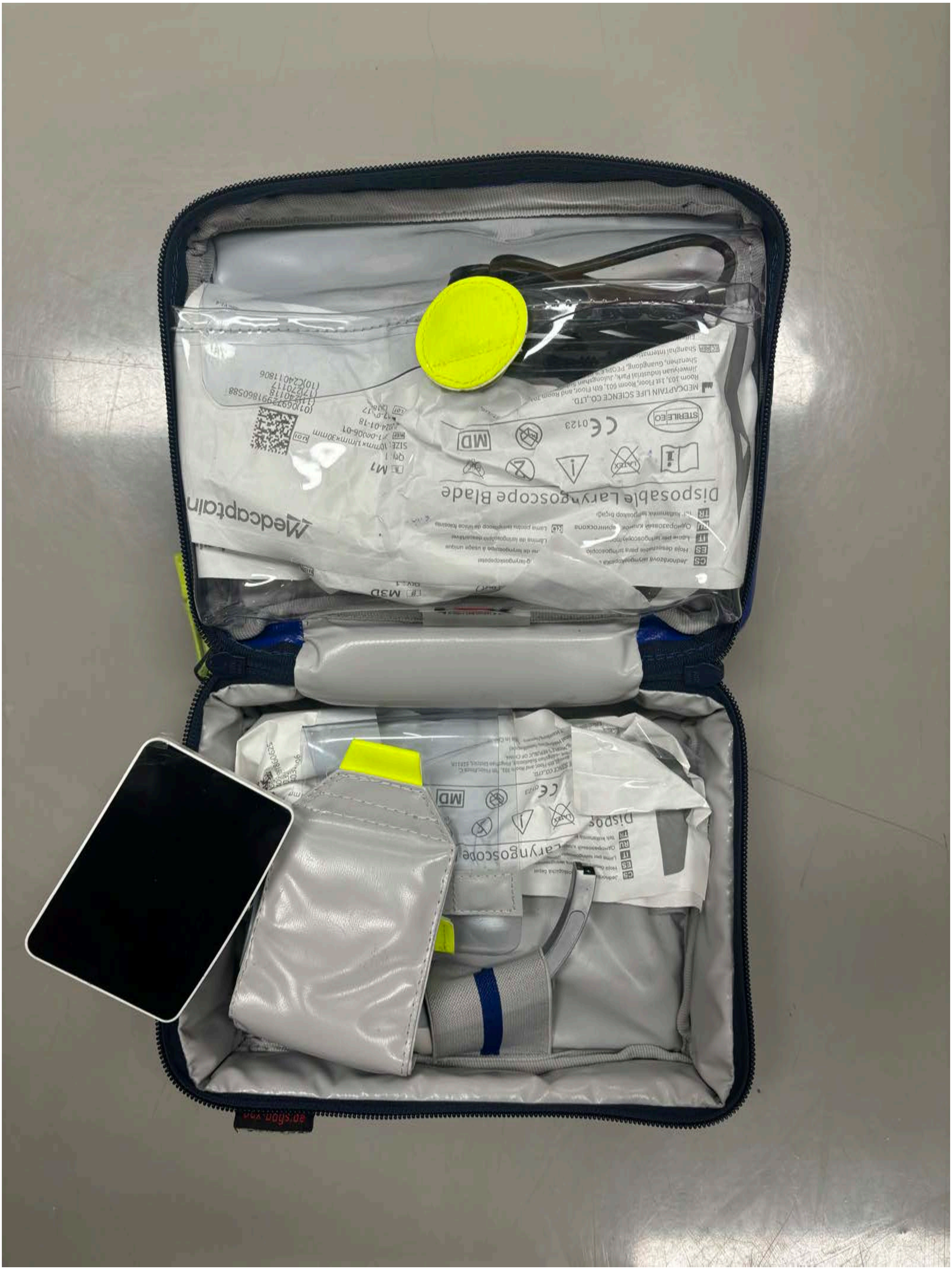
Disposable Laryngoscope Blade
Medcaptain
MEDCAPTAIN LIFE SCIENCE CO.,LTD.
STERILE EO
0123
MD

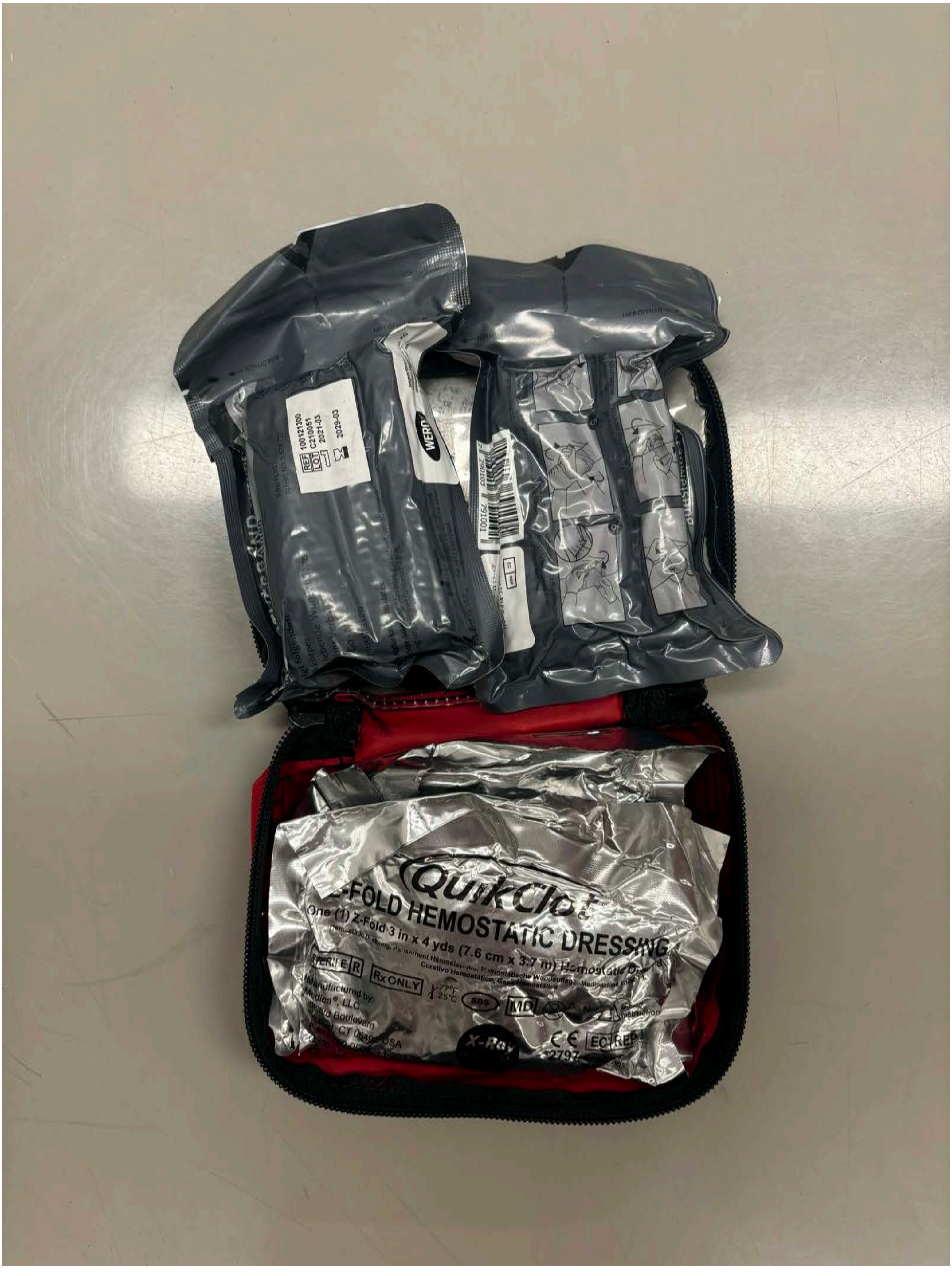

REF 100121300
LOT C210051
2021-03
2029-03
QuikClot
FOLD HEMOSTATIC DRESSING
One (1) Z-Fold 3 in x 4 yds (7.6 cm x 3.7 m)
Curativo Hemostático, Gasa Hemostática
Rx ONLY
Manufactured by:
X-Ray

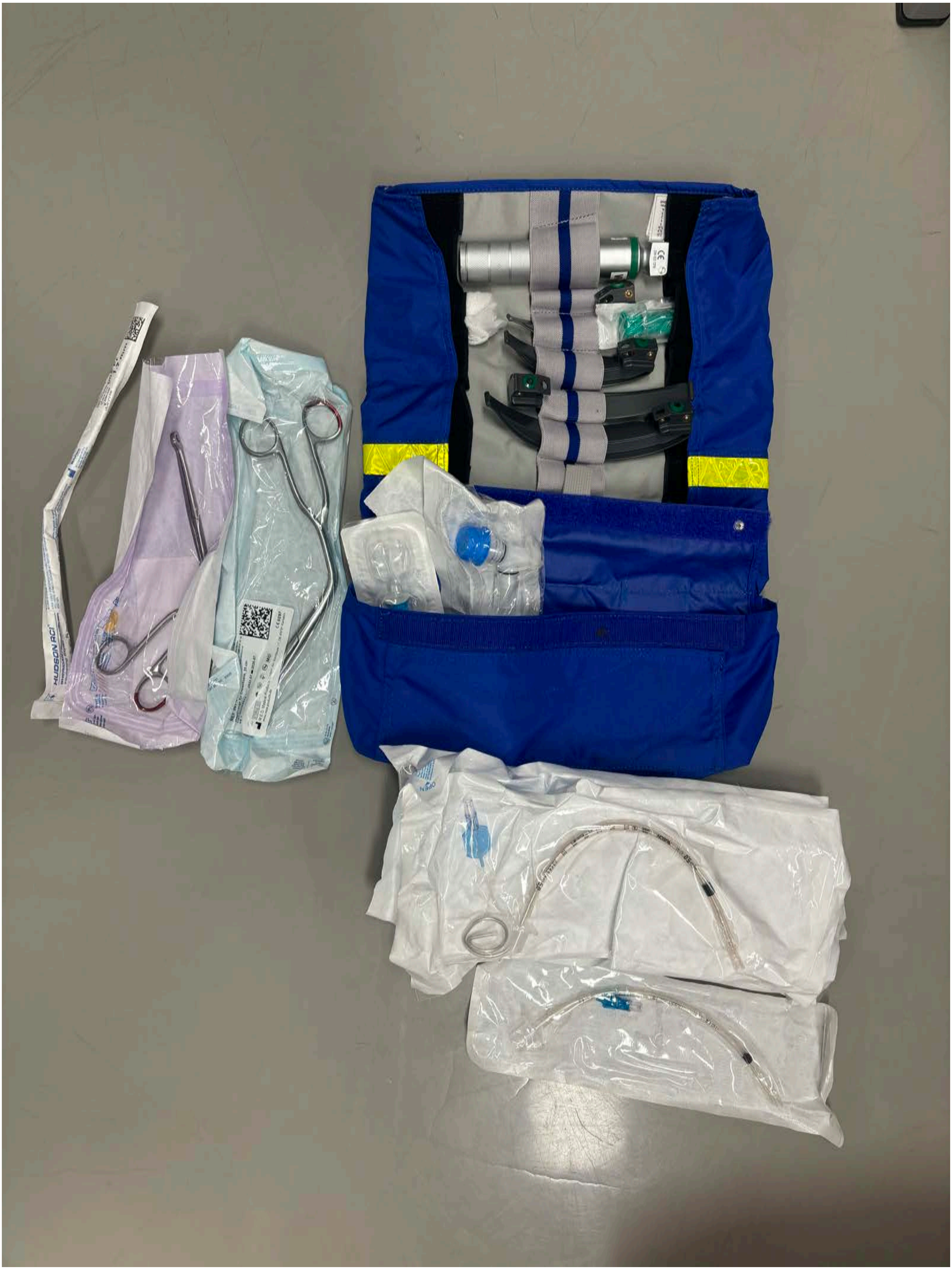
HUDSON RCI

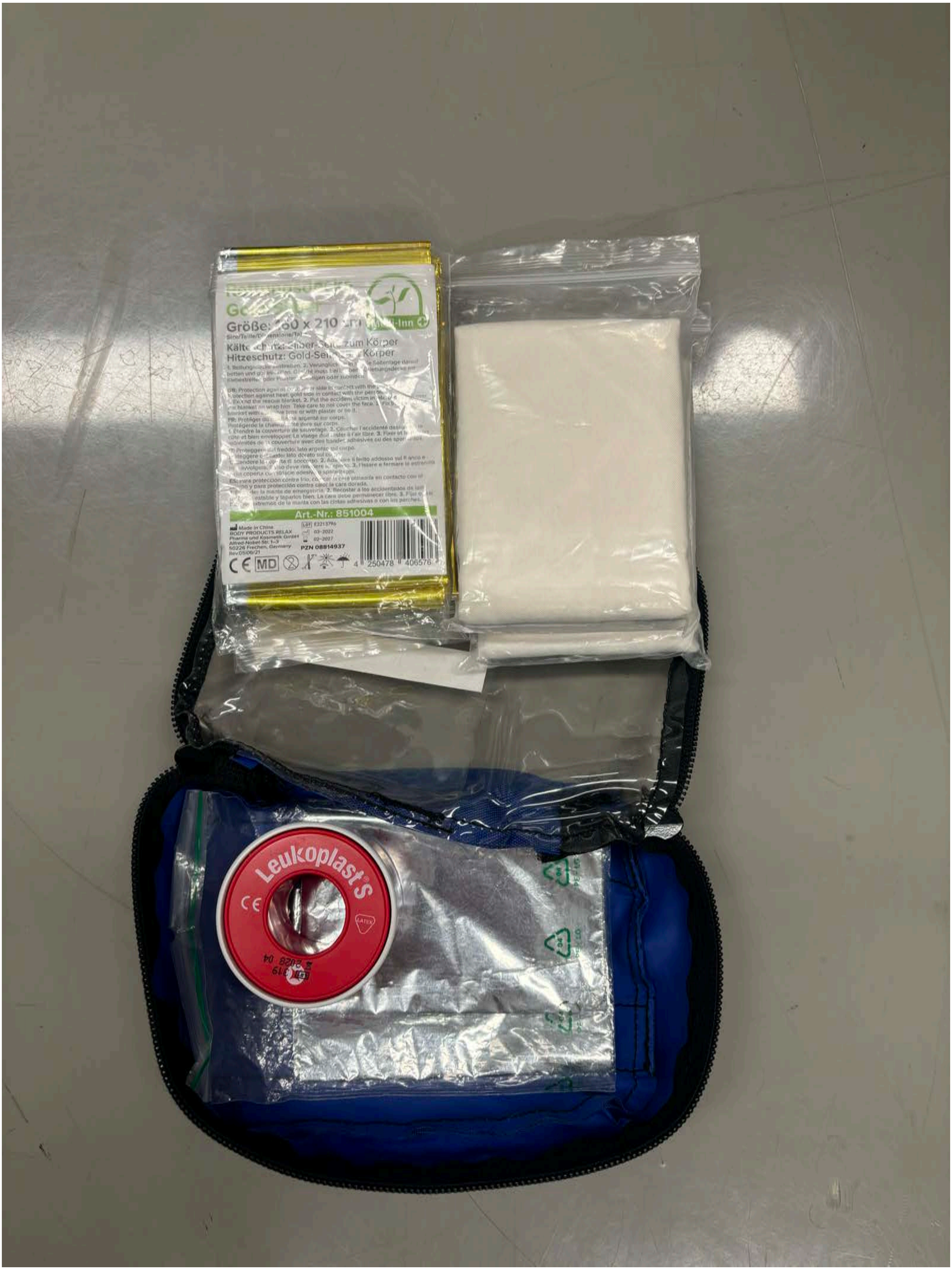
Art.-Nr.: 851004
Made in China
BODY PRODUCTS RELAX
Pharma und Kosmetik GmbH
Alfred-Nobel-Str. 1–3
50226 Frechen, Germany
Rev.05/06/21
E2213796
03-2022
02-2027
PZN 08814937
MD
Leukoplast®S
LATEX

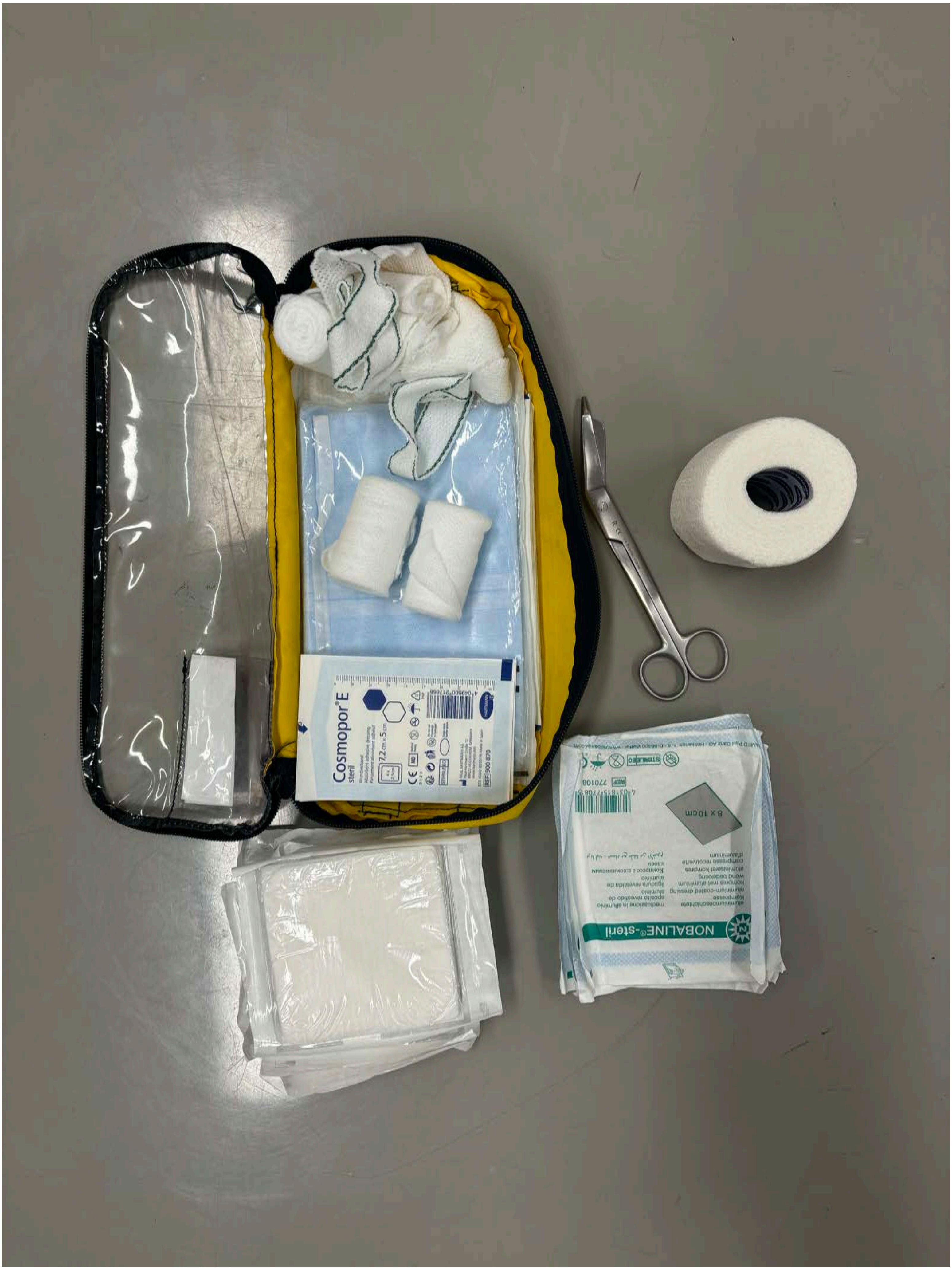
Cosmopor® E
steril
7,2 cm x 5 cm
REF 900 870
NOBALINE®-steril
8 x 10cm
REF 770108

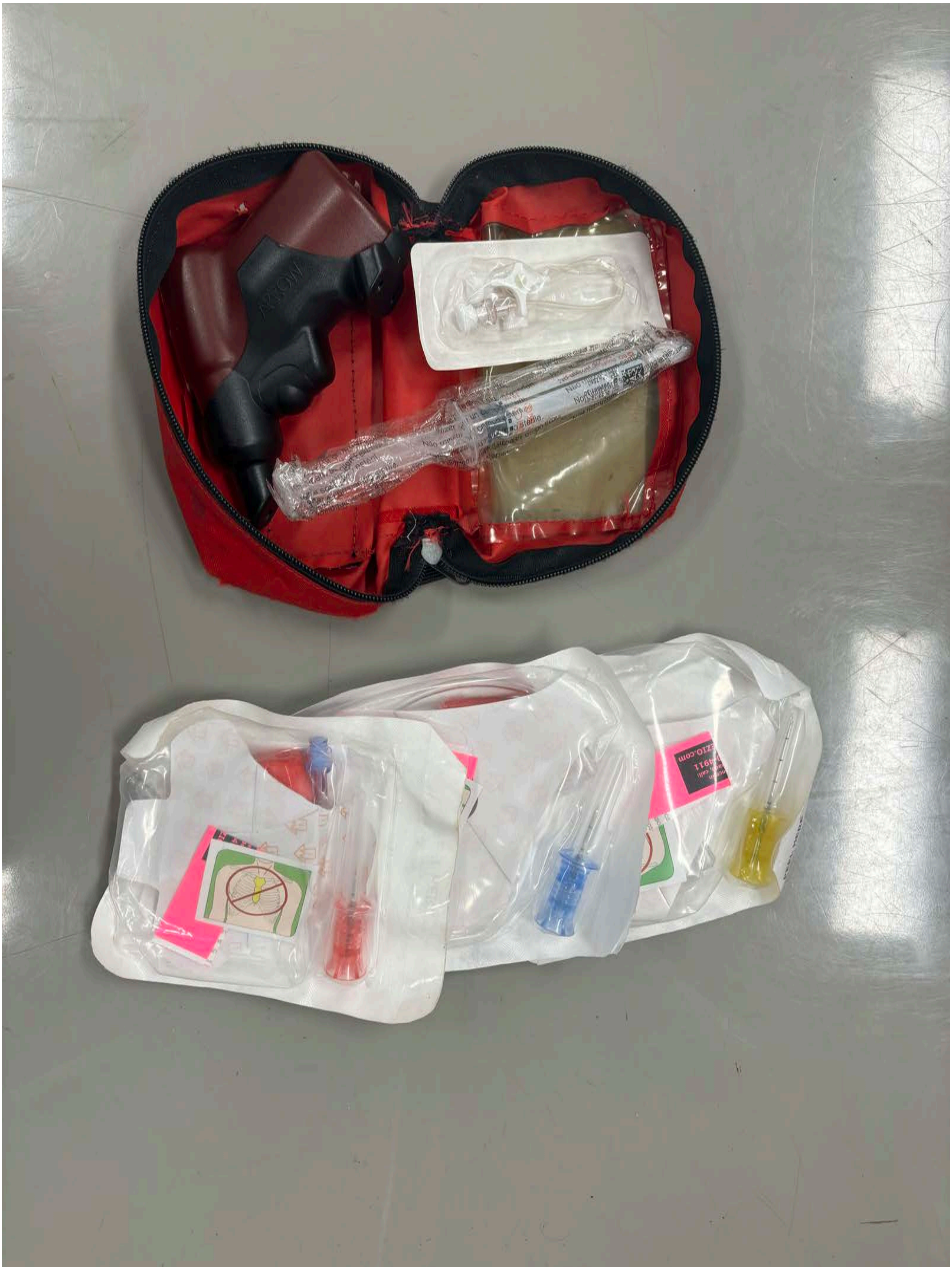

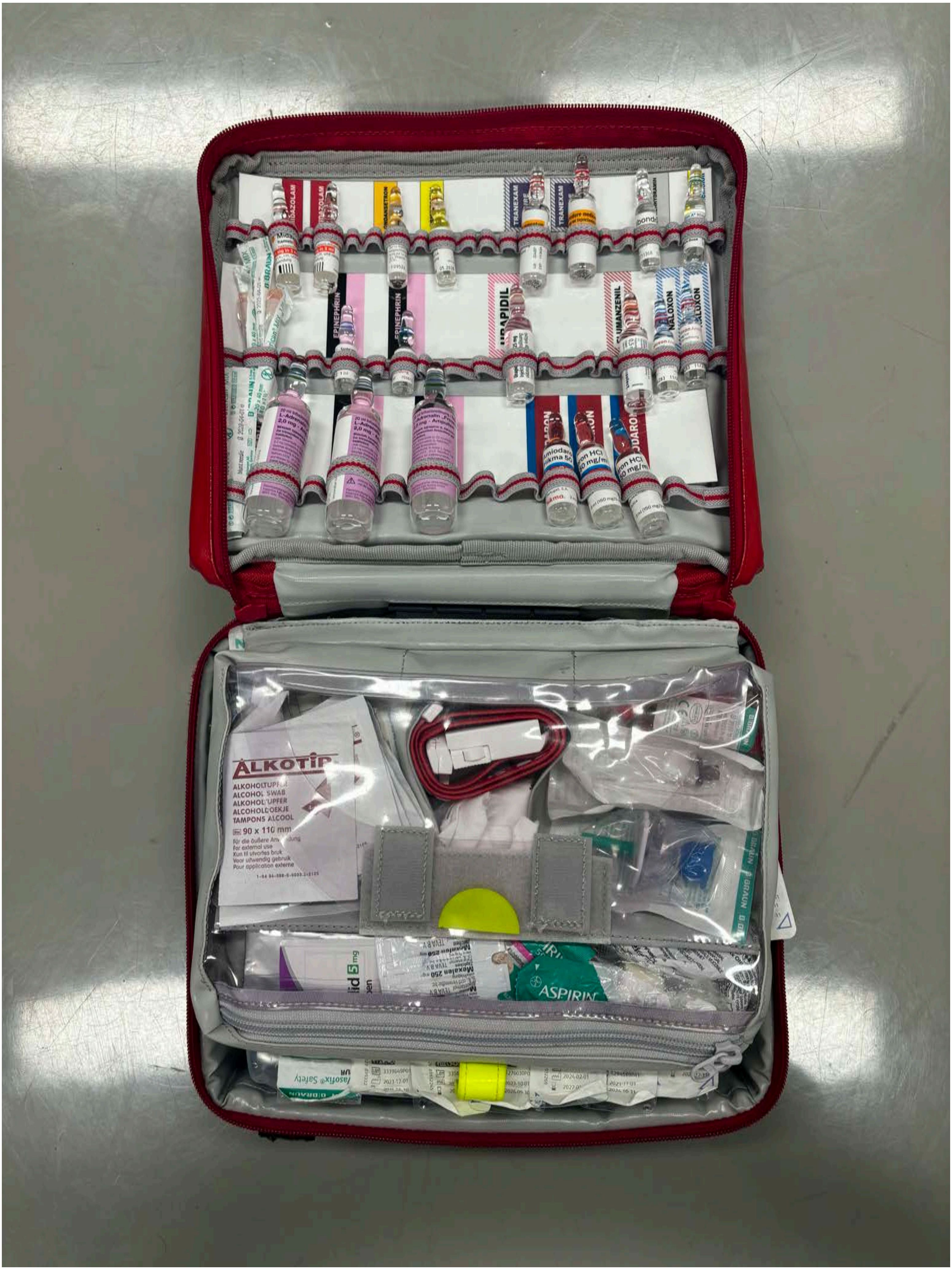
EPINEPHRIN
URAPIDIL
NALOXON
ALKOTIP
ALKOHOLTUPFER
ALCOHOL SWAB
ALCOHOLDOEKJE
TAMPONS ALCOOL
90 x 110 mm
For external use
ASPIRIN

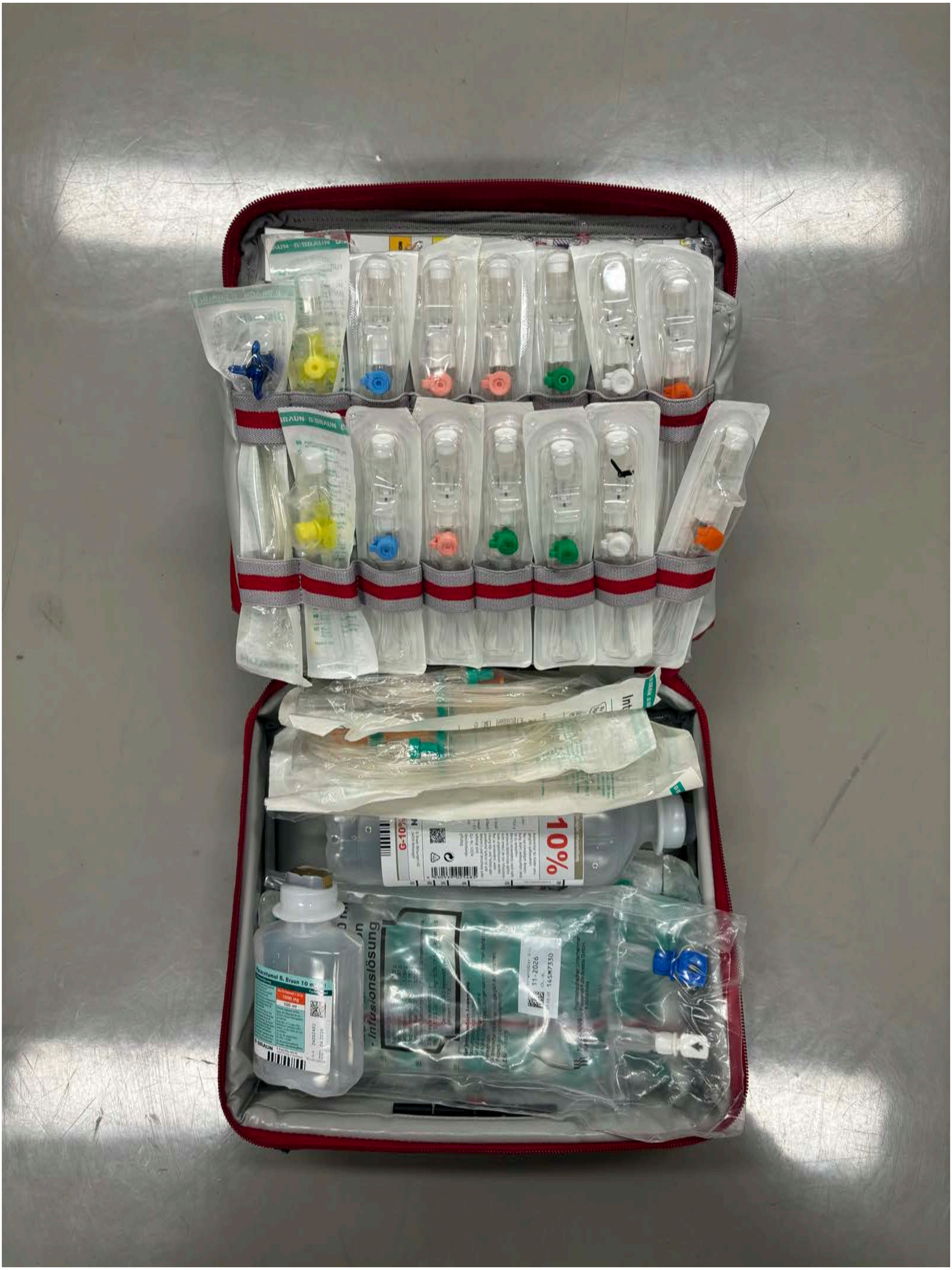
10%
Infusionslösung
11-2026

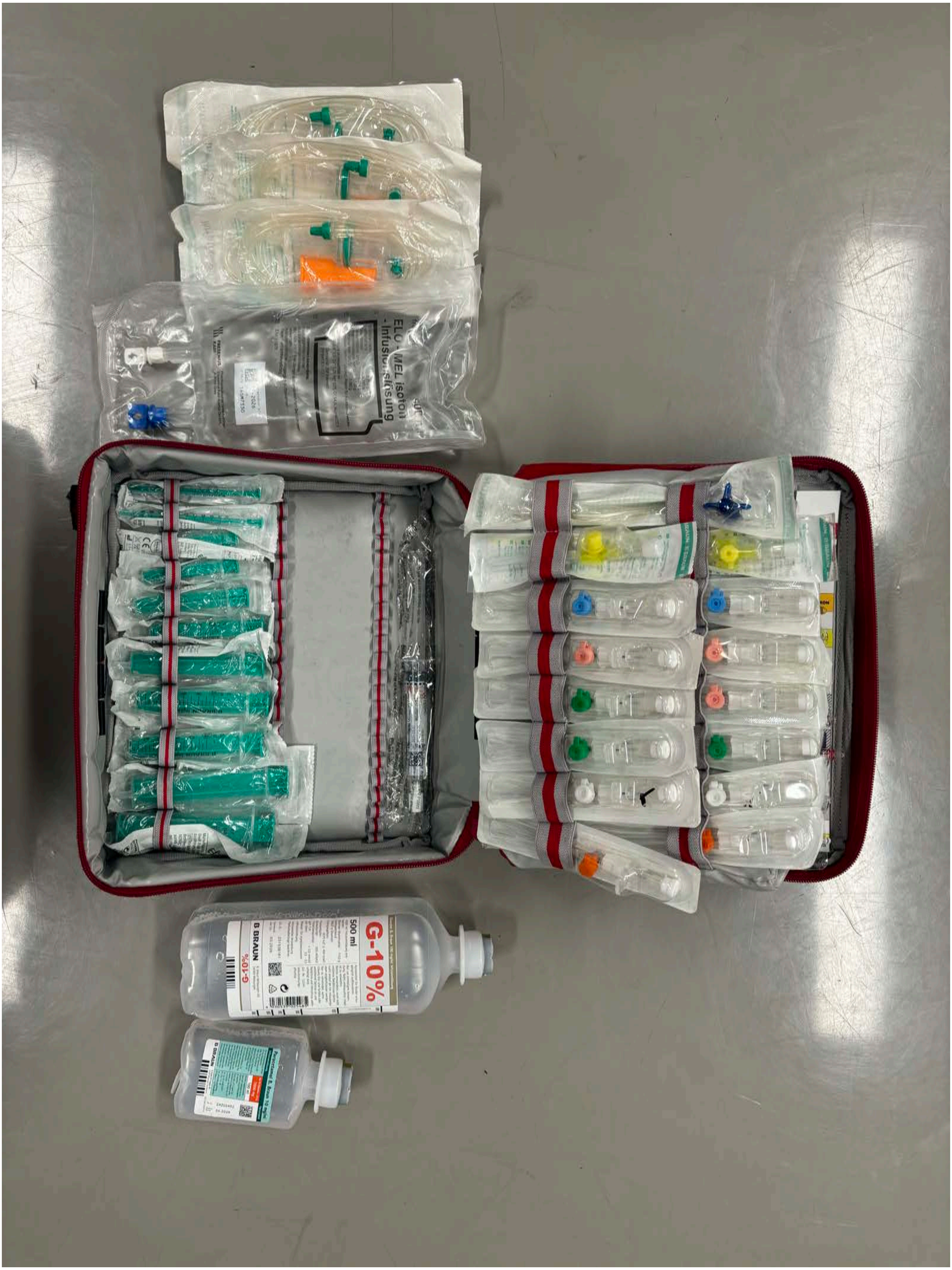
B.BRAUN
G-10%
500 ml

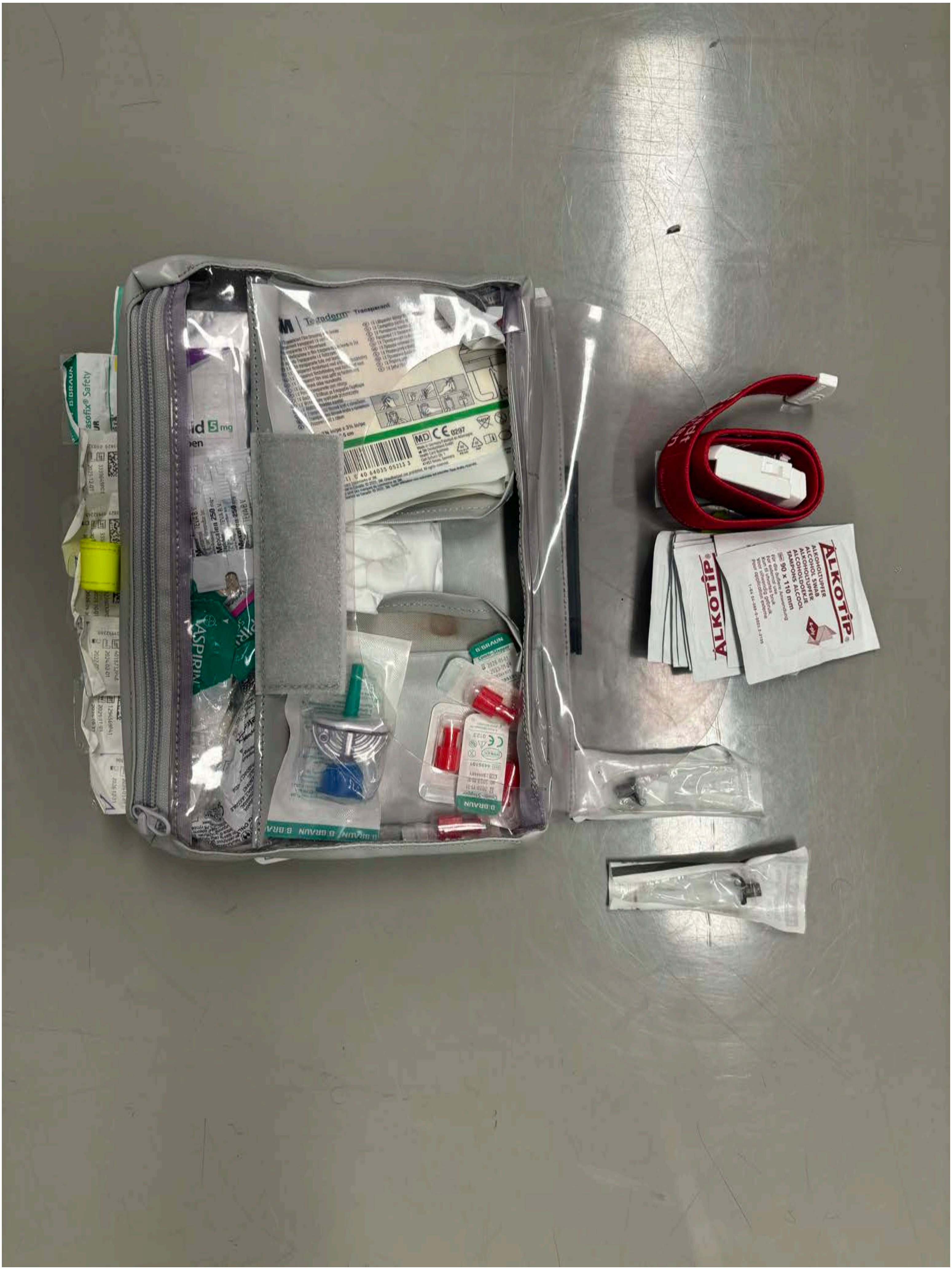
Tegaderm
Transparent
B.BRAUN
ASPIRIN
ALKOTIP
ALKOHOLTUPFER
ALCOHOL SWAB
90 x 10 mm

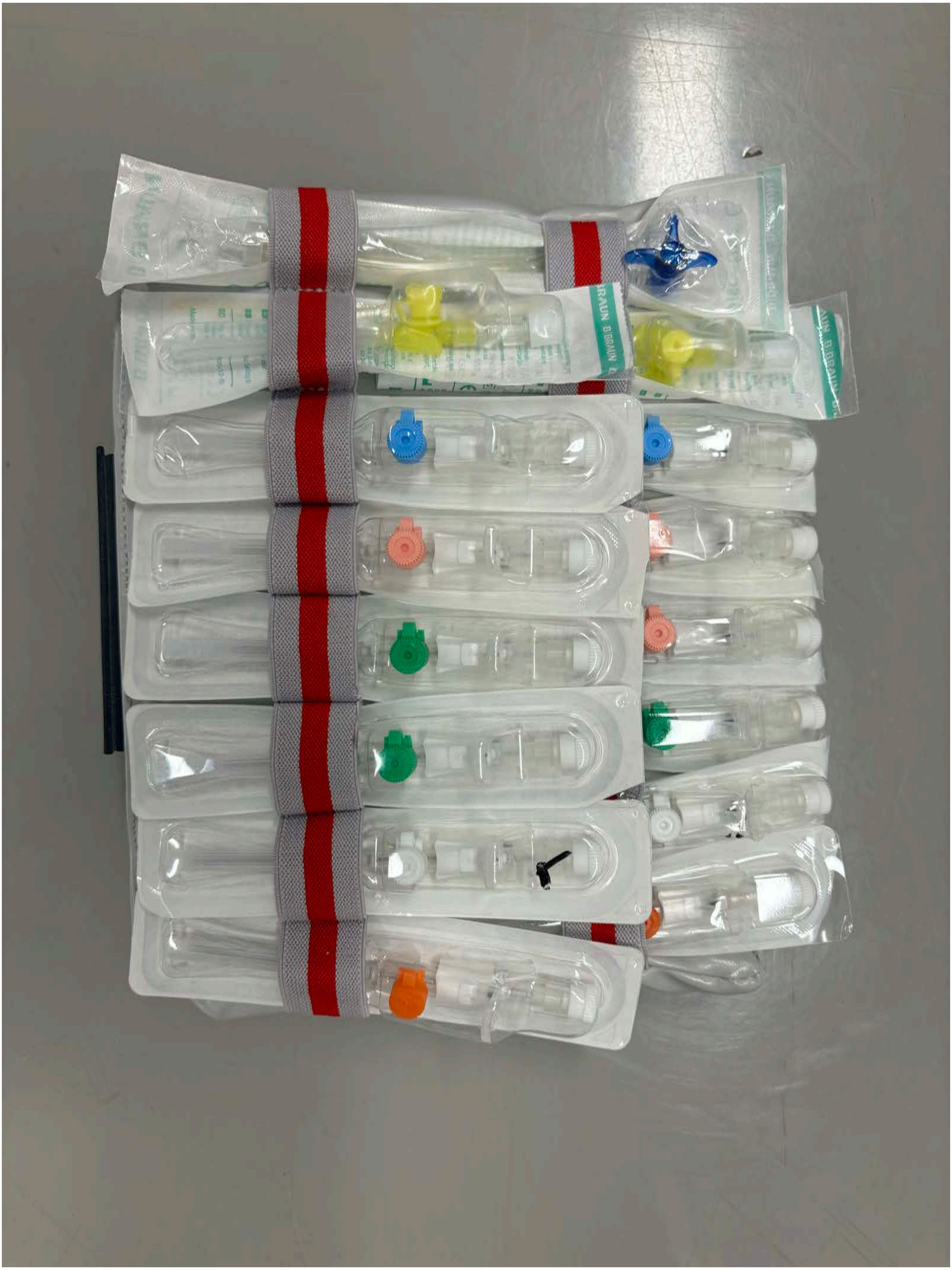
B.BRAUN

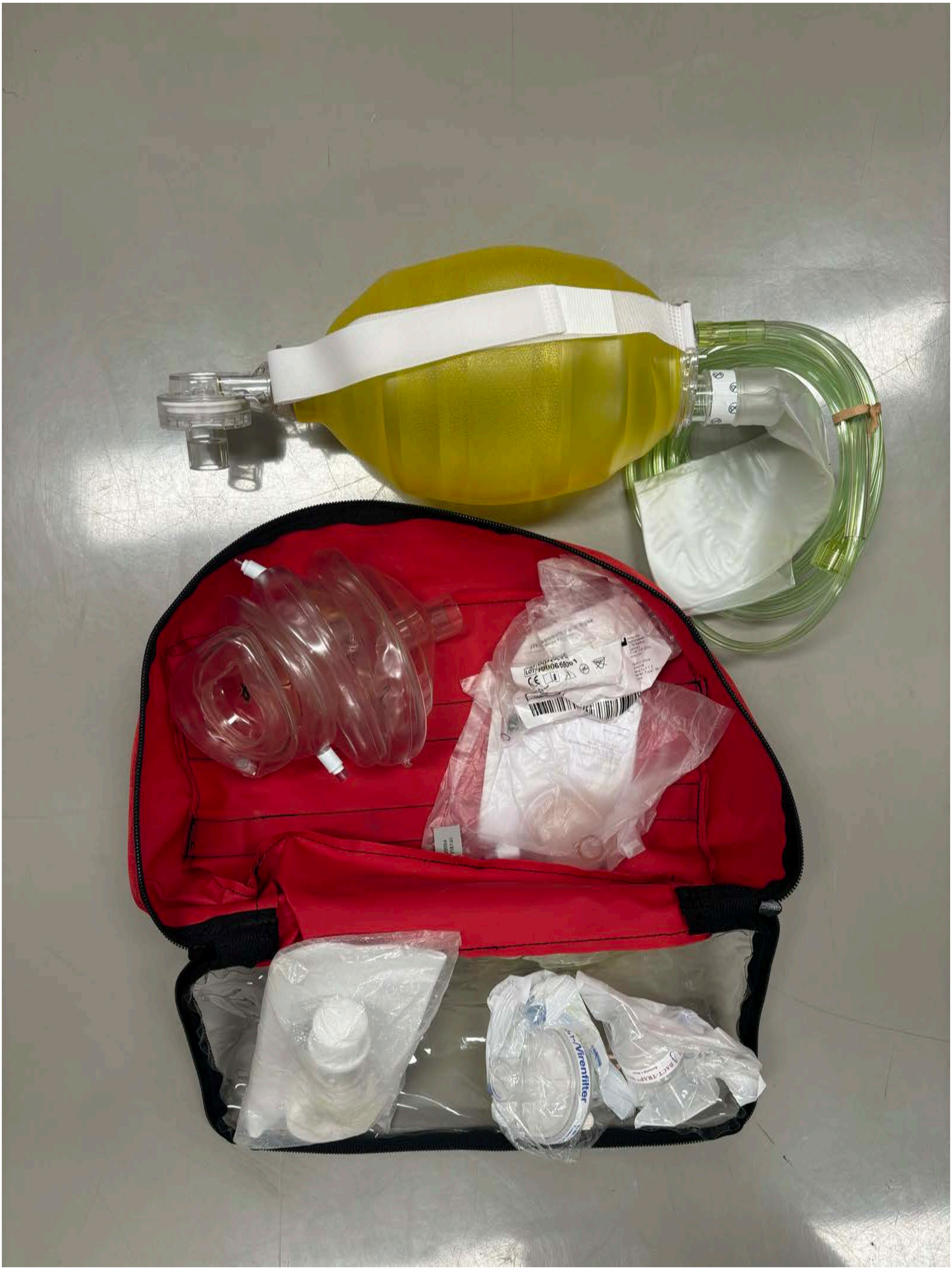

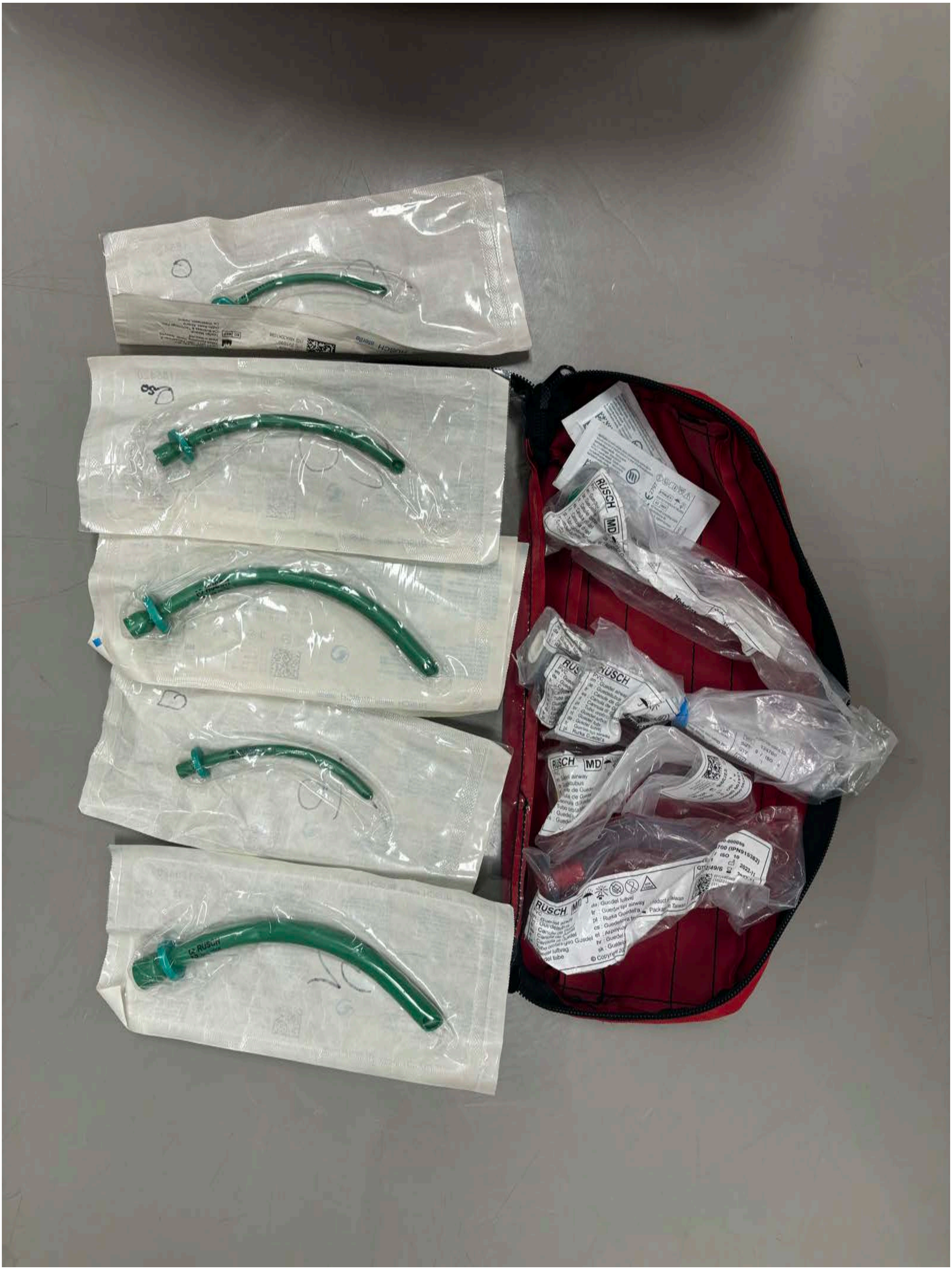
RÜSCH

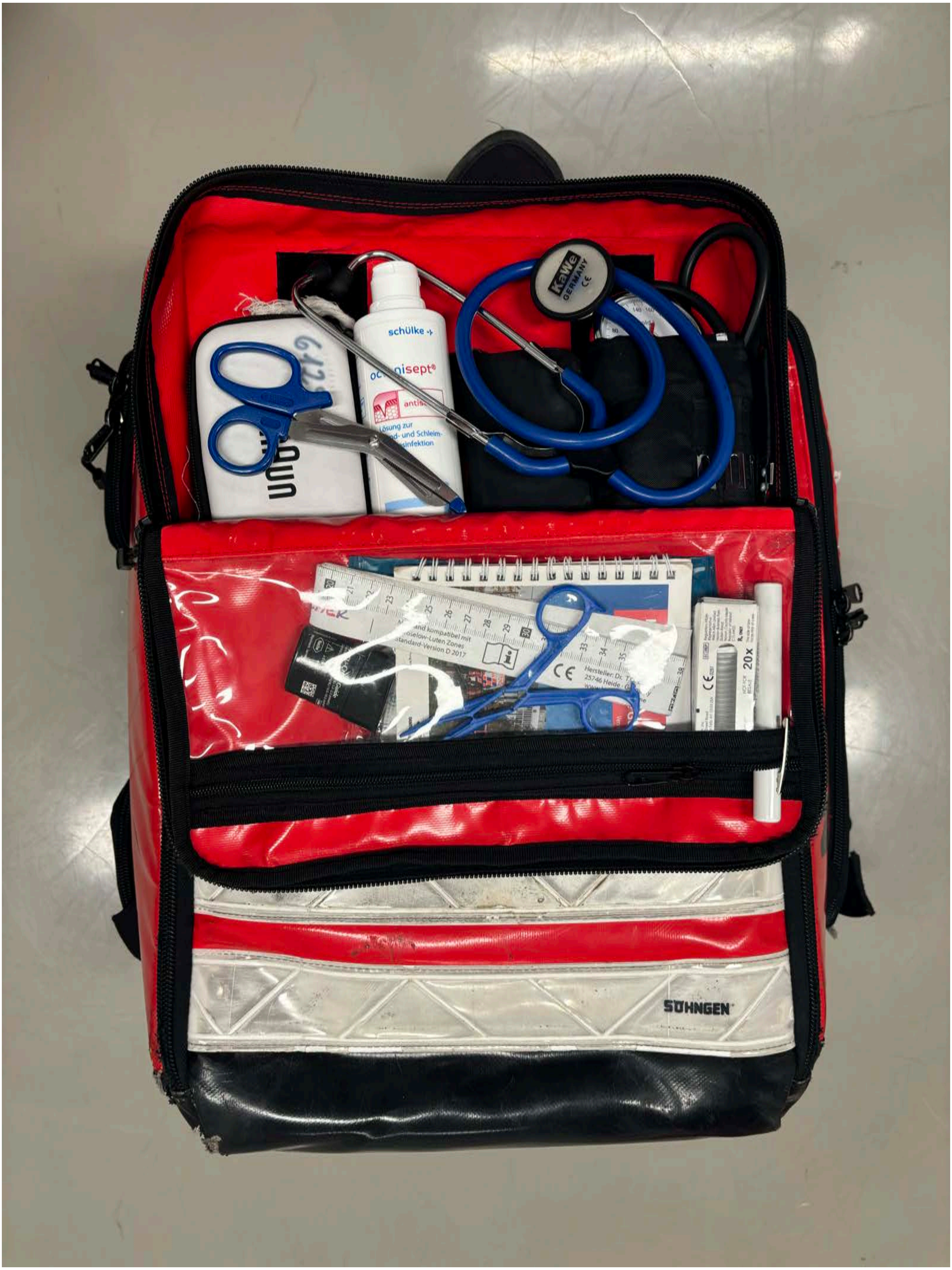
schülke
KaWe
GERMANY
SÜHNGEN

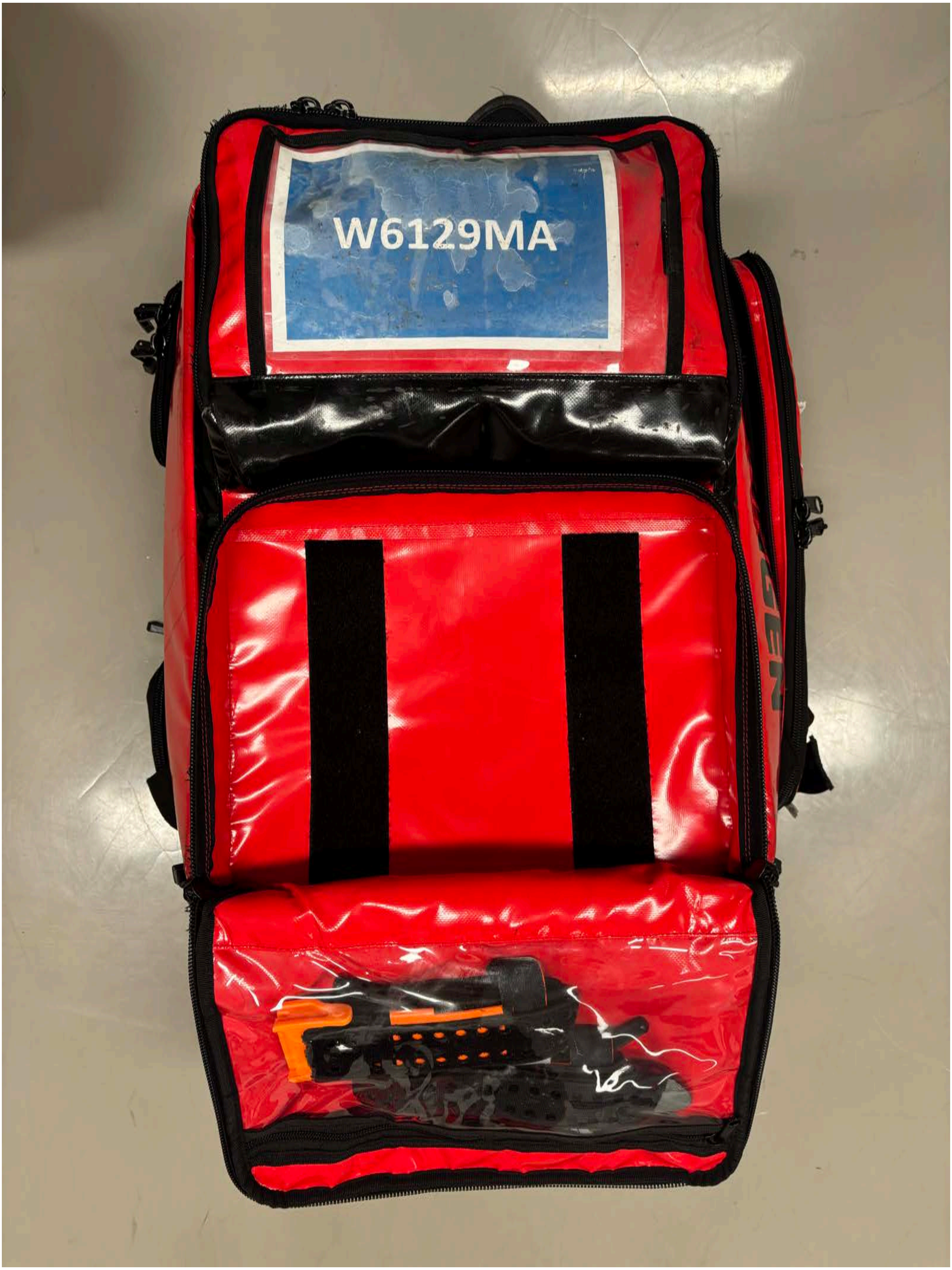
W6129MA
